# Informing Policy Responses to Digital Technology Use and Youth Mental Health and Well-Being: Scoping Review

**DOI:** 10.2196/90160

**Published:** 2026-10-21

**Authors:** Louise Holly, Padmini Vishwanath, Cassie Redlich, Ledia Lazëri, Natasha Azzopardi-Muscat

**Affiliations:** 1 Digital Transformations for Health Lab University of Geneva Geneva Switzerland; 2 Koita Centre for Digital Health Ashoka University Sonipat India; 3 World Health Organization Regional Office for Europe Copenhagen Denmark

**Keywords:** adolescent health, mental health, well-being, social media, digital technologies, health policy

## Abstract

**Background:**

The mental health of children, adolescents, and youths is a growing concern globally, with increasing prevalence of conditions and help-seeking behavior. Alongside traditional factors, digital determinants are now prominent influences on mental health. With digital platforms integral to young people’s lives, and problematic social media use rising, policymakers face the challenge of balancing the benefits and risks of young people’s digital technology use. Decisive action is hindered by inconsistent evidence on the impacts of social media and other technology use on mental health and well-being.

**Objective:**

In response to requests from WHO Europe Member States for guidance on promoting youth mental health in the digital era, this rapid evidence review examines the complex relationship between digital technology use and youth mental health and well-being, with a focus on social media. The aim of the review was to inform recommendations for policy action and support the activities of the Polish Presidency of the European Council in 2025.

**Methods:**

A systematic search of PubMed, PsycNET, and Google Scholar was performed for relevant studies published between January 2020 and December 2024 following a scoping review protocol. Eligibility criteria focused on young people (aged up to 24 years), personal digital technology use, mental health outcomes, English language, peer-reviewed, and conducted within the WHO Europe region or comparable countries. Data were extracted and analyzed using thematic analysis, with findings validated through an expert consultation workshop.

**Results:**

Of 7420 studies, 419 were selected for full-text screening, and 226 (63 reviews and 163 individual studies) met the inclusion criteria. Content analysis reveals mixed evidence on the impact of social media and other technology use on young people’s mental health, with more frequent associations between technology use and negative outcomes. Outcomes were influenced by factors like type of use, individual vulnerabilities, and technology design. A significant number of moderating and mediating factors were identified in the literature that shape the relationship between social media use and mental health outcomes and influence the extent or direction of its impact. Many studies include recommendations for future research actions to be taken by technology companies, policymakers, caregivers, educators, and clinicians to both protect young people from digital harms and encourage positive uses of technology.

**Conclusions:**

Despite methodological limitations in current research, there is growing evidence of digital harms and plausible risks to youth mental health, reinforcing the need for a precautionary policy approach. The findings from this review emphasize the importance of balanced governance that protects youths while allowing them to benefit from technology. This evidence review informed 8 recommended policy actions for European governments, including stronger regulation of technology companies, enhanced digital literacy, and continued longitudinal research to inform effective, youth-centered public health interventions.

## Introduction

### Background

Factors impacting the mental health of children and young people are rapidly attracting the interest of policymakers across the WHO European Region and globally, given the increasing prevalence of mental health conditions and help-seeking among this population observed at the European regional level and internationally [1]. Alongside traditional determinants of mental health for young people such as family environment, emerging determinants including climate change, unemployment, and housing affordability are gaining prominence in young people’s lives [2]. In particular, the rising use of digital media and devices by children and young people has generated significant concerns about the effects of digital determinants*—*defined as the characteristics, features, uses, and governance of digital technologies and digital ecosystems that influence health-related behaviors, health outcomes, and health care delivery—on mental health and well-being [3]. Social media and digital platforms are now embedded in the lives of young people, with the potential to influence their social interactions, self-esteem, and mental well-being [4]. Data from the Health Behaviour in School-aged Children survey report from 2021 to 2022 found that 36% of adolescents reported continuous online contact with friends and others, and 11% of adolescents were classified as problematic social media users—up from 7% in 2017-2018 [5].

The need to develop effective, implementable policy responses to help protect young people’s mental health from the potential harms associated with digital technologies—while balancing the benefits they afford for individuals and society—is acknowledged by governments throughout Europe and the world. Yet, decisive action is hampered by a limited and inconsistent evidence base both for the impacts of digital technologies on youth mental health and well-being and for effective policy interventions to prevent avoidable harms [6]. While there is unquestionable need for policymaking to be grounded in evidence, there are equally high potential costs for public health associated with waiting for convincing proof of harm, as demonstrated through previous responses to environmental exposures including lead, tobacco, and asbestos [7]. In the context of scientific uncertainty and indications of plausible harms, adoption of the precautionary principle in policymaking to mitigate risks associated with digital determinants of youth mental health and well-being is critical and warranted [8].

### Context

This rapid scoping review was initiated by WHO Europe in response to requests from Member States for guidance on the current state of the evidence on the impact of social media and digital technologies on youth mental health and policy actions to mitigate harms. The review also supported activities of the Polish Ministry of Health under the Polish Presidency of the European Council in 2025, where youth mental health and digital technologies were identified as a priority area for action at the European Union level [9]. Alongside this review, researchers also conducted a policy mapping and analysis to understand how countries in the WHO European Region and internationally are already responding to these issues. Together, the evidence review and policy analysis informed the development of a policy brief to guide evidence-informed policy, programs, and a regulatory environment which is protective of young people’s mental health in the digital world [10].

### Objectives

The objective of this review was to gather, summarize, and critically appraise current evidence on the impact of digital technology use on young people’s mental health and well-being, with a particular focus on social media in order to develop recommendations for policy action.

Four overarching questions guided the evidence review, with several subquestions to facilitate a detailed understanding of the evidence base (Multimedia Appendix 1):

- Research question 1: What research has been conducted about the impact of social media and digital technology use on young people’s mental health and well-being?
- Research question 2: What are the key findings on the relationship between young people’s use of social media and digital technologies and mental health or well-being outcomes?
- Research question 3: What recommendations have researchers made for policy action or further research?
- Research question 4: What are the limitations of the current evidence base?

## Methods

### Study Design

A rapid scoping review method was selected for this evidence review due to the exploratory nature of the research and compressed time frame. Scoping reviews investigate the breadth and scope of available evidence on a topic, providing an overview of the literature and identifying key concepts, gaps, and potential future research directions. Drawing on the scoping review framework developed by Arksey and O’Malley [11], and on best-practice PRISMA (Preferred Reporting Items for Systematic Reviews and Meta-Analyses) protocols, we used structured and systematic approaches to provide an overview of the current body of literature [12]. Given the time constraints for this review and our decision to include both reviews and individual studies, it does not meet the formal requirements of a traditional scoping review or review of reviews (see the Limitations section). A PRISMA-ScR (Preferred Reporting Items for Systematic Reviews and Meta-Analyses Extension for Scoping Reviews) checklist was completed to document reporting against applicable items (Multimedia Appendix 2).

A study protocol was developed in November 2024 for a review of evidence on the impact of social media and digital technology use on young people’s mental health and well-being and identification of possible policy implications. The review was carried out by 2 researchers (LH and PV) between December 2024 and February 2025. Ongoing supervision and technical support were provided by members of the WHO Regional Office for Europe’s mental health team and the Digital Transformations for Health Lab.

### Eligibility Criteria

#### Study Population

We included studies that explicitly focused on children, adolescents, or youths. The World Health Organization defines youths as aged 15 to 24 years, so we similarly adopted 24 as the upper age range for study inclusion. We excluded studies focused only on adult populations or that did not explicitly mention children, adolescents, or youths.

#### Type of Social Media Use

Studies were included that explored social media use or the use of digital devices for personal and entertainment purposes such as streaming videos, playing games, or connecting with friends. We excluded studies on the use of digital technologies in schools or for educational purposes. We also excluded studies on digital interventions to treat mental health conditions.

#### Type of Mental Health and Well-Being Outcomes

Included studies explored mental health or well-being outcomes related to technology use. We excluded studies that were exclusively on physical health, developmental outcomes, and nonhealth-related impacts of technology use. Inclusion criteria for types of studies were broad to include empirical studies and all types of reviews published in peer-reviewed publications.

#### Duration of Study

We excluded studies that were exclusively about the impact of technology use during the COVID-19 pandemic due to the unique circumstances associated with this period. Studies published before January 2020 were excluded since older studies would be less likely to reflect current social media use behaviors. The inclusion of evidence reviews (which typically synthesize primary research published over a longer period than our own search window) was intended to capture key findings from studies published prior to this date. The rationale and limitations of this approach are discussed further in this section and the Discussion section.

#### Geographical Scope

Only studies conducted within the WHO Europe region or in countries with comparable levels of digital technology adoption and issues associated with young people’s mental health and social media use (such as Australia, Canada, and the United States) were included.

#### Study Types and Language

Peer-reviewed individual (primary) studies and evidence reviews (including systematic reviews, meta-analyses, scoping reviews, and other review types) were all eligible for inclusion, without applying separate eligibility criteria, search strategies, or analytic approaches to each evidence type. This decision reflected 2 considerations. First, given the exploratory, policy-oriented aim of this rapid review, we sought to map the totality of the available evidence base on this fast-moving topic, rather than to synthesize a single evidence type in isolation; both primary studies and reviews offered relevant and complementary perspectives on the relationship between technology use and youth mental health. Second, because our own search was restricted to studies published between January 2020 and December 2024, including evidence reviews allowed us to draw on synthesized findings from primary research conducted well before this window. This combined approach differs from that of a formal review of reviews, which requires distinct research questions, eligibility criteria, and analytic strategies for reviews versus primary studies, including a formal assessment of overlap between included reviews (eg, using the corrected covered area) to identify primary studies that are indexed more than once. We did not apply these steps, and this is recognized as a limitation of our approach in the Discussion section. Other forms of literature such as commentaries or gray literature were excluded. Only studies published in English were included.

### Search Strategy and Selection Criteria

Our search strategy was based on combining 3 groups of terms: population terms, technology terms, and outcome terms (Multimedia Appendix 1). We ran our initial searches in PubMed, PsycNET, and Google Scholar on November 29, 2024. These 3 databases were selected because they cover both health-specific and interdisciplinary academic fields. We supplemented our systemic search with references from previous research studies and through citation searching. A PRISMA flow diagram was used to keep track of records.

### Data Extraction and Analysis

We used a thematic analysis approach to extract and analyze data from the selected studies. Data were recorded in a Microsoft Excel spreadsheet, which organized data according to the research questions. After all relevant information had been extracted from the studies, the content of the studies was analyzed for recurrent themes and notable findings. Data were exported to a Microsoft Word document to enable descriptive and narrative synthesis.

Emerging themes from the data analysis were shared at regular intervals with the wider project team from the World Health Organization and the Digital Transformations for Health Lab. Preliminary findings from the study were also presented for feedback and validation at an expert consultation workshop with European and global experts convened by WHO Europe in February 2025. Based on the feedback from this meeting, data related to evidence quality in the included studies were revisited, and additional thematic analysis was conducted.

## Results

### Overview

Our search revealed that a huge volume of literature has been published on issues related to the health and well-being impacts of young people’s technology use over the 5-year period of 2020 to 2024.

Our initial literature search yielded 7420 articles. Title screening, abstract screening, and deduplication were conducted using the Rayyan platform [13]. Two researchers (LH and PV) screened titles and abstracts in Rayyan to identify 419 records for full-text screening. Full-text screening against inclusion and exclusion criteria was conducted in parallel by the 2 reviewers (LH and PV), who met frequently during the screening period to ensure consensus on which studies met the inclusion criteria and to resolve any disagreements. This process resulted in a total of 226 articles being selected for data extraction and analysis (Figure 1) [14,15].

**Figure 1:**
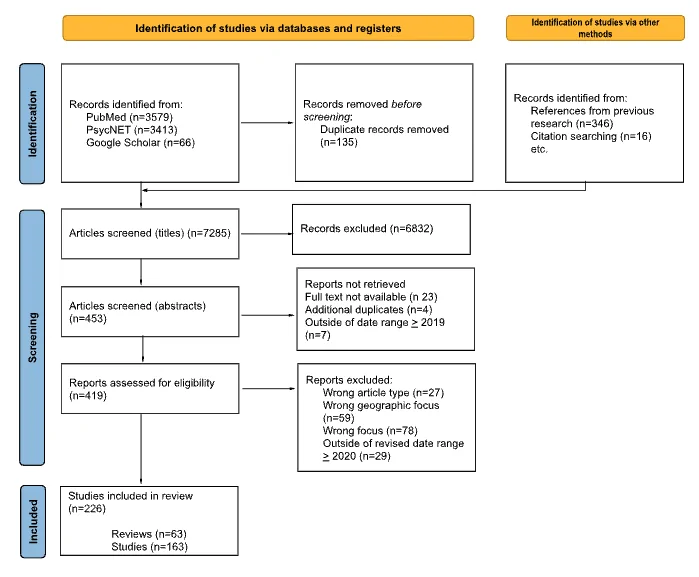
PRISMA (Preferred Reporting Items for Systematic Reviews and Meta-Analyses) flowchart.

### What Research Has Been Conducted About the Impact of Social Media and Digital Technology Use on Young People’s Mental Health and Well-Being?

Of the 226 included articles published, 63 were reviews, and 163 were individual studies. The individual studies included 107 cross-sectional studies and 37 longitudinal studies (Tables S1 and S2 in Multimedia Appendix 3). The large proportion of reviews within this sample reflects the high volume of research activity on this evolving topic as well as uncertainty and disagreement over how to interpret evidence (Figures 2 and 3).

**Figure 2:**
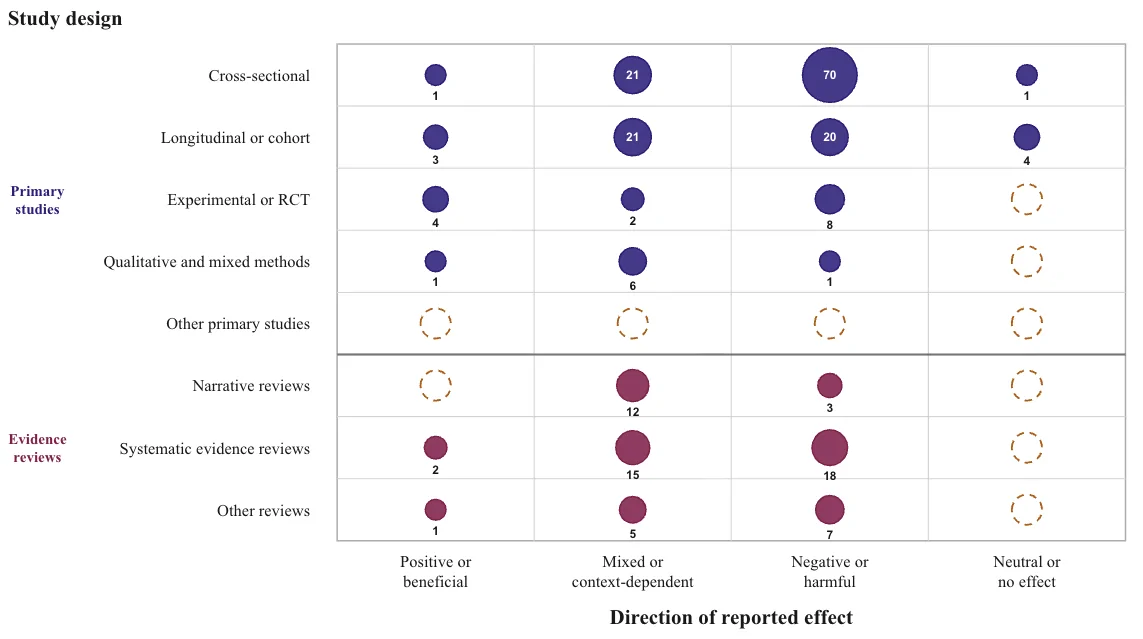
Evidence gap map: study design and the direction of reported effects (2020-2024). 226 studies reporting associations between digital exposure and mental health among young people, cross-tabulated by study design (rows) and the direction of the reported effect (columns). Bubble area and labelled values indicate the number of studies for each cell; colour denotes primary studies (indigo) versus evidence reviews (wine). RCT: randomized controlled trial.

**Figure 3:**
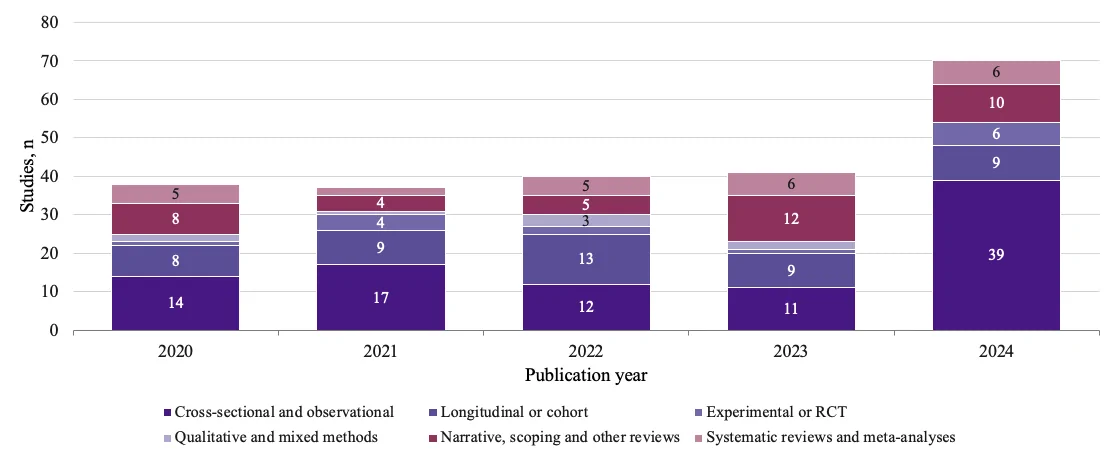
Evolution of study designs (2020-2024). Number of studies investigating digital exposure and mental health outcomes among young people, by study design and publication year. RCT: randomized controlled trial.

### Focus of Research

#### Digital Exposures

Content analysis revealed significant diversity in the focus of the research published over a 5-year period (Table 1 and Figure 4). Young people’s general use of social media platforms was the dominant area of inquiry but, within this theme, a wide variety of subthemes were recorded. Many studies looked at the quantity of young people’s social media use, often using measures like screen time to assess problematic technology use or addiction. Other studies, particularly the more recent ones, emphasize the need to examine the type or nature of young people’s social media use, rather than how much time they spend on the platforms. Several studies concentrated on specific digital behaviors such as communication with strangers, information seeking, cyberbullying, or content generation.

**Table 1:** Types of digital and social media uses studied.

Theme	Description and terms used	Specific use types
Problematic or addictive or excessive use	Quantity of use; addiction; time of use; screen time; problematic internet use	Excessive use; addictive use; compulsive use; night-time use; prebedtime use
Type of social media use	Nature of social media use: passive; active; social; nonsocial; visual	Posting exaggerated materials; watching or scrolling; influencers; likes or comments; social media trends; oversharing; experiencing triggering posts; viewing or exposure to idyllic body images; viewing body positivity; sharing selfies; using filters or editing photos
Communication	Communication between users who know each other; communication with strangers	Sharing messages, content, videos, or images; sexting or sexualized communication
Harmful content or threatening behaviors	Crimes or content that have clear harmful implications	Cyberbullying; victimization; sex trafficking or harassment; pornography; self-harm content; disordered eating content; violent content
Information seeking or dissemination	Information seeking or dissemination on various topics, such as mental health	Seeking mental health information; connecting with others on mental health; peer support groups; nutritional knowledge
Other entertainment	Other digital technologies that are not related to social media use	Gaming, gambling; television; video watching; streaming

**Figure 4:**
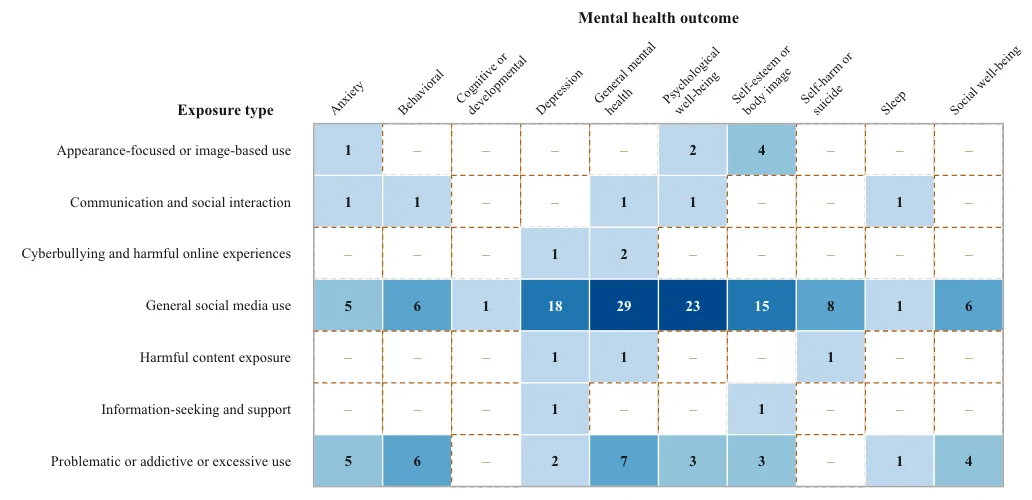
Evidence map: primary digital exposure types and primary mental health outcomes of included individual studies (2020-2024). Total of n=163 coded exposure–outcome pairings across k=226 studies; a single study could be coded against more than one outcome. Cell shading intensity indicates the number of studies; dashed cells indicate combinations for which no studies were identified, and lightly shaded cells indicate sparse, low-frequency evidence.

#### Mental Health and Well-Being Outcomes

A spectrum of mental health and well-being outcomes was also studied in the included literature, from clinical mental health disorders such as depression to mental health challenges that impact young people’s daily lives and well-being such as stress, loneliness, and belonging (Textbox 1). While the impact of technology use on young people’s cognitive and brain development was not an explicit inclusion criterion for this review, these themes were identified in much of the literature.

**Types of mental health and well-being outcomes studied.**

**Intermediate factors shaping mental health or well-being outcomes**

- Peer comparison
- Social upward comparison
- Appearance-related consciousness
- Feedback seeking
- Suicidal contagion
- Sleep
- Fear of missing out
- Nomophobia
- Body or image dissatisfaction
- Addiction
- Overeating or unhealthy eating
- Obesity
- Thwarted belongingness
- Fatigue
- Irritability
- Apathy
- Racing thoughts
- Rumination
- Declining grades

**Overall mental health or well-being impact**

- Depression and anxiety
- Psychological and emotional well-being and affect
- Social well-being
- Suicide or self-harm
- Behavioral or developmental problems
- Others

**Specific types of mental health and well-being outcomes**

- Clinical depression or anxiety
- Self-reported depression or anxiety based on scale
- General well-being, negative affect, sadness, guilt, momentary affective well-being, life satisfaction, self-esteem and confidence, resilience, emotional problems (strengths and difficulties), boredom, stress, personal identity
- Loneliness and isolation, perceived social support, social connectedness, belongingness, social craving, peer relationship problems
- Suicide, suicidal ideation, suicide attempts, self-harm
- Psychosocial development, conduct problems, hyperactivity or inattention, self-control, sensation seeking, aggression
- Cognitive development, brain development, school satisfaction, learning outcomes

#### Intermediate Factors

We also identified a wide range of outcomes that, while not mental health and well-being outcomes themselves, preceded and influenced these final outcomes in young people’s technology use. We refer to these factors as intermediate factors (Textbox 1).

### Key Findings on the Relationship Between Young People’s Technology Use and Mental Health Outcomes

#### Overview

Social media use has a complex and multifaceted relationship with mental health and well-being, influenced by how, why, and how much it is used. Since a wide variety of mental health and well-being outcomes have been studied in the literature, we classified these outcomes into four categories: (1) depression, anxiety, and suicidality; (2) psychological and emotional well-being; (3) social well-being; and (4) behavioral, physical, and developmental impacts. Much of the evidence on the nature, strength, and direction of the relationship between social media use and the aforementioned outcomes is mixed.

#### Depression, Anxiety, and Suicidality

Some studies show a small, positive association between social media use and depression, whereas others show no or even a negative association [16]. Some evidence suggests a bidirectional relationship, where preexisting depressive symptoms drive increased social media use [17]. The nature of technology use may influence the association with depression and anxiety. For example, passive social media use, online victimization, and appearance-focused content are often linked to negative outcomes [17-22]. An association between social media use and anxiety and suicide disorders has been found, but the magnitude and nature of these relationships are still unclear. [20,21,23-29]. Some studies have shown that cyberbullying and victimization may be key moderators or mediators of the association between social media use and suicidality [30,31]. Exposure to self-harm images, while understudied, has shown some association with suicidal ideation [32,33].

#### Psychological and Emotional Well-Being

Similarly, psychological and emotional well-being may be shaped by the type of engagement with technologies. Evidence on the nature of association between social media use and psychological and emotional well-being is mixed. Passive use and posting appearance-related content have been linked with reduced well-being, while moderate use and positive interactions may enhance empathy and self-esteem [18,34-38]. Viewing idyllic images has been associated with negative well-being, especially among girls and those with internalized norms of body ideals [17,39]. Body positivity content, while understudied, has shown mixed impact on well-being. While there is some potential positive impact, it also poses a risk of triggering body consciousness by initiating thoughts on body image [40]. Alternatively, identity development and other positive impacts on personality growth have been seen through the ability to self-express on social media [41,42]. Overall, digital technology use may be more likely to bring short-term effects as compared to longer-term impacts such as lifelong satisfaction [18].

#### Social Well-Being

Evidence on the nature of association between social media use and social well-being is similarly mixed. While social media offers opportunities for connection and belonging, particularly for vulnerable groups, it carries risks such as loneliness and hate speech [35,43-50]. Studies have shown both positive and negative associations, depending on factors including the type of use and who a user communicates with [44,51]. Social media and other online platforms could provide important avenues for displaced and marginalized youths to foster and sustain intimacy by creating opportunities for exchange and emotional support across distances [52]. Relationships could be bidirectional, as poor social well-being outcomes such as a lack of family support and loneliness may result in increased social media use to fulfill social needs [22].

#### Physical, Developmental, and Behavioral Impacts

Studies on the physical, developmental, and behavioral impacts of technology use also include mixed outcomes, with some young users benefiting from improved problem-solving skills while others face challenges like disordered eating, poor sleep, and increased aggression. These findings also highlight the nuanced effects of social media use, where context and individual factors play critical roles in determining outcomes. The impact of social media and other platforms on eating behaviors is an emerging area of study with links found between use of highly visual social media and unhealthy, disordered eating and overeating [43,53,54]. Insomnia and sleep quality are associated with excessive or time of technology use but not type of use [31,55,56]. Several studies have shown some association between sedentary lifestyles and increased social media use [25,57,58]. Some evidence shows that social media use may be associated with increased aggressive behaviors, sensation-seeking, or other inattention or hyperactivity problems. One study also pointed to the specific role of influencer-generated content or challenges that may exaggerate imitation of dangerous behaviors [59,60]. There may be reverse causality, as some evidence shows that maladaptive behaviors such as inattention and disengagement may be associated with increased social media use [59,61].

### Mediators or Moderators of the Relationship Between Young People’s Technology Use and Mental Health Outcomes

A significant number of moderating and mediating factors were identified in the literature that may shape the relationship between social media use and mental health outcomes and influence the extent or direction (positive or negative) of its impact (Figure 2). Moderators are variables that change the strength or nature of the relationship, such as gender: girls and gender-diverse youths often face worse outcomes, particularly around body image-related content [28,32,34,48]. Mediators, on the other hand, explain how or why social media use affects well-being, such as engagement in upward social comparison leading to depressive symptoms [27,62]. Researchers have extensively studied these factors to understand how and why social media use affects mental health and well-being.

Psychosocial and behavioral factors—like low self-esteem, social anxiety, and excessive investment in online self-presentation—were found to exacerbate negative outcomes, especially among vulnerable groups [44,53,63,64]. Resilience and emotional self-regulation may temper these harms [32,65]. Additionally, motivations for using social media, importance given to peer feedback, and perceived self-control moderate the relationship [16,42,66-69]. Sociodemographic factors such as socioeconomic status, age, and digital literacy also play a key role in moderating risks and benefits, highlighting the complexity of understanding social media’s effects or association with mental health [23,32,49,51,65,70,71].

### Factors That Influence Young People’s Vulnerability and Resilience

#### Overview

Some researchers attribute differences in mental health and well-being outcomes resulting from technology use to young people’s varying levels of vulnerability or resilience [29,65]. This means that some children are better equipped than others to withstand, adapt to, or recover from negative experiences associated with technology use.

Several studies have explored evidence on what factors can contribute to greater resilience (ie, protective factors) or vulnerability (ie, risk factors). Numerous protective and risk factors were identified in the literature and can be broadly categorized into individual factors related to the young digital technology user and environmental factors that reflect what is happening in the young person’s family, community, and society (Table 2). Many of these factors can coexist and interact in complex ways. A frequent observation in studies is that children who are generally exposed to higher levels of adversity tend to have fewer protective factors [72]. Gender and digital literacy were 2 key factors that were identified as both protective and risk factors in different studies [29,34,41,45,65,73-75].

**Table 2:** Protective factors that may increase resilience or vulnerability.

Protective factors			Risk factors
**Individual factors**			
	Individual temperament (eg, self-confidence and body appreciation) [42,76,77] Positive, intrinsic motivations for going online (eg, information-seeking, enrichment, and learning) [78,79] Adopting strategies to protect privacy and self-regulate use (eg, turning notifications off) [29,80,81] Developing digital literacy and skills to build resilience and promote positive technology use [19,56,73] Learning about self-acceptance, body positivity, and healthy digital practices [42,74] Adopting positive strategies to manage emotions and cope with negative experiences (eg, reporting incidents and seeking help) [58,65]	Existing mental health conditions [20,33,73] Individual temperament—low self-esteem, impulsivity, and fear of missing out [76,82,83] Experience of offline risks or stressors (eg, violence or bullying) [66] Greater digital literacy and skills leading to more screen time and greater exposure to online risks [19,56] Negative or extrinsic motivations (eg, seek approval, avoidance, and to talk to strangers) [48] Remaining passive after an upsetting online experience [65] Maladaptive coping skills (eg, using social media to avoid dealing with negative emotions) [59,84] Gender-related risks (eg, boys and sexual minority youths more likely to engage in risky online behaviors, girls have higher levels of appearance-related social media consciousness and are more likely to engage in comparison, and seek approval from peers) [34,41,45,73-75]	
**Environmental factors**			
	Higher socioeconomic status or family education level [26,71] Social support (online and offline), close family or peer relationships [29,85-87] Parental interest and active mediation of child’s technology use [26,44] Open discussions with family and setting boundaries on technology use together [31,88] Parents model healthy digital habits [86,89]	Lower socioeconomic status or family education level [26,67] Lack of social support, weak family communication [29,87,90] Uncontrolled use of technology, internet access in bedroom [57,91] Strict parental monitoring [78,88] Parental phubbing and negative modeling [26] Restrictions on other activities (eg, COVID-19 restrictions pushed more children online) [57]	

#### Age and Developmental Factors

Most studies included in this review suggested that all children and adolescents are vulnerable to online risks to their health and well-being due to their age. Biological and developmental factors play a significant role in how young people engage with technology and navigate online risks. Younger children, in particular, often struggle to manage their use of digital devices due to their limited cognitive development and digital literacy [37]. Their ability to recognize and respond to online dangers is also less developed compared to older children or adolescents. During adolescence, the brain undergoes crucial developmental changes, particularly in areas responsible for emotion regulation and executive functions [26,52]. This period is characterized by a heightened drive for novelty-seeking, social reward, and a growing sensitivity to external feedback, which can influence online behavior [92,93].

Adolescence is also a time of intense self-discovery and identity formation [42,94]. Young people during this stage are more likely to experience heightened self-consciousness, a stronger need for peer acceptance, and a desire for social belonging [84]. The pursuit of autonomy and independence is central to their development, but so is the increased susceptibility to impulsivity and risk-taking behaviors [95,96]. The adolescent brain’s sensitivity to rewards further contributes to this, making adolescents particularly vulnerable to the allure of online platforms that provide instant gratification and social validation [97]. It is during this period that mental health issues, such as anxiety, depression, and body dissatisfaction, commonly emerge, and young people may struggle with issues of identity and self-worth [74,98].

Some young people appear to be more vulnerable than others due to preexisting vulnerabilities [33,74]. Those with mental health problems, such as low self-esteem or body dissatisfaction, often face increased risks in online environments, as they may be more sensitive to negative feedback or social comparison [99]. Additionally, children who experience vulnerabilities offline—whether due to family circumstances, lack of support, or other factors—are more likely to encounter greater online risks and find it harder to access the help they need [16]. Gender minority youths and those from lower socioeconomic backgrounds may also experience additional stressors that exacerbate mental health challenges, making them more susceptible to the negative effects of online engagement [39,41].

There is mixed evidence regarding gender-related differences in vulnerability to various online risks, but certain trends have emerged. For instance, girls are often subject to greater societal pressures related to physical appearance, which can make them more vulnerable to issues concerning body image and self-esteem [21]. These pressures may be amplified in online spaces, where social comparison is frequent and appearance-related content is pervasive. As a result, the impact of online risks such as cyberbullying or social media comparisons may be more pronounced for some young people, particularly those already dealing with mental health issues or negative self-perceptions [16].

### The Role of Technology Design Features in Mental Health Outcomes

New affordances of social media and smartphones can shape young people’s developmental experiences in several ways. While a limited number of studies explored the impact of specific design features or affordances of social media, the importance of these factors was identified in the literature.

#### Greater Frequency, Immediacy, and Permanence of Communication and Experiences

One of the most notable changes brought about by digital technologies is the frequency, immediacy, and permanence of communication [82,100]. Unlike previous forms of communication, smartphones allow for constant connectivity, facilitating a wider range of simultaneous interactions [101]. With direct messaging and chat functions, young people can engage with both friends and strangers at any time, further amplifying the potential for both positive and negative experiences [31,52,102].

#### Intensified Demands of Peers

24/7 connectivity means that young people are constantly accessible, which can intensify peer demands and expectations, contributing to feelings of stress and pressure. Smartphone notifications and alerts, designed to capture attention, often function as a source of distraction and anxiety [48]. The desire to stay connected is frequently reinforced by features like rewards, streaks, and reminders, which encourage regular communication and content sharing [59,103]. These features can create a cycle of engagement that becomes difficult to disengage from, contributing to feelings of dependency and stress.

#### Altered Nature of Relationships

The nature of online relationships has shifted [104]. Social interactions are increasingly quantified through likes, followers, and comments, which can lead to unhealthy comparisons and a distorted sense of self-worth [21,42,74]. Anonymity online, while allowing for freedom of expression, can also reduce stigma but simultaneously enable harmful behaviors such as cyberbullying [32,42,74].

#### New Opportunities for Connection and Information

Digital platforms offer new opportunities for connection and information-sharing [18]. Young people can access social networks, support groups, and resources related to mental health, which may not be readily available in their offline lives [16]. However, the same platforms that offer valuable support also host inaccurate and harmful content, with hashtags and trends making it easier for users to encounter both positive and detrimental material [32]. The availability of information on mental health, for example, may help users seek support, but it can also expose them to potentially triggering content if not carefully curated [105].

#### Increased Importance of Imagery

The visual nature of many platforms has led to a heightened focus on appearance, perfection, and comparison [41]. Smartphone cameras, photo editing apps, and AI tools enable user-generated content to be created and posted quickly [35]. Features such as filters and image-altering software encourage the creation of idealized versions of self, further contributing to body image concerns and mental health struggles [38,53,74,92,106].

#### Experiences Driven by Data and Business Models

Data and business models play a major role in shaping user experiences, and platforms are competing for users’ attention [97,107]. Algorithms that personalize, curate, and suggest content are central to the design of most social media platforms, and these algorithms can reinforce both positive and negative patterns of behavior [84]. The drive for data monetization through targeted advertising and influencer marketing often blurs the lines between content and advertising, making it difficult for users to discern what is authentic [108]. Algorithmically curated content, particularly content focused on enhancing the users’ appearance and lifestyle, can heighten the pressure young people feel to conform to certain ideals or behaviors, amplifying the impact on their mental health.

### Impact Pathways

Several studies applied theoretical frameworks to try and explain how using digital technologies, particularly social media, may impact young people’s well-being. Five potential impact theories dominated within the studies: (1) transformations and affordances [82,104], (2) socioecological model of development [26,109], (3) uses and gratification theory [63,66,83], (4) development of sociocultural framework [27,34,35,38,92], and (5) time displacement theory [31,34,62,71,83]. Many of these theories consider the complex interactions between the individual characteristics of the user, how technologies are used, and how they are designed (Figure 5).

**Figure 5:**
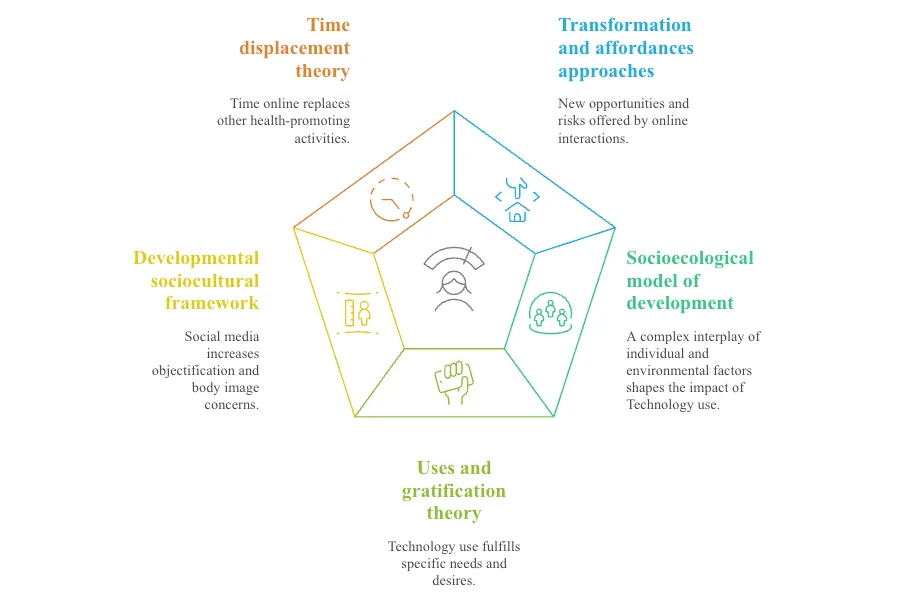
Five theoretical frameworks explored in the literature.

### Recommendations Emerging From Studies

The majority of studies included in this review concluded with recommendations for future research as well as some recommendations for actions to be taken by technology companies, policymakers, caregivers, educators, and clinicians to both protect young people from digital harms and encourage positive uses of technology. The compilation of these recommendations are summarized in Textbox 2.

**Recommended actions extracted from literature to address digital determinants of young people’s mental health and well-being.**

**Priority areas for future research**

- Measure the strength and significance of different mediators and confounding factors that influence the relationship between technology use and mental health outcomes.
- Examine the mental health impacts of different social media features, types of content, and types of online interactions.
- Explore the individual differences of study participants and differences between subgroups to understand motivations for using different platforms and why some young people are more affected by technology uses than others.
- Conduct more longitudinal research to examine the direction of associations and within-person impacts over time as well as to assess whether momentary associations on well-being have long-lasting effects.
- Include younger children in future studies to understand the impacts of early exposure to technology and how mental health outcomes differ according to age.
- Expand the number of experimental designs to test for causal relationships and the effect of different interventions to modify young people’s technology use.
- Reach greater consistency in measures of technology use and mental health outcomes to enable results to be pooled.
- Balance investigation of the impact of social media and digital technology use on both positive and negative mental health and well-being outcomes.

**Actions for technology companies**

- Limit features that encourage compulsive and prolonged use and a focus on physical appearance.
- Enforce age limits on platforms and strengthen efforts to remove harmful content.
- Allow researchers to use data generated by social media companies to enhance understanding of the relationship between technology use and young people’s health and well-being.

**Actions for caregivers, educators, and clinicians**

- Support young people to limit excessive technology use (some researchers propose daily time limits and some reference the World Health Organization’s screen time recommendations).
- Support young people to use alternative emotion regulation strategies and to create healthy habits, for example, around sleep, physical activity, and opportunities for in-person social interaction.
- Build young people’s knowledge and skills on social media literacy, online safety, promoting healthy technology use, and where to get support if they need help.
- Stay informed about the social platforms that young people use and their different benefits and risks.
- Expand school-based, gender-sensitive education on topics such as well-being, building self-esteem, and positive body image.

**Actions for policymakers**

- Strengthen regulation of social media, particularly the highly visual platforms.
- Implement person-centered approaches to minimize the harmful effects of technology use rather than introduce blanket restrictions.
- Invest in mechanisms to build young people’s resilience and make support available for those who experience negative impacts of technology use.

### Limitations of Existing Research

Researchers have identified several limitations in the current evidence base regarding the impact of technology use on young people’s well-being and mental health. One primary concern is the rapid evolution of social media platforms, which causes research to quickly become outdated. As such, more recently popularized platforms like Snapchat and TikTok, as well as the integration of AI within digital platforms, are often underrepresented in studies, creating gaps in our understanding of these social media applications.

Another major limitation is the lack of longitudinal or experimental evidence. The majority of studies are cross-sectional, preventing the establishment of causation or the direction of relationships and failing to capture the transient, momentary effects of technology use. Additionally, there is still a strong focus on screen time as a measure, which does not account for the quality and context of user experiences. The ubiquity and increasingly personalized nature of social media makes it difficult to study the relationship between exposure and outcomes.

A lack of high-quality evidence in this field was a concern for many scholars. Many of the review articles included in this study discussed methodological limitations that they believe diminish the overall quality of the evidence base and may have resulted in conflicting findings on the relationship between young people’s technology use and mental health. Many studies rely on self-reported data about technology use and mental health status, which may be subject to bias or inaccuracy. The diversity in reporting measures and definitions used for well-being or mental health and levels or types of technology use further complicates comparisons across studies. This lack of standardization, coupled with differences in study characteristics such as sample populations, study design, and measurement tools, often limits the generalizability of findings.

Another issue is that the impact of different social media platforms or types of technology use is often grouped together, potentially disguising their distinct effects. Studies tend to focus on a small number of variables, neglecting many possible confounding, mediating, and moderating factors. Few studies consider young people’s personal vulnerabilities, characteristics, and environmental factors, either at baseline or over time. Moreover, other stressors that may have a significant influence on the relationship between digital technology use and well-being and mental health, including those related to the COVID-19 pandemic, are understudied.

Finally, many studies are based on small and nonrepresentative samples, frequently characterized by an overrepresentation of girls among study participants. Considering the differing effects of digital technology use on boys and girls, the generalizability of these findings is constrained. These limitations highlight the need for more comprehensive, longitudinal, and diverse studies to better understand the complex relationship between technology use and young people’s mental health and well-being.

## Discussion

### Principal Findings

This scoping review adds to a growing body of literature exploring the digital determinants of young people’s mental health and well-being. Our analysis of studies published in the past 5 years supports previous interpretations of the complex and bidirectional relationship between young people’s technology use and mental health outcomes. Methodological limitations and insufficient consideration of the multiple factors shaping young people’s digital experiences continue to limit our ability to make robust causal claims about technology use and different types of digital harms. Nevertheless, studies continue to expand our understanding of risks associated with social media and digital technology use and demonstrate that young people who use digital platforms are encountering a range of online risks that pose harm to their mental health and well-being including addictive design, cyberbullying, violence, pornography, pressure to obtain an unrealistic or unhealthy physical appearance or lifestyle, sextortion, self-harm and disordered eating triggers, marketing of harmful products, and gambling.

Certain features of social media and other digital platforms are inherently harmful by design, posing risks to every user’s mental health and well-being. However, the literature indicates that there are a wide range of individual and environmental factors that can increase or decrease the degree of risks that young people may encounter. Furthermore, there are a diverse set of factors that will influence a young person’s resilience or vulnerability to experiencing harm when exposed to these risks. Any policy response to the digital determinants of young people’s mental health and well-being must therefore take such complexities into account. The benefits and risks of digital technology use vary significantly both within and between individuals depending on the specific context in which they occur. Therefore, simplistic, one-size-fits-all governance approaches are unlikely to be effective, particularly for protecting the most vulnerable youths.

The lack of definitive causal evidence on social media use and negative mental health outcomes has led to disagreement within the academic community about the most effective policy responses. Scientific publications claiming that there is no evidence that social media is a driver of worsening mental health and well-being do not resonate with the lived experiences of many young people or their families [110]. At the other end of the spectrum, bold claims about social media being a major driver of poor mental health and well-being have given rise to fears about reactive policy responses that may have harmful unintended consequences for young people. Despite different views, our review identified a number of common recommendations for policymakers and other stakeholders, which aim to minimize harms associated with technology use while also enabling young people to enjoy the benefits offered by social media and digital platforms (Textbox 2).

### Precautionary Policies in Response to Scientific Uncertainty

Lack of scientific consensus has frustrated politicians who are under growing public pressure to protect young people from digital harms and curb the power of technology companies. While policy responses have been slowed by concerns around evidence quality, new laws and guidelines are nonetheless being developed around the world to protect young people and regulate digital platforms. The health sector has historically played a limited role in shaping these governance instruments [111]. However, many health ministries and public health agencies now recognize that young people’s technology use is a public health issue and are pushing for action.

In the face of mixed and inconclusive evidence on the health impacts of technology use, the need for a precautionary governance approach that draws on existing public health knowledge is gaining support. The former US Surgeon General was one of the earliest public health leaders to call for the burden of evidence to be flipped toward proving that social media is safe, rather than proving it is harmful, as is the case in the vast majority of studies that we reviewed [112]. In a report on childhood in the digital age, the Organisation for Economic Co-operation and Development argues that stronger regulation of digital media is warranted even if it is potentially just one of many factors contributing to mental health issues [113]. The Council of the European Union has also concluded that it is prudent for countries to take preventative measures to mitigate the potential risks of digital technologies, even when the scope and consequences of the threat to young people are still partly unknown, given that the associated risks for children’s and adolescents’ mental health may be high [9].

Taking preventative action to protect young people’s health is a common public health approach in situations where there are plausible risks but scientific evidence is not fully established [7]. For example, bans on the use of potentially hazardous chemicals such as bisphenol A and lead in children’s products were enacted in several countries ahead of conclusive evidence of their harmful effects. Restrictions on sales of vapes and vaping in public spaces are also being adopted in some countries ahead of evidence on their long-term health effects.

Based on the findings from this evidence review, and a concurrent analysis of policy interventions across the WHO European Region and globally, 8 recommendations have been proposed to guide future policy actions to promote youth mental health and well-being in a digital age [10]. These recommendations focus on improving young people’s digital well-being by supporting balanced and healthy uses of social media and other digital technologies and making digital environments safer for young people. They recognize that a range of actors from different sectors have a role to play in supporting young people’s mental health and digital well-being. The recommendations also reflect the power of industry and commercial interests in shaping digital environments, thereby calling for stronger regulatory instruments to incentivize young people’s health and safety by design and hold digital technology companies to account.

### Limitations

Our decision to include both evidence reviews and individual primary studies within this scoping review under a single set of eligibility criteria is recognized as a significant methodological limitation. Our approach differs from that expected of a formal umbrella review, which requires distinct research questions and eligibility criteria and a formal assessment of overlap between included reviews. It is therefore possible that some primary studies contributed to our findings both directly, as an included primary study, and indirectly, through their inclusion within one or more of the 63 evidence reviews also included in this review, which may have inflated the apparent volume or consistency of evidence for some findings. We also included a broad range of study types, which vary in their own methodological rigor, and we did not appraise or weigh included reviews by quality or type. We sought to partially mitigate these limitations through transparency: findings from reviews and from primary studies are extracted and reported separately in our supplementary data extraction table, allowing readers to identify which findings derive from which evidence type. Given the rapid time frame and the exploratory, policy-oriented objectives of this review, this inclusive approach reflected a pragmatic trade-off between breadth and methodological precision. We recognize that this limits the extent to which our findings can be treated as a precise quantification of the evidence base. To build on this work with greater methodological rigor, researchers could usefully separate the study types brought together in this review: for example, by undertaking a dedicated umbrella review of the reviews identified here, with formal overlap and quality assessment, alongside a systematic or scoping review of primary studies restricted to those not already synthesized within an included review, or published since the most recent relevant review.

Other methodological restrictions further limited this scoping review. Strict inclusion and exclusion criteria were introduced so that a manageable number of studies were considered for review. This resulted in the omission of literature published in languages other than English and from countries outside the WHO European Region,barring Australia, Canada, and the United States, limiting the generalizability of our findings to the global context. The review only included peer-reviewed publications and omitted other sources of evidence that could provide more timely and granular information on young people’s digital experiences and their effects [114]. Digital trace data, for example, can capture and provide accurate insights into the user’s momentary effects and true nature of use, an ongoing challenge for traditional data collection methods such as self-reported surveys. Internal data and proprietary research held directly by social media companies can provide valuable insights into the effects of particular platform features. Future research should explore the use of such data sources for academic research while following ethical guidelines around user privacy and confidentiality. Finally, the compressed time frame for the review also limited our ability to conduct a full assessment of evidence quality.

### Conclusions

Over 200 articles on the impact of social media and digital technology use on young people’s mental health and well-being were reviewed to inform policy recommendations. Through systematic data extraction and thematic analysis, key findings and limitations from the current evidence base have been summarized and translated into an agenda for action and future research.

Our scoping review also demonstrates that social media and other digital platforms are an important part of many young people’s daily lives and may offer benefits for their health and well-being. Overly restrictive policy responses, for example, blanket bans on smartphones or social media, risk undermining young people’s right to enjoy the positive aspects of these technologies [115]. Policymakers across the WHO European Region and beyond are committed to protecting and promoting the health of young people in digital environments. A pressing challenge is to design and implement regulation that is nuanced, taking into consideration children’s age, developmental stage, and other vulnerabilities, and can balance the positive and negative digital determinants of young people’s health and well-being. Co-designing policies with young people and involving them in ongoing research and accountability efforts are critical to ensure that policy interventions support their rights and respond to their diverse needs and experiences.

As digital technologies continue to develop at a rapid pace, research and policy must proactively monitor and adapt to the changing landscape. Many of the studies included in this review call for continued and longitudinal research to understand the dynamic relationship between technology use and young people’s well-being. In particular, further studies are needed to understand why some young people are more vulnerable than others to negative impacts of digital technology use and to explore which aspects of digital design and use characteristics most significantly impact young people’s mental health and well-being. Academics have called for new approaches to research on digital technologies and their potential harms in situations where innovation and reports of negative effects outpace the ability of scientists to assess them [116]. Such an approach would empower policymakers to take more timely action in the future. However, while further evidence on the digital determinants of young people’s mental health continues to emerge, it is clear that there is sufficient evidence to act now.

## Appendix

Multimedia Appendix 1 Research questions and search strategy.

Multimedia Appendix 2 PRISMA ScR Checklist.

Multimedia Appendix 3 Data extraction tables.

## Abbreviations

PRISMA: Preferred Reporting Items for Systematic Reviews and Meta-Analyses
PRISMA-ScR: Preferred Reporting Items for Systematic Reviews and Meta-Analyses Extension for Scoping Reviews

## Data Availability

The summarized data tables are supplied in supporting files available for download along with the published manuscript. The full datasets generated and analyzed during this study are available from the corresponding author on reasonable request.

## Generative AI Use

AI tools were used to support data processing and visualization. Gemini (Google) was used to assist with checking, cleaning, and recoding data in the data extraction sheet. The data extraction framework and initial data entry were completed manually by the research team. Claude (Anthropic) was used to generate visualizations based on the extracted data. The authors reviewed and verified all AI-assisted data processing and visual outputs and remain fully responsible for the accuracy, interpretation, and integrity of the data and analyses presented in this manuscript.
