# Supplementary material for: Informing Policy Responses to Digital Technology Use and Youth Mental Health and Well-Being: Scoping Review
Source: Online J Public Health Inform. 2026 Oct 21;18:e90160. doi: 10.2196/90160 (PMC13735201; doi:10.2196/90160)
Supplement: Supplementary file 1 [file ojphi_v18i1e90160_app1.docx]

1. **Research questions to guide the evidence review**
RQ1. What research has been conducted about the impact of social media and digital technology use on young people’s mental health and well-being?
     1a. What kinds of studies have been published?
     1b. What types of social media and digital technology use are studied?
     1c. What types of mental health or well-being outcomes are studied?
     1d. Which mediating factors were studied?
     1e. What are the characteristics (age, gender, location etc) of the subjects of the research?
RQ2. What are the key findings on the relationship between young people’s use of social media and digital technologies and mental health / well-being outcomes?
2a. What types of (positive and negative) mental health and well-being impacts are reported in the literature?
2b. How do results differ by age group and gender?
2c. What factors (individual and environmental) make young people more vulnerable or resilient to negative mental health and well-being impacts?
2d. What factors (individual and environmental) help young people to use social media and digital technology in ways that enhance their mental health and well-being?
2e. What factors (individual and environmental) make young people use social media and digital technology in ways that are harmful to their mental health and well-being?
2f. What are the potential impact pathways proposed in the literature?
2g. What features of social media and other digital technologies are contributing to positive or negative mental health or well-being outcomes?

RQ3. What recommendations have researchers made for policy action or further research?

RQ4. What are the limitations of the current evidence base?

**2. Search terms used to structure the literature search**

| **Group 1 - population terms** | **Group 2 - technology terms** | **Group 3 - outcome terms** |
| --- | --- | --- |
| child OR children OR teen OR adolescent OR girl OR boy OR youth OR young people | digital OR technology OR social media OR social network OR game OR gaming OR smartphone OR online OR media OR virtual OR screen OR mobile OR internet OR TikTok OR Instagram OR Snapchat OR Facebook OR YouTube | mental OR health OR well-being OR wellbeing OR anxiety OR depression OR addiction OR bullying OR suicide OR loneliness OR social isolation OR eating disorder OR mood OR cognitive development OR emotion OR harm OR risk OR stress OR resilience OR self-esteem OR social connection OR motivation OR life satisfaction |

**3. Search strings used for database searches on 29 November 2024**

a) **PubMed**

( "Adolescent"[MeSH Terms] OR "Child"[MeSH Terms] OR "Youth"[MeSH Terms] OR "Minors"[MeSH Terms] OR child[tiab] OR children[tiab] OR teen[tiab] OR teenager[tiab] OR teenagers[tiab] OR adolescent[tiab] OR adolescents[tiab] OR adolescence[tiab] OR girl[tiab] OR girls[tiab] OR boy[tiab] OR boys[tiab] OR youth[tiab] OR "young people"[tiab] OR "young person"[tiab] OR "young adults"[tiab] OR "school-aged"[tiab] OR "school aged"[tiab] OR pediatric[tiab] OR paediatric[tiab] ) AND ( "Social Media"[MeSH Terms] OR "Internet"[MeSH Terms] OR "Computers, Handheld"[MeSH Terms] OR "Smartphone"[MeSH Terms] OR "Video Games"[MeSH Terms] OR "Screen Time"[MeSH Terms] OR "social media"[tiab] OR "social network"[tiab] OR "social networking"[tiab] OR TikTok[tiab] OR Instagram[tiab] OR Snapchat[tiab] OR Facebook[tiab] OR YouTube[tiab] OR "online platform"[tiab] OR "digital platform"[tiab] OR smartphone[tiab] OR smartphone[tiab] OR "mobile phone"[tiab] OR "mobile device"[tiab] OR internet[tiab] OR online[tiab] OR gaming[tiab] OR "video game"[tiab] OR "video games"[tiab] OR "screen time"[tiab] OR screentime[tiab] OR "digital technology"[tiab] OR "digital media"[tiab] OR "digital device"[tiab] OR virtual[tiab] OR "digital use"[tiab] OR "technology use"[tiab] ) AND ( "Mental Health"[MeSH Terms] OR "Well-Being"[MeSH Terms] OR "Depression"[MeSH Terms] OR "Anxiety"[MeSH Terms] OR "Suicide"[MeSH Terms] OR "Self-Injurious Behavior"[MeSH Terms] OR "Bullying"[MeSH Terms] OR "Cyberbullying"[MeSH Terms] OR "Loneliness"[MeSH Terms] OR "Feeding and Eating Disorders"[MeSH Terms] OR "Self Concept"[MeSH Terms] OR "Stress, Psychological"[MeSH Terms] OR "Cognitive Development"[MeSH Terms] OR "Resilience, Psychological"[MeSH Terms] OR "Behavior, Addictive"[MeSH Terms] OR "mental health"[tiab] OR wellbeing[tiab] OR "well-being"[tiab] OR anxiety[tiab] OR depression[tiab] OR depressive[tiab] OR addiction[tiab] OR addictive[tiab] OR "problematic use"[tiab] OR bullying[tiab] OR cyberbullying[tiab] OR suicide[tiab] OR suicidal[tiab] OR "self-harm"[tiab] OR "self harm"[tiab] OR loneliness[tiab] OR "social isolation"[tiab] OR "eating disorder"[tiab] OR "eating disorders"[tiab] OR mood[tiab] OR "cognitive development"[tiab] OR emotion[tiab] OR harm[tiab] OR risk[tiab] OR stress[tiab] OR resilience[tiab] OR "self-esteem"[tiab] OR "self esteem"[tiab] OR "social connection"[tiab] OR "life satisfaction"[tiab] OR motivation[tiab] OR "mental ill-health"[tiab] ) AND ( "2020/01/01"[Date - Publication] : "2024/11/29"[Date - Publication] ) AND ( "humans"[MeSH Terms] )

b) **PsychNET / PsycINFO**

( DE "Adolescents" OR DE "Children" OR DE "Youth" OR DE "College Students" OR DE "Early Adolescence" OR DE "Late Adolescence" OR DE "Preadolescents" OR DE "Elementary School Students" OR DE "High School Students" OR AB(child OR children OR teen OR teenager OR adolescent OR adolescents OR adolescence OR girl OR girls OR boy OR boys OR youth OR "young people" OR "young person" OR "young adults" OR "school-aged" OR pediatric OR paediatric) ) AND ( DE "Social Media" OR DE "Social Networking (Internet)" OR DE "Internet" OR DE "Smartphones" OR DE "Computer Games" OR DE "Video Games" OR DE "Screen Time" OR DE "Mobile Phones" OR DE "Online Socialization" OR AB("social media" OR "social network" OR "social networking" OR TikTok OR Instagram OR Snapchat OR Facebook OR YouTube OR smartphone OR "mobile phone" OR "mobile device" OR internet OR online OR gaming OR "video game" OR "video games" OR "screen time" OR screentime OR "digital technology" OR "digital media" OR "digital platform" OR "digital device" OR virtual OR "technology use" OR "digital use") ) AND ( DE "Mental Health" OR DE "Psychological Wellbeing" OR DE "Depression (Emotion)" OR DE "Major Depression" OR DE "Anxiety" OR DE "Anxiety Disorders" OR DE "Suicidal Ideation" OR DE "Suicide" OR DE "Self-Injurious Behavior" OR DE "Bullying" OR DE "Cyberbullying" OR DE "Loneliness" OR DE "Social Isolation" OR DE "Eating Disorders" OR DE "Self-Esteem" OR DE "Stress" OR DE "Cognitive Development" OR DE "Resilience (Psychological)" OR DE "Addictive Behavior" OR DE "Internet Addiction" OR DE "Life Satisfaction" OR AB("mental health" OR wellbeing OR "well-being" OR anxiety OR depression OR depressive OR addiction OR addictive OR "problematic use" OR bullying OR cyberbullying OR suicide OR suicidal OR "self-harm" OR "self harm" OR loneliness OR "social isolation" OR "eating disorder" OR "eating disorders" OR mood OR "cognitive development" OR emotion OR harm OR risk OR stress OR resilience OR "self-esteem" OR "self esteem" OR "social connection" OR "life satisfaction" OR motivation OR "mental ill-health") )

Date range 1 January 2020-29 November 2024 applied as a filter.

c) **Google Scholar**

Search 1:

("social media" OR "social networking" OR TikTok OR Instagram OR Snapchat OR Facebook OR Instagram) ("mental health" OR "well-being" OR depression OR anxiety OR "self-esteem") (adolescent OR teenager OR youth OR "young people" OR children)

Search 2:

("screen time" OR "smartphone use" OR "mobile phone use" OR "internet use") ("mental health" OR wellbeing OR depression OR anxiety OR loneliness OR stress) (adolescent OR teenager OR "young people" OR children)

Search 3:

(gaming OR "video games" OR "online gaming" OR "digital technology") ("mental health" OR wellbeing OR "cognitive development" OR addiction OR aggression) (adolescent OR teenager OR youth OR children)

Date range 1 January 2020-29 November 2024 applied as a filter.
