# Supplementary material for: Informing Policy Responses to Digital Technology Use and Youth Mental Health and Well-Being: Scoping Review
Source: Online J Public Health Inform. 2026 Oct 21;18:e90160. doi: 10.2196/90160 (PMC13735201; doi:10.2196/90160)
Supplement: Supplementary file 2 [file ojphi_v18i1e90160_app2.docx]

**Preferred Reporting Items for Systematic reviews and Meta-Analyses extension for Scoping Reviews (PRISMA-ScR) Checklist**

**Manuscript title:** Informing policy responses to digital technology use and youth mental health and well-being: Findings from a rapid scoping review

**Corresponding author:** Louise Holly [louise.holly@dthlab.org](mailto:louise.holly@dthlab.org)

Page numbers refer to the clean version of the revised manuscript submitted alongside this checklist.

| **SECTION** | **ITEM** | **PRISMA-ScR CHECKLIST ITEM** | **REPORTED ON PAGE #** |
| --- | --- | --- | --- |
| **TITLE** | | | |
| Title | 1 | Identify the report as a scoping review. | Page 1 – Title reads “Informing policy responses to digital technology use and youth mental health and well-being: Findings from a rapid scoping review” |
| **ABSTRACT** | | | |
| Structured summary | 2 | Provide a structured summary that includes (as applicable): background, objectives, eligibility criteria, sources of evidence, charting methods, results, and conclusions that relate to the review questions and objectives. | Pages 1-2 – Structured abstract includes background, objectives, methods, results and conclusions |
| **INTRODUCTION** | | | |
| Rationale | 3 | Describe the rationale for the review in the context of what is already known. Explain why the review questions/objectives lend themselves to a scoping review approach. | Pages 2-4 – The introduction summarizes existing literature, describes the context in which this scoping review was conducted and the rationale for a rapid scoping review. |
| Objectives | 4 | Provide an explicit statement of the questions and objectives being addressed with reference to their key elements (e.g., population or participants, concepts, and context) or other relevant key elements used to conceptualize the review questions and/or objectives. | Page 4 – The primary objective of the study is stated at the end of the introduction section ‘Objectives’ and details the research questions used to structure the data analysis. |
| **METHODS** | | | |
| Protocol and registration | 5 | Indicate whether a review protocol exists; state if and where it can be accessed (e.g., a Web address); and if available, provide registration information, including the registration number. | The protocol was not registered a priori in a public registry. |
| Eligibility criteria | 6 | Specify characteristics of the sources of evidence used as eligibility criteria (e.g., years considered, language, and publication status), and provide a rationale. | Pages 6-7 – The eligibility criteria for included studies are described in the methods section ‘Eligibility criteria’. These were categorized according to: study population; type of digital technology use; type of mental health and well-being outcomes; duration of study; geographical scope; study types; language. |
| Information sources* | 7 | Describe all information sources in the search (e.g., databases with dates of coverage and contact with authors to identify additional sources), as well as the date the most recent search was executed. | Page 7 – The search strategy, including databases searched, are detailed in the methods section ‘Search strategy and selection criteria’. |
| Search | 8 | Present the full electronic search strategy for at least 1 database, including any limits used, such that it could be repeated. | A full list of search terms and search strings used are provided in Multimedia Appendix 2. |
| Selection of sources of evidence† | 9 | State the process for selecting sources of evidence (i.e., screening and eligibility) included in the scoping review. | Page 6 – The methods section ‘Search strategy and selection criteria’ details the process undertaken to screen literature and determine eligibility criteria. |
| Data charting process‡ | 10 | Describe the methods of charting data from the included sources of evidence (e.g., calibrated forms or forms that have been tested by the team before their use, and whether data charting was done independently or in duplicate) and any processes for obtaining and confirming data from investigators. | Pages 6-7 – The methods section ‘Data extraction and analysis’ describes how data was organized and analyzed using a thematic analysis approach. |
| Data items | 11 | List and define all variables for which data were sought and any assumptions and simplifications made. | Page 6 – We extracted the following data on study characteristics and content based on the research questions: (1) study characteristics (author, year, country, study design, sample size, age group); (2) digital technology types and behaviors examined; (3) mental health and well-being outcomes examined; (4) key findings on the relationship between technology use and mental health outcomes, including direction and strength of association where reported; (5) moderating and mediating factors identified; (6) researcher recommendations for policy action or future research; and (7) methodological limitations identified by study authors. |
| Critical appraisal of individual sources of evidence§ | 12 | If done, provide a rationale for conducting a critical appraisal of included sources of evidence; describe the methods used and how this information was used in any data synthesis (if appropriate). | A formal assessment of the quality of individual studies was not conducted which is consistent with scoping review methodology. This is noted in the limitations section on page 27. |
| Synthesis of results | 13 | Describe the methods of handling and summarizing the data that were charted. | Pages 6-7 – The methods section ‘Data extraction and analysis’ describes how data was extracted first into an Excel spreadsheet for organizing according to research questions and then relevant data was transferred into a Word document for synthesis and further thematic analysis. |
| **RESULTS** | | | |
| Selection of sources of evidence | 14 | Give numbers of sources of evidence screened, assessed for eligibility, and included in the review, with reasons for exclusions at each stage, ideally using a flow diagram. | Figure 1 (page 8) shows a PRISMA flow diagram. Our initial literature search yielded 7,420 articles. Following title and abstract screening and de-duplication, 419 records were identified for full text screening. Full-text screening resulted in a total of 226 articles meeting the inclusion criteria and being selected for data extraction and analysis. |
| Characteristics of sources of evidence | 15 | For each source of evidence, present characteristics for which data were charted and provide the citations. | Figure 3 (page 9) describes the design characteristics and frequency of included studies.  Table 1 (pages 10-11) describes the categories of digital technology and social media use examined in included studies.  Table 2 (pages 11-12) describes the mental health, well-being outcomes and intermediate factors examined in included studies.  Table 3 (pages 16-17) Individual and environmental factors associated with increased resilience (protective factors) or vulnerability (risk factors) to the mental health impacts of digital technology use among children, adolescents and youth  Box 1 (page 22) summarizes recommendations extracted from included studies. |
| Critical appraisal within sources of evidence | 16 | If done, present data on critical appraisal of included sources of evidence (see item 12). | Not applicable — no critical appraisal  performed (see item 12). |
| Results of individual sources of evidence | 17 | For each included source of evidence, present the relevant data that were charted that relate to the review questions and objectives. | In addition to Tables 1–3 (see item 15) a narrative synthesis of results is provided in the Results section (pages 7-25) |
| Synthesis of results | 18 | Summarize and/or present the charting results as they relate to the review questions and objectives. | The results section (pages 7-25) presents the findings of the rapid scoping review. Findings are structured into sub-sections which correspond to the different research questions and emergent themes. |
| **DISCUSSION** | | | |
| Summary of evidence | 19 | Summarize the main results (including an overview of concepts, themes, and types of evidence available), link to the review questions and objectives, and consider the relevance to key groups. | The first section of the discussion section summarizes the main results of the study (pages 25-26). The subsequent section (pages 26-27 interprets and analyzes the findings in relation to current policy debates and WHO’s request from member states to develop recommendations for action. |
| Limitations | 20 | Discuss the limitations of the scoping review process. | Limitations of the study are described at the end of the discussion (pages 27-28). |
| Conclusions | 21 | Provide a general interpretation of the results with respect to the review questions and objectives, as well as potential implications and/or next steps. | The conclusions section (page 28-29) summarizes the contribution of the study with some recommendations for future research and policy. |
| **FUNDING** | | | |
| Funding | 22 | Describe sources of funding for the included sources of evidence, as well as sources of funding for the scoping review. Describe the role of the funders of the scoping review. | Sources of funding and the role of funders are described on page 31. |

JBI = Joanna Briggs Institute; PRISMA-ScR = Preferred Reporting Items for Systematic reviews and Meta-Analyses extension for Scoping Reviews.

* Where *sources of evidence* (see second footnote) are compiled from, such as bibliographic databases, social media platforms, and Web sites.

† A more inclusive/heterogeneous term used to account for the different types of evidence or data sources (e.g., quantitative and/or qualitative research, expert opinion, and policy documents) that may be eligible in a scoping review as opposed to only studies. This is not to be confused with *information sources* (see first footnote).

‡ The frameworks by Arksey and O’Malley (6) and Levac and colleagues (7) and the JBI guidance (4, 5) refer to the process of data extraction in a scoping review as data charting*.*

§ The process of systematically examining research evidence to assess its validity, results, and relevance before using it to inform a decision. This term is used for items 12 and 19 instead of "risk of bias" (which is more applicable to systematic reviews of interventions) to include and acknowledge the various sources of evidence that may be used in a scoping review (e.g., quantitative and/or qualitative research, expert opinion, and policy document).

*From:* Tricco AC, Lillie E, Zarin W, O'Brien KK, Colquhoun H, Levac D, et al. PRISMA Extension for Scoping Reviews (PRISMAScR): Checklist and Explanation. Ann Intern Med. 2018;169:467–473. [doi: 10.7326/M18-0850](http://annals.org/aim/fullarticle/2700389/prisma-extension-scoping-reviews-prisma-scr-checklist-explanation).
