# Supplementary material for: Informing Policy Responses to Digital Technology Use and Youth Mental Health and Well-Being: Scoping Review
Source: Online J Public Health Inform. 2026 Oct 21;18:e90160. doi: 10.2196/90160 (PMC13735201; doi:10.2196/90160)
Supplement: Supplementary file 3 [file ojphi_v18i1e90160_app3.docx]

**Table S1. Summary of included studies (reviews)**

| **Study ID** | **Author name (year)** | **Study design** | **Exposures studied** | **Outcomes studied** | **Types of online content, behaviours or experiences studied** | **Key findings** | **Direction of evidence** |
| --- | --- | --- | --- | --- | --- | --- | --- |
| [1](https://www.zotero.org/google-docs/?INbvNM) | Arias-de la Torre et al (2020) | Umbrella review | Mobile technologies and social media, instant messaging, social networking sites, social comparison, user engagement quality and quantity, problematic use or addiction | Depression, anxiety, well-being | Problematic and general use of social networking sites, time spent, quantity and quality of user engagement | Excessive social comparison and personal involvement when using mobile technologies and social media (MTSM) could be associated with the development of depressive symptomatology. Adaptive use of MTSM could help prevent the development of depression, promote social support, and become a point of information access. | Mixed |
| [2](https://www.zotero.org/google-docs/?1muo5a) | Dienlin & Johannes (2020) | Narrative review | Social networking sites (Instagram, TikTok), instant messengers (WhatsApp, Signal) | Eudaimonic well-being (life satisfaction), hedonic well-being (positive and negative affect) | Passive and active use, social or non-social use, procrastination vs. goal-directed behaviors | The general effects of digital technology use on well-being are likely in the negative spectrum, but very small. No screen time is created equal; different uses lead to different effects. Low and excessive use are related to decreased well-being, whereas moderate use is related to increased well-being. | Mixed |
| [3](https://www.zotero.org/google-docs/?XtbafI) | Ghaemi (2020) | Narrative review | Frequency of social media use | Digital depression, cyberbullying, sexual harassment, increased drug use | Sexting, viewing porn, sharing sexualized photos | Psychiatric outcomes have worsened in adolescents in the past decade, correlating with the invention of the smartphone and the rise of social media. Depressive symptoms and suicide rates have risen. | Negative |
| [4](https://www.zotero.org/google-docs/?eiHgy6) | Ivie et al (2020) | Meta-analysis | Amount of time spent on social media | Depression | General social media use | A small but positive association between social media use and depressive symptoms. High heterogeneity suggests substantial differences between studies. | Mixed |
| [5](https://www.zotero.org/google-docs/?Ve3FYo) | Lopez-Fernandez & Kuss (2020) | Other review type | Problematic internet use, general internet addiction, specific internet addictions | Mental health disorders | Gaming addiction, internet addiction, gambling addiction | Generalised Internet Addiction is associated with anxiety and mood disorders. Internet gaming addiction has positive results with CBT. Internet Gambling disorder is associated with depression, anxiety, and social phobia. | Negative |
| [6](https://www.zotero.org/google-docs/?YhX6bL) | McCrory, Best & Maddock (2020) | Scoping review | Visual social media (Facebook, Instagram, Snapchat) | Mental health, well-being, loneliness, depression, self-esteem, body perception | Time spent on social media, online self-presentation (selfies), use of filters | Young people who spend more time on social media sites were found to be more relaxed and felt a greater sense of social connection. Other studies found that the more time spent using social media sites, the greater the levels of loneliness and depression. Act of taking selfies: Taking and posting selfies was found to increase self-esteem, body satisfaction and self-confidence, however, it was also found to increase anxiety, body consciousness and decrease confidence and self perception. Photo manipulation and digital filters were found to have negative connections with body perception/body dissatisfaction. | Mixed |
| [7](https://www.zotero.org/google-docs/?gZ89Wg) | Odgers & Jensen (2020a) | Narrative review | Overall digital technology use | Overall well-being, mental health, depression | All types of online content, behaviors, or experiences | There is little evidence to support the claims that time spent online is associated with increased risk for mental-health problems. Heavier texters over the study period actually reported less depression than teens who texted less frequently. Offline vulnerabilities tend to mirror online vulnerabilities. | Mixed |
| [8](https://www.zotero.org/google-docs/?XLcCrV) | Odgers & Jensen (2020b) | Narrative review | Online communication, social media, social networking sites | Mental health, well-being, depression, anxiety, self-esteem, loneliness | Daily social media use | Most research to date has been correlational, focused on adults versus adolescents, and has generated a mix of often conflicting small positive, negative and null associations. The most recent and rigorous large-scale preregistered studies report small associations between the amount of daily digital technology usage and adolescents' well-being that do not offer a way of distinguishing cause from effect and are unlikely to be of clinical or practical significance. | Mixed |
| [9](https://www.zotero.org/google-docs/?DOSQhG) | Piteo & Ward (2020) | Systematic review | Social networking site usage | Depression and/or anxiety symptoms | Amount of time spent, frequency of visits, investment in social networking sites, active vs. passive use | Increased time spent or frequency of social network site (SNS) use, and problematic and addictive behaviour on SNS were significantly associated with higher levels of depressive symptoms. Increased time spent or frequency of SNS use and higher levels of investment on SNS were significantly associated with higher levels of anxiety symptoms. However, other potential confounding factors could explain the relationship between SNS and depressive and anxiety symptoms, including perceived social support, social comparison and fear of missing out. | Mixed |
| [10](https://www.zotero.org/google-docs/?TVXhgI) | Pottie et al (2020) | Systematic review | Social media and Information and Communication Technologies (ICTs) | Health, well-being, inclusion, and integration | Social networking, presentation of self, health information consumption, and political rights | Refugee youth commonly use ICTs for social networking, presentation of self, health information consumption, and political rights. Uptake of social media through personal mobile devices, such as smartphones, positively enhanced interactions of newly arrived refugees. | Positive |
| [11](https://www.zotero.org/google-docs/?FrI87I) | Rounsefell et al (2020) | Systematic review | Habitual social media use | Body perception (satisfaction or dissatisfaction) and food choices | Neutral engagement or maladaptive engagement, Idyllic Body pictures (fitspiration, selfies, etc.) | Social media engagement or exposure to picture-related content was associated with higher body dissatisfaction, dieting/restricting food, overeating, and choosing healthy foods. Qualitative analysis identified five themes: (i) social media encourages comparison between users, (ii) comparisons heighten feelings about the body, (iii) young adults modify their appearance to portray a perceived ideal representation, (iv) young adults are aware of social media's impact on body perception and food choices, however, (v) external validation via social media is pursued. | Negative |
| [12](https://www.zotero.org/google-docs/?6ON64c) | Schønning et al (2020) | Scoping review | Social media and social networking sites, online communication | Mental health and well-being (depression, anxiety, psychiatric problems, body dissatisfaction, sleep, self-harm, loneliness, self-esteem, FOMO) | Frequency and duration of use, social media addiction, specific actions (liking, commenting), peer comparison, cyberbullying | About 3/4 of the included studies focused on social media and some aspect of pathology. Focus on the potential association between social media use and positive outcomes seems to be rarer. The majority of the included studies tend to find a correlation between more frequent social media use and poor well-being and/or mental health. Few separated between different forms of (inter)actions on social media, which are likely to be differentially associated with mental health and well-being outcomes. | Negative |
| [13](https://www.zotero.org/google-docs/?jW4XHL) | Vidal et al (2020) | Scoping review | Internet use, Social networking sites, Mobile use | Depression and suicidality | Quantity of SNS use, quality of SM use, social aspects, disclosure of mental health symptoms | The studies reviewed yielded four main themes in social media (SM) use: quantity of SM use, quality of SM use, social aspects associated with SM use, and disclosure of mental health symptoms. Most articles demonstrated a positive and bi-directional association between frequency of SM use and depression and in some instances even suicidality. There are possible differential effects between passive and active, and private versus public SM use. | Mixed |
| [14](https://www.zotero.org/google-docs/?dv5CsW) | Alonzo et al (2021) | Systematic review | Social media use | Anxiety, depression, psychological distress, sleep quality | Active social media use | Among cross-sectional studies, significant associations between excessive social media use with poor mental health outcomes, poor sleep quality, and significant associations between poor sleep quality and negative mental health were found. In longitudinal studies, frequent social media use was a risk factor for both poor mental health and poor sleep outcomes. Some studies showed sleep quality mediating the relationship between social media use and negative mental health outcomes. | Negative |
| [15](https://www.zotero.org/google-docs/?zX1O5w) | Course-Choi & Hammond (2021) | Narrative review | Type of social media use | Well-being | Frequent social media use, active vs. passive use, disordered use | Most research distinguishing type of social media use, as opposed to mere frequency of use, is producing more significant results. Passive use (i.e., browsing) as opposed to active use (i.e., liking/posting/commenting) is linked to greater depressed mood. Disordered (as opposed to heavy) use is implicated in lower levels of life satisfaction, while compulsive Facebook use affects perceived academic performance. | Mixed |
| [16](https://www.zotero.org/google-docs/?6J0hy7) | Ehrenreich et al (2021) | Narrative review | Smartphones, digital communication platforms (social media) | Mental health, adolescent development | Texting, messaging, cyberbullying, sexting | Digital communication provides forums for adolescents to navigate intimate peer relationships, establish autonomy, and identity exploration. Associations with mental health are mixed. Passive use is associated with poorer adjustment. Cyberbullying and sexting are common. | Mixed |
| [17](https://www.zotero.org/google-docs/?iKYolz) | Loleska & Pop-Jordanova (2021) | Narrative review | Problematic smartphone use, addiction | Addiction (compulsive gambling, smartphone use) | Not specified | Problematic smartphone use is characterized by compulsive use resulting in physical, psychological, or social harm. Strongly associated with depression, anxiety, sleep deficit, and stress. | Negative |
| [18](https://www.zotero.org/google-docs/?3bChWJ) | Stoilova, Livingstone & Khazbak (2021) | Other review type | Internet use, digital technologies, online experiences | Well-being, mental health, resilience | Sexual experiences, online risks, digital privacy, online information, mental health support | Internet use can contribute positively to well-being, but also amplifies risk of harm (cyberbullying, sexual exploitation, etc.). What children do online is more important than time spent. Vulnerable children offline are more vulnerable online. | Mixed |
| [19](https://www.zotero.org/google-docs/?41IJIJ) | Tang et al (2021) | Systematic review | Screen time | Internalizing mental health symptoms (depression, anxiety, self-esteem, psychological well-being) | Total screen time, TV/video watching, computer/internet use, mobile phone use, social media use, videogame use | The association between screen time and subsequent depressive symptoms is small to very small. Limited evidence of reverse association. Little evidence supporting longitudinal associations with anxiety, self-esteem, and general internalizing problems. | Mixed |
| [20](https://www.zotero.org/google-docs/?FjDb8n) | Bozzola et al (2022) | Scoping review | Social media use | Mental health risks (depression, psychological problems) | Not specified | Social media use is associated with risks like depression, diet and weight problems, cyberbullying, psychological problems, sleep issues, addiction, anxiety, sex-related issues, behavioral problems, body image, reduced physical activity. | Negative |
| [21](https://www.zotero.org/google-docs/?RyM7sk) | Choukas-Bradley et al (2022) | Other review type | Social media (highly visual platforms: Instagram, Snapchat, TikTok) | Body image, depressive symptoms, disordered eating | Exposure to idealized images, social comparison, idealized self-presentation, feedback seeking | Social media features (idealized images, quantifiable feedback) intersect with adolescent development and sociocultural gender socialization to exacerbate girls' body image concerns, which may underlie associations between SM use and mental health (depressive symptoms, disordered eating). | Negative |
| [22](https://www.zotero.org/google-docs/?1taa9x) | Ferguson et al (2022) | Meta-analysis | Screen media (social media, smartphones) | Mental health | Not specified | Screen media, including smartphones and social media, play little to no role in mental health concerns or suicidal ideation. Concerns about screen time may distract from more pressing causes of mental health decline. | Mixed |
| [23](https://www.zotero.org/google-docs/?qM46wC) | Haddock et al (2022) | Scoping review | Digital technology use | Brain, cognitive, and social-emotional development | Internet use, social media, video games | Digital technology use has specific positive effects on adolescent brain, cognitive, and social-emotional development, as well as mental health and well-being, including improvements in executive control, motivation, and social connection. | Positive |
| [24](https://www.zotero.org/google-docs/?HTdkTt) | Kostyrka-Allchorne et al (2022) | Systematic review | Digital experiences, social media use | Anxiety, depression, eating disorders, nonsuicidal self-injury | Viewing harmful content, cyberbullying, seeking information/help for mental health, attention-seeking behaviors (posting self-harm or sexually suggestive photos) | Digital practices of adolescents with pre-existing clinical vulnerabilities encompass both positive and negative experiences. The current literature is too limited to determine if their digital experiences differ significantly from those without mental health disorders. | Mixed |
| [25](https://www.zotero.org/google-docs/?IIjvMk) | Liu et al (2022) | Mixed review | Social media use | Depression | Not discussed | Time spent on social media is significantly associated with depression in a linear, dose-dependent manner, with this correlation being more prominent in girls. | Negative |
| [26](https://www.zotero.org/google-docs/?RXMQEM) | Ricci et al (2022) | Systematic review | Information and communication technologies, internet use, digital technology use | Psychosocial development, well-being, mental health | Appearance-focused games, social media, excessive internet use, advertising of unhealthy foods | Excessive technology use is associated with intellectual deficits and mental health issues, but it can also enable psychosocial development, social connection, and the strengthening of friendships. | Mixed |
| [27](https://www.zotero.org/google-docs/?aFIUzP) | Shannon et al (2022) | Mixed review | Problematic social media use | Mental health (depression, anxiety, stress) | Problematic, excessive, or compulsive use | Problematic social media use has moderate but statistically significant correlations with depression, anxiety, and stress. | Negative |
| [28](https://www.zotero.org/google-docs/?rflpbc) | Valkenburg, Meier & Beyens (2022) | Umbrella review | Active/passive, private/public usage of social media platforms (Instagram, Snapchat, Facebook, WeChat, WhatsApp) | Mental health, well-being | Not discussed | Associations between social media use and mental health are generally weak or inconsistent, with social network site use being weakly associated with both higher levels of ill-being and well-being. | Mixed |
| [29](https://www.zotero.org/google-docs/?4fVU6O) | Vissenberg, d'Haenens & Livingstone (2022) | Systematic review | Negative online experiences | Well-being | Online risk experiences (cyberbullying, exposure to violent content) | Negative online risk experiences are associated with lower well-being, though negative emotions are often not long-lasting. Digital literacy and online resilience can function as promotive and protective factors for well-being. | Negative |
| [30](https://www.zotero.org/google-docs/?XLi1pz) | Blanchard et al (2023) | Systematic review | Social media use | Mental health outcomes (body image, self-esteem, stress, depressive symptoms), dietary outcomes (disordered eating, obesity) | Social media influencers, photo-related activities, appearance-based social comparison | Social media use is significantly correlated with depressive symptoms, disordered eating symptoms, body dissatisfaction, and anxiety. | Negative |
| [31](https://www.zotero.org/google-docs/?1NjPyn) | Chochol, Gandhi & Croarkin (2023) | Narrative review | Social media (user-generated content, user-specific profiles) | Anxiety | Visual content, likes, messaging, different platforms | Associations between the quantity of digital technology use and mental health outcomes are inconsistent and small. Positive well-being effects are often related to social support derived from self-disclosure on social media. | Mixed |
| [32](https://www.zotero.org/google-docs/?NDghSX) | Choukas-Bradley et al (2023) | Narrative review | Social media use | Mental health (body image concerns, disordered eating, depression, suicidality) | Selfie posting, photo-editing, body positivity content | Social media acts as a double-edged sword, offering both opportunities for community and support, and risks such as cyberbullying and exposure to discrimination, which can lead to depressive symptoms and suicidality. | Mixed |
| [33](https://www.zotero.org/google-docs/?Hj8pKs) | Dane & Bhatia (2023) | Scoping review | Social media use (content creation, social networking, virtual communities) | Body image, eating disorders | Social media trends (#thinspiration, pro-eating disorder content), appearance-focused platforms, investment in photos | Social media usage leads to body image concerns, disordered eating, and poor mental health, mediated by social comparison, thin/fit ideal internalization, and self-objectification. | Negative |
| [34](https://www.zotero.org/google-docs/?WKbdGG) | Ding, Cao & Sun (2023) | Mixed review | Problematic internet use | Social anxiety | Not discussed | The overall correlation between problematic internet use and social anxiety was significantly positive. Social anxiety is a predictor of PIU development. | Negative |
| [35](https://www.zotero.org/google-docs/?1PLXo3) | Harness, Domoff & Rollings (2023) | Narrative review | Social media use | Youth mental health | Problematic use, cyberbullying, social comparison, social support | Social media's effects on youth mental health are highly nuanced, contributing to well-being, ill-being, or both depending on the individual and specific experiences. Intervention-focused research is needed. | Mixed |
| [36](https://www.zotero.org/google-docs/?KrHO2P) | Hilty et al (2023) | Scoping review | Social media use | Psychopathology, behavioral health conditions, psychological well-being (depression, anxiety disorders) | Cyberbullying, sexting, suicide, oversharing, triggering posts | Shorter, purposeful use (≤3 h/day) is associated with better mood and well-being, while longer or passive use predicts depression and anxiety. Girls are disproportionately affected by depression/anxiety. | Mixed |
| [37](https://www.zotero.org/google-docs/?xIDrj8) | Jo & Jang (2023) | Other review type | Social media use | Emotional well-being | Internet-based communication | Adolescent emotional well-being on social media is defined as being happy and maintaining emotional health through internet-based relationships. Consequences are better relationships and positive affect. | Positive |
| [38](https://www.zotero.org/google-docs/?4UCbk5) | Khalaf et al (2023) | Systematic review | Social media use, social networking sites | Mental health, well-being, body image problems, cyberbullying, sleep impacts | Challenges and opportunities, humor, identity formation, creative expression | There is a modest but statistically significant association between social media use and depressive symptoms, internalizing/externalizing problems, and body image concerns. However, social media also provides social support and identity formation. | Mixed |
| [39](https://www.zotero.org/google-docs/?dmgUDe) | Khetawat & Steele (2023) | Meta-analysis | Social media use | Psychosocial distress | Stressful experiences accompanying notifications, use of informational and communication technologies | All five digital stress components (availability stress, approval anxiety, FOMO, connection overload, and online vigilance) had significant medium positive associations with psychosocial distress. | Negative |
| [40](https://www.zotero.org/google-docs/?H7hwlD) | Messena & Everri (2023) | Scoping review | Digital technologies use | Multidimensional well-being (psychological, social, physical, cognitive) | Negative digital technology use (compulsive/addictive use), negative online experiences (cyberbullying, sexual solicitation, sexting) | Studies show a bidirectional relationship where well-being dimensions predict digital technology negative use (DTNU), and DTNU contributes to psychological problems like depression and anxiety. | Negative |
| [41](https://www.zotero.org/google-docs/?mSdyYY) | Moss, Wibberley & Witham (2023) | Scoping review | Instagram use | Self-harm | Negative experiences, sense of belonging | Time spent on Instagram is related to deliberate self-harm (DSH). Instagram use can lead to the desensitization, normalization, and social contagion of DSH, though it also provides a sense of belonging. | Negative |
| [42](https://www.zotero.org/google-docs/?K8hxdl) | Paulus et al (2023) | Other review type | Screen media activity (general screen time, social media use, gaming, watching TV) | Mental health-related outcomes (sleep patterns, mood disturbances, anxiety, cognitive processes) | General categories of screen media activities (watching TV, gaming, social media) | Excessive screen exposure is generally associated with negative mental health effects, poor sleep, and complex cognitive impacts. The relationship is influenced by numerous moderating factors. | Mixed |
| [43](https://www.zotero.org/google-docs/?v41b25) | Popat & Tarrant (2023) | Other review type | Social media use | Mental health, psychological well-being | Cyberbullying, exposure to harmful content, connection with peers, support networks, positive online challenges | Social media impacts mental health through self-expression, appearance comparison, pressure to stay connected, peer support, and exposure to bullying. It has both negative and positive impacts. | Mixed |
| [44](https://www.zotero.org/google-docs/?u0oFjZ) | Priftis & Panagiotakos (2023) | Narrative review | Excessive screen time viewing | Physical and mental health effects (internalizing symptoms, externalizing symptoms, suicidal behaviors) | Negative impacts on physical and mental health | Excessive screen time viewing is correlated with increased risk for obesity, cardiometabolic risk factors, unhealthy dietary habits, eating disorders, mental health issues (internalizing and externalizing symptoms), and problems in child-parent relationships. Sleep, physical activity, eyesight, headaches, and the musculoskeletal system were also negatively affected. | Negative |
| [45](https://www.zotero.org/google-docs/?pQC8sd) | Santos et al (2023) | Systematic review | Screen time and exposure (TV, video games, smartphones) | Mental health, flourishing life-satisfaction, well-being, internalizing and externalizing behaviors | Not specified | Social media use was negatively associated with mental well-being and, in girls, associated with a higher risk for depression. Excessive screen time in adolescents seems associated with mental health problems. The most used device was the smartphone, and its use on weekdays was associated with diminished mental well-being. | Negative |
| [46](https://www.zotero.org/google-docs/?WElJqx) | Susi et al (2023) | Systematic review | Internet, social media | Self-harm, suicidal behaviors | Self-harm, suicide-related images | Viewing self-harm-related images online has harmful effects including escalation of self-harm, reinforcement of engagement behaviors, encouragement of social comparison, development of a self-harm identity, and emotional/cognitive triggering of self-harm urges. Protective effects were also found, including self-harm mitigation, promotion of recovery, and social connection. However, harmful effects predominated. | Mixed |
| [47](https://www.zotero.org/google-docs/?yZCUCi) | Ye, Zhang & Zhao (2023) | Meta-analysis | Internet addiction (gaming, social media, shopping, smartphone use) | Depression | Not discussed | Depression is positively correlated with Internet addiction (IA) and vice versa. Adolescents with depressive disorders have a higher risk of IA, and adolescents with IA have a higher risk of depressive disorders. IA had a stronger effect on depression risk. | Negative |
| [48](https://www.zotero.org/google-docs/?bLL30p) | Ahmed et al (2024) | Mixed review | Social media use, problematic social media use | Anxiety, depression, wellbeing, eating disorders, sleep problems | Negative impacts on mental health and sleep | Small but significant positive associations between social media use and depression and anxiety. Problematic social media use (PSMU) was positively associated with depression, anxiety, and sleep problems, and negatively associated with wellbeing. | Negative |
| [49](https://www.zotero.org/google-docs/?z7nR48) | Dun-Campbell et al (2024) | Umbrella review | Social media | Anxiety, depression, self-harm, suicide | Platform engagement, user-generated content | High-quality reviews found associations between social media use and depression, suicidal ideation, and self-harm. The review also found associations between mental ill health and other commercial determinants (alcohol, tobacco, gambling, ultra-processed foods, air pollution). | Negative |
| [50](https://www.zotero.org/google-docs/?DAgdC9) | Engel et al (2024) | Scoping review | Social media influencers | Mental health | Health information, nutrition, appearance, self-diagnosis | Social media influencers (SMIs) serve as important sources of health information but pose challenges due to lack of expertise and commercial interests. Most studies focused on negative roles, such as promoting unrealistic body images, unhealthy diets, substance use, and inaccurate diagnosis/treatment advice. Inappropriate advertising of unhealthy products was a major challenge. | Negative |
| [51](https://www.zotero.org/google-docs/?Hp5x4V) | Ferguson et al (2024) | Meta-analysis | Time spent on social media | Anxiety, depression | Negative impacts | The current pool of research is unable to support claims of harmful effects of social media use on youth internalizing disorders (anxiety, depression). The mean effect of social media on mental health across studies was near zero and below the threshold for evidentiary value. | Mixed |
| [52](https://www.zotero.org/google-docs/?x4U75j) | Giancola, Bentley & Torous (2024) | Narrative review | Social media use | Suicidal thoughts and behaviours | Mixed effects | The relationship between social media use and suicide risk in youth remains unclear and highly nuanced. Reviews show contradictory associations, with some finding positive associations and others finding no statistical significance. Social media can act as a vehicle for connection and resources, or as a trigger and reinforcement of self-injurious behaviors. | Mixed |
| [53](https://www.zotero.org/google-docs/?upkYRh) | Girela-Serrano et al (2024) | Systematic review | Mobile phones and wireless devices | Internalizing symptoms (anxiety, depression, suicidal ideation/self harm), externalizing symptoms (attention, concentration) and general well-being | Notes that none of the reviewed studies probed for specific uses of social media (e.g., interpersonal support, social comparison). Unless the specific type of data and content viewed by children and adolescents on social media (and other online activities using MP/WD) is analysed, much of the commentary on the mechanism by which usage might affect mental health remains conjecture | Suggestive but limited evidence that greater use of mobile phones and wireless devices (MP/WD) may be associated with poorer mental health. Bedtime MP/WD duration/frequency was associated with worse mental health. General MP/WD use might be associated with externalizing symptoms. Substantial between-study heterogeneity limits general conclusions. | Negative |
| [54](https://www.zotero.org/google-docs/?EJYp8m) | Hall (2024) | Narrative review | Focuses on typical and mundane, everyday, uses of social media. Social media = platforms that enable searchable and publicly distributed content, including text, photos, memes, and videos . Social media are often identified by a branded platform name (eg, Instagram). Users create content and this content is transmitted in a decentralized fashion. | Effects of social media on the well-being and ill-being of users as an aggregate - both eudaemonic & hedonic well-being. Notes that ill-being is often measured as the presence of mental health symptoms e.g. depression or loneliness. | Typical and mundane uses of social media, digital detox, problematic interactive media use, using SM to cope with negative emotions. | Time spent on social media does not have a strong effect on the well-being of its users. Preexisting vulnerabilities (e.g., poverty, mental health) are associated with both adolescent social media use and adolescent ill-being. Declines in well-being precede increased future social media use, rather than the reverse. Social media use explains very little variance in well- and ill-being compared to other factors. | Mixed |
| [55](https://www.zotero.org/google-docs/?aM0KSv) | Jaycox et al (2024) | Narrative review | SM use - aligning with Surgeon General's definition "internet-based channels that allow users to opportunistically interact and selectively self-present, either in real-time or asynchronously, with both broad and narrow audiences who derive value from user-generated content and the perception of interaction with others.” | Suicide behaviour - include suicide ideation, suicide intent, suicide attempts, and death by suicide | Cyberbullying, self-harm websites, suicide-related content, social connections, help-seeking behaviors. | Current research shows complex and conflicting relationships between social media use and suicide risk. A key limiting factor is a dearth of information on contextual factors around social media use. While there are clear associations between some types of SM use and suicide risk, there are also many opportunities to mitigate suicide risk through social media. | Mixed |
| [56](https://www.zotero.org/google-docs/?vpkGak) | Liu et al (2024) | Systematic review | General screen media use: intentional use of various types of media (e.g., phone, computer), content (e.g., entertainment, education), and technologies (e.g., social media applications). Review included studies on internet use, social media use and phone use. | Mental health: Internalizing problems characterized by any emotional disturbance (e.g., depression, anxiety, loneliness) and its associated behavioral manifestations (e.g., suicidal ideation and attempts). Studies included outcomes of anxiety/depression, emotional well-being, suicidal behaviour, loneliness, FOMO, self-esteem. | Social media use, smartphone use, internet use, active screen media use. | Elevated screen media use has a significant negative association with adolescents' mental health, but mostly with small-to-modest effect sizes. Positive family processes (e.g., parent-teen relationship quality) and democratic media-specific parenting mitigate such association. Adolescents in families with low SES have elevated SM use and mental health risks. | Negative |
| [57](https://www.zotero.org/google-docs/?c3FkZe) | McGorry et al (2024) | Other review type | (Unregulated) social media | Mental health | General social media use. | The mental health of emerging adults has been declining steadily over the past two decades. Global megatrends, including unregulated social media, rising inequality, and climate change, have harmed youth mental health. Social media is one potent element in a wider set of harmful megatrends. A new field of youth mental health is needed with integrated primary youth mental health care and digital technologies to drive service innovation. | Negative |
| [58](https://www.zotero.org/google-docs/?9calIM) | National Academies of Sciences, Engineering and Medicine (2024) | Other review type | Social media | Psychological outcomes, sleep problems | Social comparison, cyberbullying, sexual exploitation, fanfiction, gaming, support | The review did not support the conclusion that social media causes changes in adolescent health at the population level, but there are potential harms (e.g., unhealthy social comparisons, displacing sleep) and benefits (e.g., connection, learning). The relation between social media and health is complex, bidirectional, and varies among individuals. | Mixed |
| [59](https://www.zotero.org/google-docs/?ig6XjR) | Sanders et al (2024) | Umbrella review | Use of electronic screens (television, mobile phones & computers) | Looks at outcomes for education and health, including depression | Digital advertising of unhealthy foods  Social media use  Sexual content  Screen-based health interventions | Meta-analyses of associations between screen use and outcomes showed small-to-moderate effects. General screen use was negatively associated with literacy and learning, but educational content or co-viewing had positive associations. Social media was consistently associated with poor health (e.g., depression, risk taking) with no indication of potential benefit. Screen-based health interventions were effective. | Mixed |
| [60](https://www.zotero.org/google-docs/?nZQhvm) | Sewall & Parry (2024) | Other review type | Social media use | Youth mental health | General social media use. | The prevailing narrative that social media use is the principal cause of declining youth mental health oversimplifies a complex phenomenon. Misspecified models that ignore confounding factors, such as psychosocial adversity, can lead to biased conclusions about social media's adverse effects. Focus on social media diverts attention from broader factors contributing to youth mental health problems. | Mixed |
| [61](https://www.zotero.org/google-docs/?xOuW2R) | Weigle & Shafi (2024) | Narrative review | Social media | Youth mental health - specific diagnoses including depression and anxiety | Includes FOMO, social comparison, cyberbullying, sexting and contagion  Users who experience greater SM-induced social comparison tend to experience a decline in well-being, a consistent finding in cross-sectional, longitudinal, and experimental studies. | The mental health of youth is increasingly tied to their SM use, depending greatly on how youth engage with SM and resultant feedback. Specific risks include displacement of healthier habits, cyberbullying, sexting, social comparison, passive use patterns, and mental health contagion, while benefits include access to social support. | Mixed |
| [62](https://www.zotero.org/google-docs/?UX9ilI) | Yu et al (2024) | Scoping review | Social media use - engaging with online platforms specifically designed for social interaction | Mental health | Social comparison, bullying, extensive negative feedback  Use of social media including as a sleep aid | Both cross-sectional and longitudinal studies demonstrated the negative impacts of SM use on sleep and mental health, though preliminary evidence indicated potential benefits during the COVID-19 period. Limited longitudinal research hinders establishing directionality and causality. | Negative |
| [63](https://www.zotero.org/google-docs/?u7tn44) | Zhang et al (2024) | Meta-analysis | Problematic internet use, a type of behavioural addiction - characterized by excessive or ineffectively managed urges and behaviors related to Internet use that cause subjective distress and/or interfere with essential life functions. Encompasses sub-cateogories such as SM addiction, gaming addiction, smartphone addiction | Loneliness - a distressing feeling that arises when an individual’s social needs are not met by their social connections, associated with physical and mental health issues | Problematic internet use, social media addiction, gaming addiction, smartphone addiction, pornography addiction. | There is a moderate, positive, and significant bidirectional relationship between loneliness and problematic internet use (PIU) over time. The association was stronger in samples from Eastern countries, with more males, more young adults, and in more recent years. | Negative |

**Table S2. Summary of included studies (individual studies)**

| **Study ID** | **Author name (year)** | **Study design** | **Exposures studied** | **Outcomes studied** | **Types of online content, behaviours or experiences studied** | **Key findings** | **Direction of evidence** |
| --- | --- | --- | --- | --- | --- | --- | --- |
| [64](https://www.zotero.org/google-docs/?32TMKi) | Barthorpe et al (2020) | Cohort | Time spent on social media | Self-harm in the past year, depressive symptoms (Short Mood and Feelings Questionnaire), Self-esteem | Browsing and updating on social networking sites | A greater amount of time spent on social media was associated with an increased risk of self-harm and depression, and lower levels of self-esteem in females. | Negative |
| [65](https://www.zotero.org/google-docs/?n1MFDj) | Bennett et al (2020) | Ecological momentary assessment | Total number of social media sites visited, total time spent on social media | Body dissatisfaction, general negative affect, guilt, and sadness | Not specified | The number of social media sites visited was a significant predictor of body dissatisfaction. Both number of sites and time spent were significant predictors of general negative affect, sadness, and guilt. | Negative |
| [66](https://www.zotero.org/google-docs/?t6zzyd) | Beyens et al (2020) | Within-person study | Top 3 platforms, passive vs. active use | Affective well-being | Active and passive use | The association between social media use and affective well-being differs strongly across adolescents: 44% did not feel better or worse after passive social media use, 46% felt better, and 10% felt worse. | Mixed |
| [67](https://www.zotero.org/google-docs/?wFg9TG) | Boer et al (2020a) | Cross-sectional | Intense social media use (time spent) and problematic social media use (symptoms of addiction) | Mental (life satisfaction, psychological complaints), school, and social well-being | Intense and problematic social media use | Problematic SMU is associated with lower well-being. Intense SMU is associated with lower well-being in countries with lower prevalence of intense SMU, but higher well-being in countries with higher prevalence. | Mixed |
| [68](https://www.zotero.org/google-docs/?0AwMhA) | Boer et al (2020b) | Three-wave longitudinal study | Social media use intensity (frequency) and social media use problems (addiction-like symptoms) | Mental health (depressive symptoms and life satisfaction) | Upward social comparisons, cybervictimization, subjective school achievements, and face-to-face contact with friends | SMU problems were associated with decreased mental health one year later, but not vice versa. SMU problems also predicted increased levels of upward social comparisons and cybervictimization. SMU intensity and mental health were not associated in any direction. | Negative for problematic use; Neutral for intensity |
| [69](https://www.zotero.org/google-docs/?TSjApC) | Coyne et al (2020) | 8-year longitudinal study | Time spent using social networking sites (SNSs) | Mental health (depression and anxiety) | Time spent on social media | Increased time spent on social media was not associated with increased depression or anxiety across development when examined at the individual level. Social networking did not predict future anxiety or depression, and vice versa. | Neutral |
| [70](https://www.zotero.org/google-docs/?VPVWrE) | Craig et al (2020) | Cross-sectional study | Social media use (intense use, problematic use, and frequent online contact with strangers) | Cyber-bullying (victimization and perpetration) | Intense use, problematic use, and frequent online contact with strangers | Engagement in all three types of social media use related to cyber-bullying victimization and perpetration. Problematic use was most strongly and consistently associated with cyber-bullying. Associations were stronger for cyber-perpetration than victimization, and for girls than boys. | Negative |
| [71](https://www.zotero.org/google-docs/?KuzJaJ) | Das-Friebel et al (2020) | Experience sampling methodology (14 days) | Bedtime social media use | Sleep duration, sleep satisfaction, positive and negative affect, depressive symptoms | Bedtime social media use | Increased bedtime social media use was not associated with poorer sleep the same night. No association was found between bedtime social media use and positive or negative affect the following day. For participants with higher depressive symptoms, bedtime social media use was negatively associated with subjective sleep duration. | Mixed |
| [72](https://www.zotero.org/google-docs/?NmywDN) | Escobar-Viera et al (2020) | Cross-sectional qualitative content analysis | Social media use | Mental well-being | Social connectedness, social capital, emotional support, negative emotional contagion, comparison with others, real-life repercussions, envy, profile management | Most responses described positive SM effects, such as gaining social capital for LGB users. However, LGB individuals reported more negative SM experiences like negative emotional contagion, comparison with others, and real-life repercussions. Increased negative SM experiences may pose a risk for the mental well-being of LGB individuals. | Mixed |
| [73](https://www.zotero.org/google-docs/?CY5V8a) | Fabris et al (2020) | Cross-sectional | Social media use, social media addiction | Emotional symptoms, Fear of Missing Out (FoMO), stress associated with neglect and negative reactions | Neglect (not receiving likes/comments) and negative reactions by online peers | Fear of missing out (FoMO) directly and indirectly predicts emotional symptoms. FoMO is associated with increased sensitivity to stress from neglect by online peers, which predicts social media addiction. Social media addiction mediates the relationship between FoMO and emotional symptoms. | Negative |
| [74](https://www.zotero.org/google-docs/?WqTWds) | Fardouly et al (2020) | Cross-sectional | Time spent on YouTube, Instagram, Snapchat, Facebook | Body satisfaction, eating pathology, depressive symptoms, social anxiety | Appearance comparisons, appearance investment, selfie posting, receiving likes | Users of YouTube, Instagram, and Snapchat reported more body image concerns and eating pathology than non-users. Appearance comparisons uniquely predicted all aspects of mental health (body satisfaction, eating pathology, social anxiety, depressive symptoms). Appearance investment uniquely predicted depressive symptoms. | Negative |
| [75](https://www.zotero.org/google-docs/?dAdzxF) | Gassó, Mueller-Johnson & Montiel (2020) | Cross-sectional | Sexting behaviors (active and passive), online sexual victimization (OSV) | Global psychopathology, anxiety, depression | Creating, sending, and receiving sexual content, nonconsensual dissemination, being pressured or threatened to sext | For male participants, online sexual victimization was associated with psychopathology, but consensual sexting was not. For female participants, active sexting, passive sexting, and online sexual victimization were all associated with poorer mental health. | Negative |
| [76](https://www.zotero.org/google-docs/?WAtzp7) | George et al (2020) | Cross-sectional | Digital technology access and use (Internet access, mobile phone ownership, social media account, frequency of social media use), perceived technology-related impairments | Academic achievement (math and reading test scores), school belonging, conduct problems, psychological distress, general physical health | Perceived technology-related impairments, online-to-offline spillover of negative experiences (e.g., face-to-face arguments, physical fights, getting into trouble at school) | Mobile phone ownership was not associated with any indicators of well-being after controlling for demographic factors. Having a social media account and frequency of social media use were only robustly associated with conduct problems. 91% of adolescents reported at least 1 perceived technology-related impairment and 29% reported online-to-offline spillover of negative experiences. Economically disadvantaged adolescents reported greater online-to-offline spillover and stronger associations between social media account ownership and poor psychological well-being compared with more affluent peers. | Mixed |
| [77](https://www.zotero.org/google-docs/?BA7tqD) | Hawes, Zimmer-Gembeck & Campbell (2020) | Cross-sectional | Social media use (time spent, intensity), general and appearance-related (AR) social media preoccupation | Depression, social anxiety, appearance anxiety, appearance rejection sensitivity (appearance-RS) | Appearance-related social media activities and content (e.g., appearance comparisons and judgements) | Social media use was positively associated with symptoms of depression, social anxiety, appearance anxiety, and appearance-RS. General and AR preoccupation had unique and positive associations with depression and social anxiety symptoms and with appearance sensitivities. AR preoccupation strengthened the relationship between time spent on social media and appearance-RS. No evidence that gender moderated the effects of AR social media preoccupation. | Negative |
| [78](https://www.zotero.org/google-docs/?2WEXQF) | Kim et al (2020) | Cross-sectional | Passive screen time (watching TV, movies, videos) and active screen time (video gaming, chatting, working on a computer) | Major depressive episode, social phobia, generalized anxiety disorder, specific phobia | Passive and active forms of screen time | Adolescents reporting 4 or more hours of passive screen time per day were three times more likely to meet the criteria for major depressive episode, social phobia, and generalized anxiety disorder. Passive screen time continued to be significantly associated with increased odds of disorders after adjusting for covariates. Associations between active screen time use and depression and anxiety disorders were smaller in magnitude and failed to reach statistical significance. | Negative |
| [79](https://www.zotero.org/google-docs/?qMOQFo) | Kvardova, Machackova & Smahel (2020) | Cross-sectional | Health-oriented websites (nutrition, weight loss, and exercise websites) | Drive for thinness | Web content internalization of body appearance standards, perceived online social support | Positive indirect link between both perceived online social support and neuroticism to the drive for thinness via web content internalization. Perceived online social support from health-oriented websites predicted web content internalization, which in turn increased the drive for thinness. Online social support can function as both a risk and a protective factor. | Negative |
| [80](https://www.zotero.org/google-docs/?eBPsM7) | Mars et al (2020) | Longitudinal | Internet use (hours online) | Depression, anxiety, self-harm | Visiting unmoderated chatrooms, being bullied online, receiving unwanted sexual comments, coming across pornography, being sent unsolicited sexual material, coming across violent/gruesome images, coming across racist/hateful material, meeting someone face to face, having personal information misused | Females reporting high levels of internet use (hours online) were at increased risk of depression at follow-up, whereas males with high levels of internet use were at increased risk for self-harm. No association between hours spent online and anxiety. For females, being bullied online and meeting someone face to face were associated with an increased risk of future depression. Being bullied online, receiving unwanted sexual comments or material, and coming across pornography and violent/gruesome material were associated with an increased risk of future self-harm. | Negative |
| [81](https://www.zotero.org/google-docs/?VBd382) | O'Reilly (2020) | Qualitative study (focus groups) | Social media use | Mental health (low self-esteem, anxiety, depression, poor sleep, self-harm, suicidal ideation) | Cyberbullying, trolling, self-harm/suicide content, communication, connection | Participants discussed the "good", "bad", and "ugly" sides of social media. Social media can reduce isolation and improve social skills, but also cause pressure, poor sleep, and exposure to cyberbullying and self-harm content. | Mixed |
| [82](https://www.zotero.org/google-docs/?Uz1ADI) | Peris et al (2020) | Cross-sectional study | Social networking and internet addiction (internet addiction symptoms, social media use, geek behaviour, nomophobia) | Internet addiction symptoms, social media use, geek behaviour, nomophobia | Social media use, geek behaviour (gaming, eroticism), nomophobia | Gender and disinhibition predicted addiction symptoms. Gender and physical attractiveness predicted social media use. Narcissism and neuroticism predicted geek behaviour and nomophobia. | Negative |
| [83](https://www.zotero.org/google-docs/?bwqUEJ) | Prichard et al (2020) | Experimental study | Instagram #fitspiration images vs travel images | State body dissatisfaction and mood, exercise behaviour | Fitspiration images (women in workout clothing, before-and-after transformations) | Exposure to fitspiration images led to higher negative mood and body dissatisfaction compared to travel images. No difference in actual exercise behaviour, but higher subjective exertion. | Negative |
| [84](https://www.zotero.org/google-docs/?XhjvfT) | Puukko et al (2020) | Longitudinal study (5 waves) | Active social media use | Depressive symptoms | Active, socially-oriented use (chatting, sharing photos, status updates) | Depressive symptoms predicted small increases in active social media use, but no evidence of the reverse relationship. Associations were very small and somewhat inconsistent. | Mixed |
| [85](https://www.zotero.org/google-docs/?N4OiH5) | Shensa et al (2020) | Cross-sectional study | Social media-based emotional support | Depression risk | Emotional support via social media | Face-to-face emotional support was associated with lower odds of depression, but social media-based emotional support was associated with 20% greater odds of depression. | Negative |
| [86](https://www.zotero.org/google-docs/?s4K8Kr) | Twenge & Farley (2020) | Cross-sectional study | Time spent on specific screen media activities (social media, internet, gaming, TV) | Self-harm behavior, depressive symptoms, life satisfaction, self-esteem | Social media, internet, gaming, TV | Hours spent on social media and internet use were more strongly associated with self-harm, depressive symptoms, low life satisfaction, and low self-esteem than gaming and TV. Girls demonstrated stronger associations than boys. | Negative |
| [87](https://www.zotero.org/google-docs/?njQlSl) | Twenge & Martin (2020) | Cross-sectional study | Digital media use (social media, texting, general computer use, gaming, smartphone use) | Psychological well-being, mental health issues (suicide risk factors, depression, happiness) | Social media, texting, gaming, internet, TV | Girls spent more time on smartphones, social media, and texting; boys spent more time gaming. Associations between heavy digital media use and low well-being/mental health issues were generally larger for girls than for boys. | Negative |
| [88](https://www.zotero.org/google-docs/?dIE1gR) | Twigg, Duncan & Weich (2020) | Longitudinal study (7 waves) | Social media use (chatting or interacting with friends) | Life satisfaction (happiness) | Chatting/interacting with friends on social web-sites | High use of social media (4+ hours) was significantly associated with a decline in happiness scores, especially for girls, but not associated with worsening life satisfaction trajectories overall. Moderate use was not associated with changes. | Negative |
| [89](https://www.zotero.org/google-docs/?RljK3v) | Beeres et al (2021) | Longitudinal study (3 waves) | Frequency of social media use | Mental health problems (internalizing and externalizing symptoms, SDQ) | Social media, blogs, chat online | Adolescents with higher social media use reported more symptoms of mental ill-health (between-person). However, on a within-person level, there were no longitudinal associations between changes in social media use and subsequent changes in mental health or vice versa. | Mixed |
| [90](https://www.zotero.org/google-docs/?hwuClz) | Belton et al (2021) | Cross-sectional study | Leisure screen time (TV, video games, smartphone/tablet) | Wellbeing (KIDSCREEN-27: physical, psychological, parental, peers, school) | Leisure screen time | Children with <2 hours of leisure screen time scored significantly higher on physical, parental, peers, and school wellbeing, but not psychological wellbeing. No significant interaction between screen time and gender on overall wellbeing. | Negative |
| [91](https://www.zotero.org/google-docs/?eAQCHP) | Beyens et al (2021) | Experience sampling method (ESM) study, longitudinal (3 weeks) | Active private, passive private, and passive public social media use | Momentary affective well-being (happiness) | Active private (sending direct messages), passive private (reading direct messages), passive public (viewing posts/stories of others) | 45% no changes in well-being, 28% only declines, 26% only increases. | Mixed |
| [92](https://www.zotero.org/google-docs/?hihmcQ) | Buda et al (2021) | Cross-sectional | Problematic social media use | Physical activity levels, sleep peculiarities, and life satisfaction | Problematic social media use | Problematic social media use associates with worse sleep quality and lower life satisfaction. | Negative |
| [93](https://www.zotero.org/google-docs/?04jhzn) | Charmaraman et al (2021) | Cross-sectional | Social media use, following celebrities | Body dissatisfaction, depressive symptoms, online social anxiety, making new friends, social isolation | Comparing self with what was seen on social media related to body image/shape/type, viewing celebrity photos, friend photos | Social media-related body dissatisfaction associated with higher depressive symptoms, online social anxiety, harder to make friends. | Negative |
| [94](https://www.zotero.org/google-docs/?grNgZY) | Coyne et al (2021) | Longitudinal (10 years) | Screen time (social media, television, video games, cell phone use) | Suicide risk | Passive sensing app categories (entertainment, games, social networking, etc.), cyberbullying, sexting, positive/negative use of media | High and increasing social media use predictive of suicide risk for girls. | Negative |
| [95](https://www.zotero.org/google-docs/?0TLkq9) | Dissing et al (2021) | Longitudinal (4 months) | Nighttime smartphone use | Loneliness, depressive symptoms, perceived stress, and life satisfaction | Smartphone activities during sleep period | Nighttime smartphone use not strongly related to poor mental health. | Neutral |
| [96](https://www.zotero.org/google-docs/?2DeWu9) | Ferguson (2021) | Longitudinal | Screen time, social media use | Suicidal depression, emotional problems | Screen time, social media use | No evidence that effect sizes between screen/social media use and depression are increasing over time. | Neutral |
| [97](https://www.zotero.org/google-docs/?rcmoTf) | Hamilton et al (2021) | Prospective longitudinal (30 days) | Social media use, messaging | Suicidal ideation with and without a plan, suicidal behavior, depressive symptoms | Social media use, direct messaging/texting | Lower levels of SM use associated with greater likelihood of suicidal ideation with plan. | Positive |
| [98](https://www.zotero.org/google-docs/?G5Q5lv) | Hjetland et al (2021) | Qualitative (focus groups) | Social media use | Mental health and well-being | Social connectedness, upward social comparison, body pressure, cyberbullying | Social media has both positive and negative impacts on mental health. | Mixed |
| [99](https://www.zotero.org/google-docs/?zKp38t) | Jarman et al (2021a) | Cross-sectional | Social media engagement (intensity and appearance-focused use) | Body satisfaction and subjective well-being | Appearance-focused social media use (Instagram and Snapchat) | Higher appearance-focused social media use directly associated with lower body satisfaction and well-being. | Negative |
| [100](https://www.zotero.org/google-docs/?Zmit8R) | Jarman et al (2021b) | Cross-sectional | Social media engagement (intensity, photo-based use, active use, passive use and liking use) | Body satisfaction and well-being | Motivations for social media use (information sharing, passing time, escapism, social interaction, social capital and appearance feedback) | Motivations for SM use associated with engagement and mixed associations with body satisfaction and well-being. | Mixed |
| [101](https://www.zotero.org/google-docs/?l2t2UM) | Khan et al (2021) | Cross-sectional observational study | Screen time (watching TV, gaming, using computers/electronic devices for other purposes like emailing, social media, chatting, surfing internet) | Mental wellbeing (life satisfaction, psychosomatic health complaints) | Time spent on screens, physical activity | Higher levels of screen time and lower levels of physical activity were associated with lower life satisfaction and higher psychosomatic complaints among adolescents from high-income countries. Detrimental associations between screen time and mental wellbeing started when screen time exceeded 1 h per day. Spending more than 1 h to 2 h per day on screens, as opposed to 1 h or less per day, was positively associated with life satisfaction in boys. | Mixed |
| [102](https://www.zotero.org/google-docs/?fnFAFZ) | Kleppang et al (2021) | Cross-sectional survey | Electronic media use (social media and gaming) | Symptoms of depression | Time spent on social media and gaming | The odds of having symptoms of depression were higher for those who used social media more than 3 hours per day. Additionally, the odds of having symptoms of depression was higher for those who used more than 3 hours on gaming per day compared to those who used 3 hours and less. | Negative |
| [103](https://www.zotero.org/google-docs/?BMUov5) | Kohler, Turner & Webster (2021) | Experimental study | Viewing appearance-focused accounts on Instagram (fitness, model, beauty-blogging) vs control photos | Mood (positive, negative), state anxiety | Upward social comparison, viewing image-conscious photos | Students viewing image-conscious Instagram accounts (vs. control) reported decreases in positive mood and increases in negative mood and state anxiety. | Negative |
| [104](https://www.zotero.org/google-docs/?gFtnK1) | Kürtüncü, Ayyıldız & Kurt (2021) | Cross-sectional observational study | Smartphone addiction | Loneliness | Smartphone use for calls, texting, research, social media | As the level of addiction to smartphones rose, the percentage of students feeling lonely dropped. Although the students' smartphone addiction was low, the rate of using the smartphone to be relieved of loneliness and for the purpose of the communication was high. | Positive |
| [105](https://www.zotero.org/google-docs/?sen8cJ) | Kvardova et al (2021) | Cross-sectional survey | Harmful online content (self-harm, suicide, eating disorders, hate messages, drug use, violence) | Emotional problems | Exposure to harmful online content | Emotional problems and sensation seeking were associated with increased exposure to harmful online content, whereas quality of family environment was associated with decreased exposure to harmful online content. | Negative |
| [106](https://www.zotero.org/google-docs/?GrP4sn) | MacDonald & Schermer (2021) | Cross-sectional survey | Smartphone use (screen time, pickups, application use) | Loneliness | Screen time, pickups, social media app use, communication app use | Loneliness was positively associated with screen time, social media app use. Loneliness was negatively associated with smartphone pickups, communication application use. More time spent on one's smartphone and on social media apps is related to increased loneliness. More frequent use (pickups) and use of communication apps is related to decreased loneliness. | Mixed |
| [107](https://www.zotero.org/google-docs/?wH5u6e) | Marengo et al (2021) | Cross-sectional survey | Problematic social media use (PSMU) | Cyberbullying victimisation and perpetration | Problematic social media use | PSMU was positively associated with cyberbullying victimisation in all five models, with the strongest association observed in girls. The risk of cyberbullying perpetration with PSMU was significantly higher in girls than in boys. | Negative |
| [108](https://www.zotero.org/google-docs/?hqBaOB) | McComb & Mills (2021) | Experimental study | Viewing idealized body pictures on Instagram (upwards comparison) | State body perception (weight dissatisfaction, appearance dissatisfaction, confidence) | Upwards appearance comparison | Appearance comparison to the models resulted in lowered confidence and increased appearance and weight dissatisfaction. | Negative |
| [109](https://www.zotero.org/google-docs/?ENfqhU) | Mundy et al (2021) | Longitudinal study | Social networking use duration | Depressive and anxiety symptoms | Time spent on social networking | Females with high social networking use had greater odds of depressive and anxiety symptoms than those that used a few minutes at most, while males with high social networking use had 1.60 greater odds of reporting depressive symptoms. For females, an increased odds of depressive symptoms at age 14.8 was observed for high social networking use at one previous wave and at two or three previous waves. | Negative |
| [110](https://www.zotero.org/google-docs/?CDYUJd) | Paakkari et al (2021) | Cross-sectional survey | Problematic social media use | Health complaints (headache, neck and shoulder pain, feeling low, nervousness, irritability), physical inactivity, loneliness, low self-rated health, morning tiredness, short sleep | Problematic social media use | All the negative health indicators systematically increased if the respondent belonged to a moderate risk or problematic use group. | Negative |
| [111](https://www.zotero.org/google-docs/?lDwVvC) | Parsons, Alden & Biesanz (2021) | Experimental | Instagram use | Positive affect, negative affect, state self-esteem, social rank self-perceptions | Instagram influencer profiles | Higher social anxiety predicted lower, and greater declines in, social rank self-perceptions during browsing. Higher social anxiety also predicted greater decreases in positive affect, increases in negative affect, and decreases in state self-esteem from the beginning to the end of the browsing task. | Negative |
| [112](https://www.zotero.org/google-docs/?pW9uE7) | Paulich et al (2021) | Cross-sectional | Total screen time (TV, videos, video games, texting, social media, video chat) | Anxiety and depression symptoms, composite internalizing problems, composite externalizing problems, attention problems, ADHD, academic performance, sleep patterns, and peer relationships | General screen time | More screen time is moderately associated with worse mental health, increased behavioral problems, decreased academic performance, and poorer sleep, but heightened quality of peer relationships. However, effect sizes associated with screen time and the various outcomes were modest; SES was more strongly associated with each outcome measure. | Mixed |
| [113](https://www.zotero.org/google-docs/?j1nRnv) | Petropoulos Petalas et al (2021) | Cross-sectional | Social Media Use (number of platforms, time spent, number of followers, followees, shared online/offline friends, CoolRatio) | Mental health (well-being: happiness, confidence, life satisfaction; ill-being: stress, anxiety, sleep onset latency) | General social media use | Time spent on social media appeared as the most robust predictor of MH decrease. At the same time, a large number of shared online and real-life friends appears to increase MH. Small yet significant associations between SMU and MH were noted, explained both by linear and by quadratic functions. | Mixed |
| [114](https://www.zotero.org/google-docs/?GwwKqk) | Pouwels et al (2021) | Experience sampling method (ESM) | Instagram, WhatsApp, and Snapchat use (time spent in previous hour, use with/without close friends) | Momentary experiences of friendship closeness | Social media use with and without close friends | Positive between-person associations of friendship closeness with general WhatsApp use and Instagram use with close friends. In contrast, at the within-person level, we found small negative overall associations of general WhatsApp use and Instagram use (with and without close friends) with friendship closeness. | Mixed |
| [115](https://www.zotero.org/google-docs/?xgy3UY) | Shafi et al (2021) | Experimental | Acute social media use (20 minutes of unsupervised SMU) | Clinical measures (depressive symptom severity, self-esteem) and biomarkers of stress (salivary alpha-amylase and cortisol levels) | General social media use | After 20 min of SMU, salivary cortisol and alpha-amylase levels were significantly higher in adolescents with depression but not in healthy control adolescents. Small but statistically significant changes in depressive symptom severity occurred in all participants, but were not clinically meaningful. | Negative |
| [116](https://www.zotero.org/google-docs/?8HquKC) | Skogen et al (2021) | Cross-sectional | Focus on self-presentation on social media | Symptoms of anxiety and depression and quality of life | Self-presentation on social media | A high focus on SOME self-presentation was associated with more mental health problems and reduced quality of life. The association was similar across gender in relation to symptoms of anxiety. For symptoms of depression and quality of life, the association was stronger for girls compared to boys. | Negative |
| [117](https://www.zotero.org/google-docs/?Y6TzVD) | Tamarit et al (2021) | Cross-sectional | Internet addiction and problematic use of social media (addiction symptoms, social media use, geek behavior, nomophobia) | Sexual online victimization (erotic and coercive sextortion and grooming) | Sexting, sextortion, grooming | Internet addiction predicts online sexual victimization; specifically, the best predictors of sexting, sextortion and grooming victimization were symptoms of internet addiction and geek behavior. Body self-esteem and sexting mediated the relationship between internet addiction and sexual online victimization. | Negative |
| [118](https://www.zotero.org/google-docs/?b3qFaQ) | Trumello et al (2021) | Cross-sectional | Internet addiction problems | Mental health problems (conduct problems, emotional symptoms, peer problems, hyperactivity) | Internet addiction | Perceived care from both father and mother had significant indirect effects on Internet addiction problems through adolescents' mental health problems. Furthermore, Internet addiction problems were demonstrated to be negatively associated with maternal care but not with paternal care. | Negative |
| [119](https://www.zotero.org/google-docs/?UViuqg) | Vuorre, Orben & Przybylski (2021) | Cross-sectional and Longitudinal | Technology engagement (social media use, digital device use, television viewing) | Mental health (depression, emotional problems, conduct problems, suicidal ideation and behavior) | General technology engagement, social media | Technology engagement had become less strongly associated with depression in the past decade, but social-media use had become more strongly associated with emotional problems. We detected no changes in five other associations or differential associations by sex. There is therefore little evidence for increases in the associations between adolescents' technology engagement and mental health. | Mixed |
| [120](https://www.zotero.org/google-docs/?pFhmg9) | Azhari et al (2022) | Cross-sectional | Social Media Disorder (SMD), intensity of social media activity across Facebook, Instagram, Snapchat and Twitter | Anxiety, loneliness, and sleep disturbances | Social media across platforms (Facebook, Instagram, Snapchat, Twitter) | Users with SMD experienced elevated levels of loneliness and had less sleep on average, and during the weekdays in particular. Only frequency of posting on Facebook, but not general usage, was associated with poorer sleep quality. SMD was not significantly associated with anxiety. | Negative |
| [121](https://www.zotero.org/google-docs/?Fp8BWg) | Boer et al (2022) | Longitudinal | SMU intensity and problems | Life satisfaction | Active and passive SMU | Within-person, changes in SMU were not associated with changes in life satisfaction on average. Between-person negative associations were driven by SMU problems. | Mixed |
| [122](https://www.zotero.org/google-docs/?YMUfxd) | Bohnert & Gracia (2022) | Longitudinal | Digital screen-time and type | Socioemotional well-being and academic performance | Gaming, social media, learning | Heavy digital screen-time (3+ hours/day) is associated with declines in well-being. Low-SES adolescents are more harmed, while high-SES adolescents benefit from moderate use and learning activities. | Mixed |
| [123](https://www.zotero.org/google-docs/?IZrYWx) | Boniel-Nissim et al (2022) | Cross-sectional | Intensity and problematic SMU | Mental/social well-being, substance use | Social media | Problematic users showed the least favorable mental and social well-being and highest substance use. Intense non-problematic users had the highest social well-being. | Mixed |
| [124](https://www.zotero.org/google-docs/?tHZmmB) | Castrén et al (2022) | Cross-sectional | Excessive SMU, gaming, gambling | Risk factors | Social media, gaming, gambling | Adolescent girls are at greater risk of excessive SMU than boys, while boys are at greater risk of excessive gaming/gambling. | Negative |
| [125](https://www.zotero.org/google-docs/?dV03hj) | Dissing et al (2022) | Longitudinal | Nighttime smartphone use | Mental health | Smartphone activities | Nighttime smartphone use was associated with slightly higher perceived stress and depressive symptoms at baseline, but not replicated at follow-up. | Mixed |
| [126](https://www.zotero.org/google-docs/?sSAxeN) | Erdem & Sezer Efe (2022) | Cross-sectional | Smartphone addiction | Peer relationships and loneliness | Smartphone use | Weak positive correlation between companionship and smartphone addiction. Weak negative correlation between conflict and smartphone addiction. | Mixed |
| [127](https://www.zotero.org/google-docs/?XWNNgs) | Gámez-Guadix et al (2022) | Cross-sectional | Self-harm on the internet | Depression, anxiety, family cohesion | Self-harm content | 11% of participants engaged in self-harm on the internet. Depression and anxiety increased the risk, whereas family cohesion decreased it. | Negative |
| [128](https://www.zotero.org/google-docs/?vpKNUQ) | Garcia, Bingham & Liu (2022) | Longitudinal (Daily-diary) | Daily Instagram use | State self-objectification, well-being | Instagram | Using more IG on a particular day was related to increased state self-objectification, lower life satisfaction, and higher negative mood. | Negative |
| [129](https://www.zotero.org/google-docs/?Ovlizn) | Günaydın et al (2022) | Correlational | Problematic Internet use | Emotion regulation and family attitude | Internet use | Significant correlation between problematic Internet use, emotional regulation difficulties, and family control. | Negative |
| [130](https://www.zotero.org/google-docs/?EI08tO) | Lee et al (2022) | Longitudinal | Frequent social media use | Internalizing mental health problems | Social media | Frequent social media use is associated with poorer subsequent mental health. Substantial sex differences existed in growth trajectories. | Negative |
| [131](https://www.zotero.org/google-docs/?q6J2lh) | Mann & Blumberg (2022) | Cross-sectional | Social media use | Possible self imagery | Social media | No significant differences found in the balance of possible selves based on social media use or self-esteem. Females reported more feared selves involving interpersonal relationships. | Neutral |
| [132](https://www.zotero.org/google-docs/?4t0uqw) | Marciano et al (2022) | Longitudinal | Smartphone use | Well-being | Smartphone use | Frequent and longer smartphone use predicted higher well-being, and well-being positively predicted subsequent duration of use. | Positive |
| [133](https://www.zotero.org/google-docs/?1UWbyW) | Marciano, Schulz & Camerini (2022) | Longitudinal | Duration of Internet use | Depression and social connection | Internet use | Higher duration of Internet use increased depression at the within-person level. Between-person, heavier users reported higher social connections. | Mixed |
| [134](https://www.zotero.org/google-docs/?rCs3FL) | McArthur et al (2022) | Longitudinal | Screen time | Socioemotional and developmental outcomes | TV, computer, gaming | Screen time mediated the relation between cumulative risk and child internalizing symptoms and developmental milestones. | Negative |
| [135](https://www.zotero.org/google-docs/?BA0ct7) | McCrory, Best & Maddock (2022) | Qualitative | Highly visual social media (HVSM) | Mental health | Snapchat, Instagram, TikTok | HVSM triggers an unrelenting process of emotional highs and lows. Passive viewing instigates jealousy, while active contributing yields temporary high self-esteem. | Mixed |
| [136](https://www.zotero.org/google-docs/?yufZK4) | Mehtälä et al (2022) | Qualitative | IT use experiences | Technostress | Social media, notifications, gameplay | Early adolescents encounter stressful IT experiences related to online social behavior, notifications, online education, and gameplay. | Negative |
| [137](https://www.zotero.org/google-docs/?zQ5ipE) | Miljeteig & von Soest (2022) | Longitudinal | Social media use | Self-esteem | Facebook, Instagram, Snapchat | Low initial self-esteem predicted more frequent social media use in women, but less in men. Recent social media use predicted lower current self-esteem for women. | Negative |
| [138](https://www.zotero.org/google-docs/?BdDgm4) | Moreton & Greenfield (2022) | Qualitative | Instagram use | Mental wellbeing | Instagram | Instagram was perceived as positive for maintaining friendships but negatively impacted wellbeing through presentation of ideals, cyberbullying, and search for social acceptance. | Mixed |
| [139](https://www.zotero.org/google-docs/?zHNuN3) | Orben et al (2022) | Longitudinal/Cross-sectional | Social media use | Life satisfaction | Social media | Distinct developmental windows of sensitivity to social media exist. Higher SMU predicts lower life satisfaction later, with windows occurring at different ages for males and females. | Negative |
| [140](https://www.zotero.org/google-docs/?wif2mE) | Paquin et al (2022) | Longitudinal | Media use trajectories | Psychotic experiences | TV, video gaming, computer | Trajectories of higher video gaming and curved computer use were associated with higher levels of psychotic experiences at age 23. | Negative |
| [141](https://www.zotero.org/google-docs/?kHr6I5) | Parris et al (2022) | Cross-sectional survey | Social media rumination | Psychological distress (anxiety and depression) | Cyberbullying, social media posts | Social media rumination mediated the relationship between perceptions of bullying at school and feelings of distress. However, mediation was not supported when examining perceptions of cyberbullying frequency. | Negative |
| [142](https://www.zotero.org/google-docs/?MJYLrM) | Plackett, Sheringham & Dykxhoorn (2022) | Longitudinal cohort study | Hours spent on social media | Mental health (SDQ) | General social media use | In adjusted analyses, no association was found between time spent on social media at age 12-13 and poorer mental health at age 14-15. There was also no mediation effect found for self-esteem or social connectedness. | Neutral |
| [143](https://www.zotero.org/google-docs/?a5Zw6w) | Pop, Iorga & Iurcov (2022) | Cross-sectional survey | Social networking sites use | Body-esteem, self-esteem, loneliness, depression, stress | Socialization, entertainment, comparing profiles | The use of Snapchat was strongly positively correlated with self-esteem, and time spent on TikTok or Snapchat correlated with greater body esteem. However, almost half of the students experienced moderate to high levels of loneliness. | Mixed |
| [144](https://www.zotero.org/google-docs/?J7Li4O) | Samra, Warburton & Collins (2022) | Cross-sectional survey and lab experiment | Problematic Social Media Use (PSMU) and exposure to images | Depression and self-esteem | Upward and downward comparison images | Problematic social media use was associated with depression, low self-esteem, and comparing oneself negatively to others. Focusing on upward comparisons partially mediated the association between PSMU and depression. | Negative |
| [145](https://www.zotero.org/google-docs/?gTB5kv) | Svensson, Johnson & Olsson (2022) | Cross-sectional survey | Digital media activities (chatting, online sociability, self-presentation, gaming) | Internalizing symptoms | Chatting, online sociability, self-presentation, gaming | Social media use was positively associated with internalizing symptoms, but this effect was only significant for girls. Among specific activities, chatting and self-presentation were positively associated with internalizing symptoms. | Negative |
| [146](https://www.zotero.org/google-docs/?O8yR34) | Winstone et al (2022) | Longitudinal survey | Types of social media users | Self-harm, depression, anxiety, poor well-being | Messaging, sharing content (broadcasting), browsing | "Broadcasters" (adolescents who frequently share content along with messaging and browsing) had the poorest mental health outcomes a year later. Mental health and well-being were generally better among High and Moderate Communicators. | Mixed |
| [147](https://www.zotero.org/google-docs/?FaJ4rD) | Wong et al (2022) | Cross-sectional survey | Intensive and problematic social media use | Quality of relationships and connections | Online communication with others | Intensive use was associated with more positive social relationships with friends, especially among girls. In contrast, problematic use was consistently and negatively associated with strong relationships and connections. | Mixed |
| [148](https://www.zotero.org/google-docs/?XkJrKx) | Yurtdaş-Depboylu, Kaner & Özçakal (2022) | Cross-sectional study | Social media addiction, reading nutrition posts | Orthorexia nervosa (ON), eating attitudes, body image | Nutrition-related posts, idealized bodies | Social media addiction was associated with a higher risk of eating behavior disorders, orthorexia nervosa tendency, and lower body image. Adolescents who frequently read nutrition-related posts had an increased likelihood of eating behavior disorders and ON. | Negative |
| [149](https://www.zotero.org/google-docs/?yae7KV) | Zerhouni et al (2022) | Experimental study | Exposure to images of thin-ideal or average size women | State body satisfaction | Instagram images of women | Exposure to thin-ideal images was associated with greater decreases in body satisfaction only among participants who engaged in upwards appearance comparison. Emotion regulation difficulties and self-efficacy further moderated these effects. | Negative |
| [150](https://www.zotero.org/google-docs/?RaMgsj) | Anthony et al (2023) | Cross-sectional study | Online communication | Mental well-being | Communication with friends | Frequent online communication with real-life friends is associated with higher well-being, whereas frequent communication with virtual friends is associated with lower well-being. | Mixed |
| [151](https://www.zotero.org/google-docs/?28DC2J) | Armstrong-Carter et al (2023) | Longitudinal ecological momentary assessment (EMA) | Social media use to interact with peers | Social connectedness, social craving, and sensation seeking | Interacting with peers | For highly peer-susceptible adolescents, using social media to interact with peers was linked to feeling more socially connected, but also to craving more social connections and novel sensations. | Mixed |
| [152](https://www.zotero.org/google-docs/?nVpxFb) | Ayorech et al (2023) | Twin study and polygenic score methods | General media use, online victimisation, and problematic media use | Anxiety, depression, and prosocial behaviour | Negative media use and general media use | Negative media use correlated with poor mental health, while general media use correlated with prosocial behavior. These associations are largely explained by shared genetic influences. | Mixed |
| [153](https://www.zotero.org/google-docs/?yiFY4Q) | Burnell, Andrade & Hoyle (2023) | Longitudinal and EMA study | Digital technology use (entertainment, communication) | Self-control and perceived technological impairment | Entertainment and communication | Adolescents with poorer self-control perceived greater technological impairment and used more digital technology for entertainment. However, there were no within-person associations linking day-to-day self-control fluctuations with technology use. | Negative |
| [154](https://www.zotero.org/google-docs/?OKViC3) | Çiftci, Yıldız & Çiftci (2023) | Cross-sectional study | Social media addiction | Loneliness and social ostracism | Social media addiction | Social media addiction increases loneliness and social ostracism. Social ostracism mediates the effect of social media addiction on loneliness. | Negative |
| [155](https://www.zotero.org/google-docs/?OSrwZv) | Çimke & Yıldırım Gürkan (2023) | Cross-sectional study | Social media addiction and appearance-related social media consciousness | Body image perception | Filter use and sharing photos | Negative body image perception and social media addiction predict higher appearance-related social media consciousness. Being female, spending more time online, and using filters were strong predictors. | Negative |
| [156](https://www.zotero.org/google-docs/?HDYGBY) | Coyne et al (2023) | Cross-sectional study | Active/passive use, taking breaks, and curating feeds | Depression, emotional/conduct problems, and body image | Active use, taking breaks, curating feeds | Gender identity moderates the association between social media use and mental health. Active use and curating feeds were associated with better mental health for transgender/nonbinary (TGNB) youth, but taking breaks was associated with worse mental health. | Mixed |
| [157](https://www.zotero.org/google-docs/?pooBtd) | Irmer & Schmiedek (2023) | Daily diary study (EMA) | Social media use (Instagram, TikTok, YouTube) | Subjective well-being (self-worth, affect) and upward social comparisons | Upward social comparisons | Daily social media use was linked to lower positive and higher negative self-worth. This association was partially mediated by upward social comparisons. | Negative |
| [158](https://www.zotero.org/google-docs/?tmROkV) | Khan et al (2023) | Cross-sectional study | Screen time (TV, gaming, computer) | Life satisfaction | Active and passive screen time | Prolonged screen time (>2 hours/day) across all types is associated with low life satisfaction. Associations were slightly stronger for active screen time and for girls. | Negative |
| [159](https://www.zotero.org/google-docs/?KMEsx4) | Marciano & Viswanath (2023) | Cross-sectional study | Positive/negative social experiences and inspiration on social media | Flourishing (well-being) | Positive social media experiences, negative experiences, and inspiration | Positive social media experiences and social media inspiration were positively associated with flourishing, while negative social media experiences were inversely associated. | Mixed |
| [160](https://www.zotero.org/google-docs/?J5MqEV) | Mougharbel et al (2023) | Longitudinal study | Screen time (TV, video games, phone talking, internet) | Anxiety and depression symptoms | TV, video games, phone talking, internet surfing | Higher screen time was longitudinally associated with higher anxiety and depression symptoms one year later. Increases in screen use predicted greater emotional distress. | Negative |
| [161](https://www.zotero.org/google-docs/?shEf63) | Noel et al (2023) | Cross-sectional study | Smartphone addiction | Depression, anxiety, and suicide ideation | Smartphone addiction | Smartphone addiction is highly prevalent (34%) and strongly associated with greater odds of depression, anxiety, and suicide ideation. The link to depression was strongest in heterosexual cis-males. | Negative |
| [162](https://www.zotero.org/google-docs/?zkBOPa) | O'Reilly et al (2023) | Qualitative focus group | Social media use | Mental health and positive wellbeing | Peer-to-peer support and information seeking | Adolescents reported using social media to reduce stress, maintain social connections, find mental health information, and provide peer-to-peer support. | Positive |
| [163](https://www.zotero.org/google-docs/?nZ3Zll) | Orsolini, Longo & Volpe (2023) | Cross-sectional case-control | Problematic Internet Use (PIU) | Depression, anxiety, stress, loneliness, and boredom | Internet use | Boredom and loneliness double-mediate the association between depressive symptomatology and the onset/maintenance of Problematic Internet Use. | Negative |
| [164](https://www.zotero.org/google-docs/?zCifyP) | Panayiotou et al (2023) | Longitudinal panel network analysis | Time spent on social media | Mental health symptoms and life satisfaction | General social media use | Time spent on social media is one of the least influential factors for adolescent mental health, predicting only concentration problems in females across time. | Mixed |
| [165](https://www.zotero.org/google-docs/?KrQk9I) | Papapanou et al (2023) | Cross-sectional | Social media use (Social Media Disorder Scale) | Social appearance anxiety and loneliness | General social media use | Social appearance anxiety is positively correlated with loneliness and predicted by higher social media use, suggesting a complex vicious cycle. | Negative |
| [166](https://www.zotero.org/google-docs/?HJ1ATq) | Seekis & Lawrence (2023) | Experimental study | TikTok body neutrality content vs. thin-ideal content | Functionality appreciation, body satisfaction, and mood | Body neutrality vs. thin-ideal content | Brief exposure to body neutrality content on TikTok improved functionality appreciation, body satisfaction, and positive mood compared to thin-ideal content. | Positive |
| [167](https://www.zotero.org/google-docs/?S7Gkwg) | Skogen et al (2023) | Cross-sectional | Negative experiences on social media | Anxiety, depression, and well-being | Unwanted attention, exclusion | Commonly reported negative experiences on social media are consistently associated with poor mental health and lower well-being. | Negative |
| [168](https://www.zotero.org/google-docs/?kiNZw4) | Steinsbekk, Nesi & Wichstrøm (2023) | Longitudinal cohort study | Social media behaviors (self-oriented vs. other-oriented) | Symptoms of depression and anxiety | Posting, liking, and commenting | Within-person changes in self-oriented or other-oriented social media behaviors did not predict changes in symptoms of depression or anxiety two years later. | Mixed |
| [169](https://www.zotero.org/google-docs/?fIDVHy) | Szlyk et al (2023) | Mixed methods | Interacting with depression-related social media content | Suicidal ideation, suicide attempts, and NSSI | Depression-related social media content | Teens with suicidal ideation or NSSI histories were more likely to report disadvantages from interacting with depression-related social media. | Mixed |
| [170](https://www.zotero.org/google-docs/?xr9al8) | Tørmoen et al (2023) | Cross-sectional | Time spent on social media | Self-harm | General social media use | Spending more than 3 hours daily on social media is associated with a higher risk of self-harm, even after adjusting for confounders like depressive symptoms. | Negative |
| [171](https://www.zotero.org/google-docs/?LDzh6O) | Tuncturk et al (2023) | Cross-sectional clinical study | Internet Gaming Disorder (IGD) vs Problematic Internet Use (PIU) | Cyberbullying, aggression, and loneliness | Internet gaming and general internet use | Male adolescents clinically diagnosed with Internet Gaming Disorder had higher rates of psychiatric comorbidity, loneliness, and cyberbullying involvement than those with general PIU. | Negative |
| [172](https://www.zotero.org/google-docs/?ViWaak) | Zhu et al (2023) | Longitudinal study | Trajectories of screen time (TV, video games, surfing/chatting) | Depression, anxiety, suicidal ideation, self-injury, aggression, and substance use | TV, video games, surfing/chatting | Distinct screen time trajectories predict different adulthood outcomes; increasing chatting/surfing was linked to higher depression/anxiety, while gaming linked to suicidality. | Negative |
| [173](https://www.zotero.org/google-docs/?ttO7G8) | Angelini & Gini (2024) | Cross-sectional survey | Social media features (asynchronicity, cue absence, visualness) | Perceived online communication, e-motional processes | Disclosing emotions | Social media features like cue absence benefit online communication for socially anxious individuals, while asynchronicity enhances online emotional processes for them. | Mixed |
| [174](https://www.zotero.org/google-docs/?aBjVHd) | Atwal & Browne (2024) | Cross-sectional survey | Screen time (TV, video games, computer, smartphone) | Depression, anxiety, suicidal ideation | General screen time | Screen time and mental health follow an exposure-response curve where mental health peaks at around 12 hours/week and declines with higher use. | Negative |
| [175](https://www.zotero.org/google-docs/?ItCdUF) | Azagba, Ebling & Korkmaz (2024a) | Cross-sectional survey | Social media use frequency | Anxiety and depression symptoms | General social media use | Frequent social media use is associated with higher odds of e-cigarette use, mediated by anxiety and depression symptoms. | Negative |
| [176](https://www.zotero.org/google-docs/?C1xs0M) | Azagba, Ebling & Korkmaz (2024b) | Cross-sectional survey | Social media use frequency | Mental health conditions | General social media use | Frequent social media use is consistently associated with worse mental health conditions, especially among sexual minorities. | Negative |
| [177](https://www.zotero.org/google-docs/?mEmggs) | Barry et al (2024) | Cross-sectional survey | Social media engagement | Depression, anxiety, stress, sleep, loneliness, self-esteem, FOMO | Social media use during daily activities | Using social media during daily activities and perceiving it as interfering with life were linked to worse well-being. | Negative |
| [178](https://www.zotero.org/google-docs/?nX2cxR) | Basu et al (2024) | Cross-sectional survey | Facebook use | Suicidal ideation | Upward social comparisons | Upward social comparisons on Facebook predict suicidal ideation, but only among individuals with high levels of thwarted belongingness. | Negative |
| [179](https://www.zotero.org/google-docs/?fe0JKX) | Benedetto et al (2024) | Cross-sectional survey | Smartphone distraction | Internet addiction | Smartphone distraction, multitasking | Higher smartphone distraction is positively associated with Internet addiction, particularly for those with ADHD traits. | Negative |
| [180](https://www.zotero.org/google-docs/?dqY3g6) | Bitto Urbanova et al (2024) | Cross-sectional survey | Internet use | Emotional and behavioural problems | Excessive internet use | Adolescents with borderline or abnormal levels of emotional and behavioral problems are at a higher risk of excessive internet use. | Negative |
| [181](https://www.zotero.org/google-docs/?nBT00N) | Boniel-Nissim et al (2024) | Cross-national survey | Social media use and gaming | Problematic use, well-being | Continuous online contact | Problematic social media use has increased to 11%, with girls reporting higher problematic SMU and boys reporting higher problematic gaming. | Negative |
| [182](https://www.zotero.org/google-docs/?iUySVS) | Brimmel, Bijttebier & Eggermont (2024) | Cross-sectional survey | Social media content choices | Depressive symptoms | Mood-related affective content choices | Intentions to select sad social media content when feeling sad are associated with higher concurrent depressive symptoms. | Negative |
| [183](https://www.zotero.org/google-docs/?WgaLdE) | Carlberg Rindestig et al (2024) | Cross-sectional survey | Unwanted online sexual solicitation (UOSS) | Depression and anxiety | Unwanted online sexual solicitation | High occurrence of UOSS (48.6%) in the psychiatric sample, which significantly predicted anxiety and depressive symptoms. | Negative |
| [184](https://www.zotero.org/google-docs/?ek0dLd) | Carter et al (2024a) | Prospective cohort study | Problematic smartphone usage | Anxiety, depression, sleep | Problematic smartphone use | Increased problematic smartphone use over time is linearly associated with increased anxiety, depression, and inability to sleep. | Negative |
| [185](https://www.zotero.org/google-docs/?2EFHiP) | Carter et al (2024b) | Cross-sectional study | Problematic smartphone usage, daily screentime, and specific app usage | Anxiety, depression, and insomnia | Problematic smartphone use (addiction-like interactions), screentime, and social media app usage (Instagram, TikTok) | Problematic smartphone use was associated with increased anxiety, depression, and insomnia, independent of screentime. Screentime alone was not associated with anxiety or depression. | Negative |
| [186](https://www.zotero.org/google-docs/?MFyhVd) | Charmaraman et al (2024) | Cross-sectional survey | Social media, online communities | Loneliness | Exploring sexual identity, peer support | LGBTQ+ youth report higher loneliness, but gaining knowledge from LGBTQ+ sites and using TikTok/Instagram were associated with lower loneliness. | Mixed |
| [187](https://www.zotero.org/google-docs/?cZ2pJD) | Çınar Özbay et al (2024) | Cross-sectional survey | Internet addiction | Aggression, social anxiety | Internet addiction | Internet addiction significantly increases aggression, and this relationship is mediated by social anxiety. | Negative |
| [188](https://www.zotero.org/google-docs/?oUP7Br) | Davis & Goldfield (2024) | Randomized controlled trial (RCT) | Limiting smartphone social media use | Depression, anxiety, FoMO, sleep | General social media use restriction | Limiting social media use to 1 hour per day significantly reduced symptoms of depression, anxiety, and FoMO, and increased sleep duration. | Positive |
| [189](https://www.zotero.org/google-docs/?pNveCg) | Dyer et al (2024) | Longitudinal, person-centred | Social media use | Depression | Not specified | Social media use has varying effects on adolescent depression over time. While high use is often linked to increased depressive symptoms, a moderate amount can be protective, highlighting individual differences in susceptibility. | Mixed |
| [190](https://www.zotero.org/google-docs/?bzkFNp) | Eichenberg, Schneider & Rumpl (2024) | Cross-sectional | Social media use | Addiction, mental distress (depression, anxiety, somatization) | Addictive use of social media platforms | Social media addiction is associated with an ambivalent closed attachment style, neuroticism, and higher levels of depression, anxiety, and somatization. Secure attachment is linked to lower addiction and mental health problems. | Negative |
| [191](https://www.zotero.org/google-docs/?YHDMnI) | Ganson et al (2024) | Cross-sectional | Screen time, social media use | Weight-based bullying | Watching YouTube/TV shows/movies, playing games, social media use (Facebook, Instagram, TikTok, Twitter, Snapchat, Twitch) | Greater weekday screen time and use of social media platforms (especially Twitter and Twitch) are associated with a higher prevalence of weight-related bullying victimization among adolescents. These associations varied by country and sex. | Negative |
| [192](https://www.zotero.org/google-docs/?jvsFPz) | Garcia & Cooper (2024) | Cross-sectional | Social media use | Mental health, sleep | Social media use frequency, addiction, self-control failure, emotional investment | Social media addiction and self-control failure are positively associated with stress and poor sleep quality in Hispanic university emerging adults. Emotional investment in social media is positively linked to depression. | Negative |
| [193](https://www.zotero.org/google-docs/?3EFwVP) | Gingras et al (2024) | Longitudinal | Social media use | Internalizing symptoms (anxiety, depression) | Intensive and problematic social media use | Socially motivated social media use (e.g., to meet new people) predicts elevated internalizing symptoms. Avoidance and boredom motivations predict internalizing symptoms specifically for adolescents with low extraversion, while entertainment motivations are linked to lower symptoms. | Mixed |
| [194](https://www.zotero.org/google-docs/?2XUmZt) | Hamilton et al (2024) | Ecological momentary assessment | Social media use (screen time, experiences) | Suicidal ideation | Positive and negative socio-emotional experiences | Daily negative social media experiences increase the proximal risk for suicidal ideation, whereas positive experiences decrease this risk. Social media screen time itself is not associated with daily suicidal ideation. | Mixed |
| [195](https://www.zotero.org/google-docs/?nThCSK) | Herrmann et al (2024) | Cross-sectional | Social media use | Internalizing symptoms (suicidal ideation) | Experimenting with gender identity, online support networks, cyberbullying | For transgender and gender-diverse youth, positive online social experiences (like using support networks) were unexpectedly associated with more internalizing problems. Negative online experiences were linked to more externalizing problems but not internalizing problems. | Mixed |
| [196](https://www.zotero.org/google-docs/?v0xih0) | Idrees et al (2024) | Cross-sectional | Technology use (electronic devices) | Life stress, self-esteem | Not discussed | Moderate-to-high problem technology use is strongly associated with higher life stress and lower self-esteem among high school students. These symptoms are more common in females and linked to various sociodemographic factors. | Negative |
| [197](https://www.zotero.org/google-docs/?2x9JIa) | Kajastus et al (2024) | Cross-sectional | Internet use | Generalized and social anxiety | Excessive internet use | Generalized anxiety and social anxiety symptoms are closely related to excessive internet use. Generalized anxiety is also associated with decreased sleep duration, while social anxiety is linked to a lack of intense exercise. | Negative |
| [198](https://www.zotero.org/google-docs/?7efkiD) | Karabel, Onur & Şimşek (2024) | Cross-sectional | Internet use | Internet addiction, depression | Daily internet usage time | There is a significant positive correlation between daily internet usage time, internet addiction, and depression levels among adolescents. Severe internet addiction is associated with higher depression scores and suicidal ideation. | Negative |
| [199](https://www.zotero.org/google-docs/?fhXNee) | Klinger et al (2024) | Cross-sectional | Media use (smartphones, streaming services, social networks) | Depressive symptoms | Not discussed | Use of streaming services and social networks significantly predicts depressive symptoms in gender-diverse youth. Gender moderates the relationship between smartphone use and depressive symptoms, with the most negative effects seen in gender-diverse individuals. | Negative |
| [200](https://www.zotero.org/google-docs/?Ix7oPS) | Kosola, Mörö & Holopainen (2024) | Cross-sectional | Smartphone and social media use | Mental health, well-being (anxiety, body image, mood, loneliness) | Social media addiction | Social media addiction is common among adolescent girls and is associated with higher anxiety, poorer body image, lower mood, and greater tiredness and loneliness. Objectively measured smartphone use averaged nearly 6 hours daily. | Negative |
| [201](https://www.zotero.org/google-docs/?cG44lY) | Lahti et al (2024) | Cross-sectional | Social media threats | Depression, anxiety | Cyberbullying, sexual harassment, racism, misinformation, harmful challenges, appearance pressures | Daily and weekly exposure to social media threats (like misinformation and cyberbullying) is systematically associated with poor self-rated health, frequent depressive feelings, and anxiety symptoms. Problematic social media use and communicating with strangers increase exposure to these threats. | Negative |
| [202](https://www.zotero.org/google-docs/?Eb9sTW) | Lee et al (2024) | Longitudinal study | Frequent social media use (several times a day) | Internalizing mental health problems (feeling trapped, lonely, sad, depressed, sleep trouble, anxious, distressed) | Frequent social media use | Frequent social media use mediates the prospective association between experiencing internalizing mental health problems and using electronic nicotine delivery systems. | Negative |
| [203](https://www.zotero.org/google-docs/?cVIArq) | Livet et al (2024) | Longitudinal study | Screen time (social media use, television watching, video gaming) | Eating related symptoms (weight concerns, body type, bingeing behaviors) | Time spent on social media, TV, video games | Higher levels of social media use and TV watching were associated with higher eating related symptoms. Self-esteem mediated the association between screen time and eating related symptoms. | Negative |
| [204](https://www.zotero.org/google-docs/?yMAVlv) | Lowthian et al (2024) | Latent class analysis | Specific online behaviors (watching videos, playing games, schoolwork, posting pictures/videos, news, political discussions, speaking to family/friends/anyone online) | Mental well-being (emotional and conduct problems), social well-being, physical well-being, life satisfaction | Posting content, talking to internet-only friends, schoolwork, news intake, passive social media use. | Four classes identified: Avid users, Scholars, Midways, Passengers. Avid users (high posting and talking to strangers/internet friends) had the lowest well-being. | Mixed |
| [205](https://www.zotero.org/google-docs/?F68S2h) | Markey et al (2024) | Cross-sectional | Social media use | Body image | Appearance-focused vs. communication-focused use | Appearance-focused social media use was uniquely associated with negative body image, including higher body shame and lower body appreciation. Communication-focused use did not uniquely predict negative body image. | Negative |
| [206](https://www.zotero.org/google-docs/?WHFq47) | Martín-Cárdaba et al (2024) | Cross-sectional | Smartphone ownership | Well-being - adverse outcomes e.g. psychological distress, problematic usage patterns, or emulation of risky behaviors | Influencer content and engagement | Electronic device ownership was positively associated with psychological discomfort, problematic usage, and imitation of dangerous behaviors. This association was mediated by the consumption of influencer-generated content and the parasocial relationship with the influencer. | Negative |
| [207](https://www.zotero.org/google-docs/?5a3WKM) | Milson & Madigan (2024) | Cross-sectional + longitudinal | Instagram | Body satisfaction | Perfectionistic self-presentation (self-promotion, non-display of imperfection, non-disclosure of imperfection) | Perfectionistic self-presentation dimensions on Instagram were negatively associated with body satisfaction and positively associated with Instagram intrusion. Non-display of imperfection was the most important unique predictor of lower body satisfaction. | Negative |
| [208](https://www.zotero.org/google-docs/?LdGWp4) | Minich & Moreno (2024) | Ecological momentary assessment | Smartphone use | Mood disorders | General smartphone use | Adolescent smartphone use had a positive short-term, within-participant association with mood. Adolescents reported better moods when using their phones, and their moods improved during phone use. | Positive |
| [209](https://www.zotero.org/google-docs/?OcezCZ) | Murley et al (2024) | Cross-sectional | Social media use | Disordered eating | General social media use | Social media use was related to disordered eating both directly and indirectly through anxiety, but only related to food and alcohol disturbance through anxiety. These indirect effects were buffered by high social support. | Negative |
| [210](https://www.zotero.org/google-docs/?P5z6Du) | Pazarcikci (2024) | Cross-sectional | Technology use / screen time | Addiction | Not discussed | Low digital parenting awareness, high parental stress, male caregivers, low household income, and single-parent families increased the risk of technology addiction in young children. Using devices without parental controls also significantly increased addiction levels. | Negative |
| [211](https://www.zotero.org/google-docs/?Wjdphe) | Sarialioğlu & Oluç (2024) | Cross-sectional | Social media use | Stress | General social media use | A positive and significant relationship was found between social media addiction and perceived stress levels in adolescents. As social media addiction increased, perceived stress levels also increased. | Negative |
| [212](https://www.zotero.org/google-docs/?OFzDAT) | Sarman & Çiftci (2024) | Cross-sectional | Social media use | Appearance-related consciousness | Visual content, comparisons | A significant positive relationship existed between social media addiction and appearance-related social media consciousness. Increased time spent using social media was a predictor for both addiction and appearance-related consciousness. | Negative |
| [213](https://www.zotero.org/google-docs/?L7KJl6) | Schmidt-Persson et al (2024) | RCT | Screen media use | Mental health | General leisure-time screen media use | A short-term (2-week) reduction in leisure-time screen media use within families positively affected psychological symptoms in children and adolescents. It particularly mitigated internalizing behavioral issues and enhanced prosocial behavior. | Negative |
| [214](https://www.zotero.org/google-docs/?JII4Bo) | Seekis & Lawrence (2024) | Experimental | Tiktok use | Self-compassion | Body neutrality movement, thin ideal, art content | Young women exposed to TikTok body neutrality content reported significantly higher self-compassion than those who viewed thin ideal or art content. Brief exposure to body neutrality content may encourage healthier relationships with one's body. | Positive |
| [215](https://www.zotero.org/google-docs/?HZePHj) | Söyünmez et al (2024) | Descriptive and cross-sectional study | Social media use, social media addiction, influencer following | Objectified body consciousness, social appearance anxiety | Following influencers (makeup, exercise, nutrition/diet, funny posts) | Social media addiction, social appearance anxiety, and female gender increased objectified body consciousness. Following influencers who share exercise and makeup content increased self-objectification, while following nutrition/diet content decreased it. | Negative |
| [216](https://www.zotero.org/google-docs/?vg4cQo) | Szwedo et al (2024) | Longitudinal study | Social media posts from friends (social ties vs. faux pas posts) | Physical health (Interleukin-6/inflammation, sleep quality, physical functioning, BMI) | Receiving connection posts (social ties) vs. faux pas posts (deviating from norms) | Posts from friends characterized by social ties predicted better future physical health, such as better sleep and lower BMI. Conversely, socially inappropriate "faux pas" posts from friends predicted worse physical health outcomes, including higher inflammation and worse sleep. | Mixed |
| [217](https://www.zotero.org/google-docs/?NUzSD3) | Thai et al (2024) | Randomized controlled trial (RCT) | Reducing social media use (to 1 hr/day) | Appearance esteem and weight esteem | Overall smartphone SMU reduction | Reducing social media use to one hour per day for three weeks yielded significant improvements in both appearance and weight esteem. The control group with unrestricted access showed no significant changes in body esteem. | Positive |
| [218](https://www.zotero.org/google-docs/?y1kfEe) | Tibbs et al (2024) | Longitudinal panel study (Random Intercept Cross Lagged Path Models) | Interactive social media use (communication) vs. passive social media use (reading/viewing) | Internalizing difficulties (anxiety and depression) | Interactive communication (calling, messaging, chatting) vs. passive use (reading/viewing content) | There were no significant associations between passive social media use and internalizing difficulties at the within-person level over time. Interactive use was associated with decreases in internalizing difficulties at only one specific time point. | Neutral/Mixed |
| [219](https://www.zotero.org/google-docs/?Z9q2cY) | Trekels (2024) | Cross-sectional survey | Idealized vs. counter-idealized (body positive) social media content | Body dissatisfaction | Exposure to narrow appearance standards vs. body-positive content (visual and textual) | Idealized content was positively related to body dissatisfaction, while visual counter-idealized content was negatively related. Body dissatisfaction was lowest when adolescents had a balanced exposure to both types of content, but increased when idealized content outweighed counter-idealized content. | Mixed |
| [220](https://www.zotero.org/google-docs/?fvWlQD) | Tuck & Thompson (2024) | Experimental and cross-sectional study | Types of social media use (image-based, comparison-based, belief-based, consumption-based) | Trait and momentary affect (Positive Affect / Negative Affect) | Sharing opinions (belief), body comparison (comparison), monitoring likes (image), watching videos (consumption) | Comparison-based use was consistently associated with lower positive affect and higher negative affect at both trait and momentary levels. Image- and consumption-based use were associated with higher trait negative affect but made people feel better in the moment. | Mixed |
| [221](https://www.zotero.org/google-docs/?H1UY93) | Van de Casteele et al (2024) | Cross-sectional | Needs experiences: SMU satisfaction, SMU frustration, offline satisfaction, offline frustration | Vitality, Satisfaction with life, Anxiety and depression, Sleep quality | Not discussed | Need-experiences common to both offline and social media domains are robustly associated with mental health. Need satisfaction plays a beneficial role, while need frustration plays a harmful role. Offline need-experiences serve as a more crucial predictor of adolescents' mental health than need experiences on social media. | Mixed |
| [222](https://www.zotero.org/google-docs/?0Gv8VS) | Visier-Alfonso et al (2024) | Cross-sectional | Overall screen time, mobile phone use, social network use (Facebook, Twitter, Instagram, Snapchat, TikTok), and messaging application use (WhatsApp) | Depression, anxiety, and stress symptoms | Not discussed | Mobile phone and social network use were positively associated with symptoms of depression, anxiety, and stress. Socioeconomic status moderated these relationships, with stronger associations observed among adolescents with lower SES. Messaging application use did not show significant associations with mental health measures. | Negative |
| [223](https://www.zotero.org/google-docs/?0CrZlR) | Walsh et al (2024a) | Experimental | Restricting digital media (smartphone) use, restricting social media use | Life satisfaction, mindfulness, autonomy, competence, self-esteem, loneliness, stress, positive/negative emotions, depression, happiness | Not discussed | Restricting digital media (smartphone) use improved life satisfaction, mindfulness, autonomy, competence, and self-esteem, while reducing loneliness and stress. Restricting social media use provided relatively few benefits (increased mindfulness) and some potential costs (decreased positive emotions and increased negative emotions). | Mixed |
| [224](https://www.zotero.org/google-docs/?0ygMwe) | Walsh et al (2024b) | Cross-sectional | Online commercial sexual exploitation of children (CSEC), exchanging sexual talks, images, or activities for money, drugs, etc. using technology | Negative reactions involving embarrassment, anxiety, feeling afraid, ashamed, sad, angry, trouble concentrating, worse grades | Negative | 1.7% of respondents experienced online CSEC during childhood. Most reported negative emotional reactions such as embarrassment, anxiety, and fear. Sexual minority youth were more likely to exchange sexual talk, feel afraid, and fall behind in school compared to heterosexual youth. | Negative |
| [225](https://www.zotero.org/google-docs/?RjSebX) | Wingralek et al (2024) | Cross-sectional | Binge-watching (BW) of TV series | Psychological well-being, emotional and social loneliness | Binge watching | Decreased psychological well-being, increased emotional loneliness, and the desire to escape from problems increased the severity of binge-watching among university students. Escape, entertainment, and leisure motivations were also significant predictors of binge-watching. | Negative |
| [226](https://www.zotero.org/google-docs/?g79AM7) | Zsila et al (2024) | Cross-sectional | Twitch use (video game livestreaming platform), Problematic Use of Twitch | Symptoms of depression, anxiety, somatization, maladaptive daydreaming, need to belong | Video game livestreaming | Frequent Twitch use was not associated with psychological distress. However, problematic Twitch use was positively associated with psychological distress, maladaptive daydreaming, and a stronger need to belong. Only a small minority of users (1.3-4.8%) were at risk of problematic use. | Mixed |

**References**

[1.](https://www.zotero.org/google-docs/?yohmcP)	[Arias-de la Torre J, Puigdomenech E, García X, et al. Relationship Between Depression and the Use of Mobile Technologies and Social Media Among Adolescents: Umbrella Review. *J Med Internet Res*. 2020;22(8):e16388. doi:10.2196/16388](https://www.zotero.org/google-docs/?yohmcP)

[2.](https://www.zotero.org/google-docs/?yohmcP)	[Dienlin T, Johannes N. The impact of digital technology use on adolescent well-being. *Dialogues Clin Neurosci*. 2020;22(2):135-142. doi:10.31887/DCNS.2020.22.2/tdienlin](https://www.zotero.org/google-docs/?yohmcP)

[3.](https://www.zotero.org/google-docs/?yohmcP)	[Ghaemi SN. Digital depression: a new disease of the millennium? *Acta Psychiatr Scand*. 2020;141(4):356-361. doi:10.1111/acps.13151](https://www.zotero.org/google-docs/?yohmcP)

[4.](https://www.zotero.org/google-docs/?yohmcP)	[Ivie EJ, Pettitt A, Moses LJ, Allen NB. A meta-analysis of the association between adolescent social media use and depressive symptoms. *J Affect Disord*. 2020;275:165-174. doi:10.1016/j.jad.2020.06.014](https://www.zotero.org/google-docs/?yohmcP)

[5.](https://www.zotero.org/google-docs/?yohmcP)	[Lopez-Fernandez O, Kuss DJ. Preventing Harmful Internet Use-Related Addiction Problems in Europe: A Literature Review and Policy Options. *Int J Environ Res Public Health*. 2020;17(11):3797. doi:10.3390/ijerph17113797](https://www.zotero.org/google-docs/?yohmcP)

[6.](https://www.zotero.org/google-docs/?yohmcP)	[McCrory A, Best P, Maddock A. The relationship between highly visual social media and young people’s mental health: A scoping review. *Child Youth Serv Rev*. 2020;115:105053. doi:10.1016/j.childyouth.2020.105053](https://www.zotero.org/google-docs/?yohmcP)

[7.](https://www.zotero.org/google-docs/?yohmcP)	[Odgers CL, Jensen MR. Adolescent development and growing divides in the digital age. *Dialogues Clin Neurosci*. 2020;22(2):143-149. doi:10.31887/DCNS.2020.22.2/codgers](https://www.zotero.org/google-docs/?yohmcP)

[8.](https://www.zotero.org/google-docs/?yohmcP)	[Odgers CL, Jensen MR. Annual Research Review: Adolescent mental health in the digital age: facts, fears, and future directions. *J Child Psychol Psychiatry*. 2020;61(3):336-348. doi:10.1111/jcpp.13190](https://www.zotero.org/google-docs/?yohmcP)

[9.](https://www.zotero.org/google-docs/?yohmcP)	[Piteo EM, Ward K. Review: Social networking sites and associations with depressive and anxiety symptoms in children and adolescents – a systematic review. *Child Adolesc Ment Health*. 2020;25(4):201-216. doi:10.1111/camh.12373](https://www.zotero.org/google-docs/?yohmcP)

[10.](https://www.zotero.org/google-docs/?yohmcP)	[Pottie K, Ratnayake A, Ahmed R, Veronis L, Alghazali I. How refugee youth use social media: what does this mean for improving their health and welfare? *J Public Health Policy*. 2020;41(3):268-278. doi:10.1057/s41271-020-00231-4](https://www.zotero.org/google-docs/?yohmcP)

[11.](https://www.zotero.org/google-docs/?yohmcP)	[Rounsefell K, Gibson S, McLean S, et al. Social media, body image and food choices in healthy young adults: A mixed methods systematic review. *Nutr Diet J Dietit Assoc Aust*. 2020;77(1):19-40. doi:10.1111/1747-0080.12581](https://www.zotero.org/google-docs/?yohmcP)

[12.](https://www.zotero.org/google-docs/?yohmcP)	[Schønning V, Hjetland GJ, Aarø LE, Skogen JC. Social Media Use and Mental Health and Well-Being Among Adolescents – A Scoping Review. *Front Psychol*. 2020;11. doi:10.3389/fpsyg.2020.01949](https://www.zotero.org/google-docs/?yohmcP)

[13.](https://www.zotero.org/google-docs/?yohmcP)	[Vidal C, Vidal C, Vidal C, et al. Social media use and depression in adolescents: a scoping review. Published online 2020. doi:10.1080/09540261.2020.1720623](https://www.zotero.org/google-docs/?yohmcP)

[14.](https://www.zotero.org/google-docs/?yohmcP)	[R A, J H, S S, KK A. Interplay between social media use, sleep quality, and mental health in youth: A systematic review. *Sleep Med Rev*. 2021;56:101414. doi:10.1016/j.smrv.2020.101414](https://www.zotero.org/google-docs/?yohmcP)

[15.](https://www.zotero.org/google-docs/?yohmcP)	[Course-Choi J, Hammond L. Social Media Use and Adolescent Well-Being: A Narrative Review of Longitudinal Studies. *Cyberpsychology Behav Soc Netw*. 2021;24(4):223-236. doi:10.1089/cyber.2020.0020](https://www.zotero.org/google-docs/?yohmcP)

[16.](https://www.zotero.org/google-docs/?yohmcP)	[Ehrenreich SE, George MJ, Burnell K, Underwood MK. Importance of Digital Communication in Adolescents’ Development: Theoretical and Empirical Advancements in the Last Decade. *J Res Adolesc*. 2021;31(4):928-943. doi:10.1111/jora.12643](https://www.zotero.org/google-docs/?yohmcP)

[17.](https://www.zotero.org/google-docs/?yohmcP)	[Loleska S, Pop-Jordanova N. Is Smartphone Addiction in the Younger Population a Public Health Problem? *PRILOZI*. 2021;42(3):29-36. doi:10.2478/prilozi-2021-0032](https://www.zotero.org/google-docs/?yohmcP)

[18.](https://www.zotero.org/google-docs/?yohmcP)	[Stoilova M, Livingstone S, Khazbak R. Investigating Risks and Opportunities for Children in a Digital World: A Rapid Review of the Evidence on Children’s Internet Use and Outcomes. Published online May 2021. doi:10.18356/25211110-2020-03](https://www.zotero.org/google-docs/?yohmcP)

[19.](https://www.zotero.org/google-docs/?yohmcP)	[Tang S, Werner-Seidler A, Torok M, Mackinnon AJ, Christensen H. The relationship between screen time and mental health in young people: A systematic review of longitudinal studies. *Clin Psychol Rev*. 2021;86:102021. doi:10.1016/j.cpr.2021.102021](https://www.zotero.org/google-docs/?yohmcP)

[20.](https://www.zotero.org/google-docs/?yohmcP)	[Bozzola E, Spina G, Agostiniani R, et al. The Use of Social Media in Children and Adolescents: Scoping Review on the Potential Risks. *Int J Environ Res Public Health*. 2022;19(16):9960. doi:10.3390/ijerph19169960](https://www.zotero.org/google-docs/?yohmcP)

[21.](https://www.zotero.org/google-docs/?yohmcP)	[Choukas-Bradley S, Roberts SR, Maheux AJ, Nesi J. The Perfect Storm: A Developmental–Sociocultural Framework for the Role of Social Media in Adolescent Girls’ Body Image Concerns and Mental Health. *Clin Child Fam Psychol Rev*. 2022;25(4):681-701. doi:10.1007/s10567-022-00404-5](https://www.zotero.org/google-docs/?yohmcP)

[22.](https://www.zotero.org/google-docs/?yohmcP)	[Ferguson CJ, Kaye LK, Branley-Bell D, et al. Like this meta-analysis: Screen media and mental health. *Prof Psychol Res Pract*. 2022;53(2):205-214. doi:10.1037/pro0000426](https://www.zotero.org/google-docs/?yohmcP)

[23.](https://www.zotero.org/google-docs/?yohmcP)	[Haddock A, Ward N, Yu R, O’Dea N. Positive Effects of Digital Technology Use by Adolescents: A Scoping Review of the Literature. *Int J Environ Res Public Health*. 2022;19(21):21. doi:10.3390/ijerph192114009](https://www.zotero.org/google-docs/?yohmcP)

[24.](https://www.zotero.org/google-docs/?yohmcP)	[Kostyrka-Allchorne K, Stoilova M, Bourgaize J, Rahali M, Livingstone S, Sonuga-Barke E. Review: Digital experiences and their impact on the lives of adolescents with pre-existing anxiety, depression, eating and nonsuicidal self-injury conditions – a systematic review. *Child Adolesc Ment Health*. 2022;n/a(n/a). doi:10.1111/camh.12619](https://www.zotero.org/google-docs/?yohmcP)

[25.](https://www.zotero.org/google-docs/?yohmcP)	[Liu M, Kamper-DeMarco KE, Zhang J, Xiao J, Dong D, Xue P. Time Spent on Social Media and Risk of Depression in Adolescents: A Dose-Response Meta-Analysis. *Int J Environ Res Public Health*. 2022;19(9):5164. doi:10.3390/ijerph19095164](https://www.zotero.org/google-docs/?yohmcP)

[26.](https://www.zotero.org/google-docs/?yohmcP)	[Ricci RC, de Paulo ASC, de Freitas AKPB, et al. Impacts of technology on children’s health: a systematic review. *Rev Paul Pediatr*. 2022;41:e2020504. doi:10.1590/1984-0462/2023/41/2020504](https://www.zotero.org/google-docs/?yohmcP)

[27.](https://www.zotero.org/google-docs/?yohmcP)	[Shannon H, Bush K, Villeneuve PJ, Hellemans KG, Guimond S. Problematic Social Media Use in Adolescents and Young Adults: Systematic Review and Meta-analysis. *JMIR Ment Health*. 2022;9(4):e33450. doi:10.2196/33450](https://www.zotero.org/google-docs/?yohmcP)

[28.](https://www.zotero.org/google-docs/?yohmcP)	[Valkenburg PM, Meier A, Beyens I. Social media use and its impact on adolescent mental health: An umbrella review of the evidence. *Curr Opin Psychol*. 2022;44:58-68. doi:10.1016/j.copsyc.2021.08.017](https://www.zotero.org/google-docs/?yohmcP)

[29.](https://www.zotero.org/google-docs/?yohmcP)	[Vissenberg J, d’Haenens L, Livingstone S. Digital literacy and online resilience as facilitators of young people’s well-being? A systematic review. *Eur Psychol*. 2022;27(2):76-85. doi:10.1027/1016-9040/a000478](https://www.zotero.org/google-docs/?yohmcP)

[30.](https://www.zotero.org/google-docs/?yohmcP)	[Blanchard L, Conway-Moore K, Aguiar A, et al. Associations between social media, adolescent mental health, and diet: A systematic review. *Obes Rev Off J Int Assoc Study Obes*. 2023;24 Suppl 2:e13631. doi:10.1111/obr.13631](https://www.zotero.org/google-docs/?yohmcP)

[31.](https://www.zotero.org/google-docs/?yohmcP)	[Chochol MD, Gandhi K, Croarkin PE. Social Media and Anxiety in Youth. *Child Adolesc Psychiatr Clin N Am*. 2023;32(3):613-630. doi:10.1016/j.chc.2023.02.004](https://www.zotero.org/google-docs/?yohmcP)

[32.](https://www.zotero.org/google-docs/?yohmcP)	[Choukas-Bradley S, Kilic Z, Stout CD, Roberts SR. Perfect storms and double-edged swords: recent advances in research on adolescent social media use and mental health. *Adv Psychiatry Behav Health*. 2023;3(1):149-157.](https://www.zotero.org/google-docs/?yohmcP)

[33.](https://www.zotero.org/google-docs/?yohmcP)	[Dane A, Bhatia K. The social media diet: A scoping review to investigate the association between social media, body image and eating disorders amongst young people. *PLOS Glob Public Health*. 2023;3(3):e0001091. doi:10.1371/journal.pgph.0001091](https://www.zotero.org/google-docs/?yohmcP)

[34.](https://www.zotero.org/google-docs/?yohmcP)	[Ding H, Cao B, Sun Q. The association between problematic internet use and social anxiety within adolescents and young adults: a systematic review and meta-analysis. *Front Public Health*. 2023;11:1275723. doi:10.3389/fpubh.2023.1275723](https://www.zotero.org/google-docs/?yohmcP)

[35.](https://www.zotero.org/google-docs/?yohmcP)	[Harness J, Domoff SE, Rollings H. Social Media Use and Youth Mental Health: Intervention-Focused Future Directions. *Curr Psychiatry Rep*. 2023;25(12):865-871. doi:10.1007/s11920-023-01476-y](https://www.zotero.org/google-docs/?yohmcP)

[36.](https://www.zotero.org/google-docs/?yohmcP)	[Hilty DM, Stubbe D, McKean AJ, et al. A scoping review of social media in child, adolescents and young adults: research findings in depression, anxiety and other clinical challenges. *BJPsych Open*. 2023;9(5):e152. doi:10.1192/bjo.2023.523](https://www.zotero.org/google-docs/?yohmcP)

[37.](https://www.zotero.org/google-docs/?yohmcP)	[Jo S, Jang MY. Concept analysis of adolescent use of social media for emotional well-being. *Int J Nurs Pract*. 2023;29(1):e13116. doi:10.1111/ijn.13116](https://www.zotero.org/google-docs/?yohmcP)

[38.](https://www.zotero.org/google-docs/?yohmcP)	[Khalaf AM, Alubied AA, Khalaf AM, et al. The Impact of Social Media on the Mental Health of Adolescents and Young Adults: A Systematic Review. *Cureus*. 2023;15(8). doi:10.7759/cureus.42990](https://www.zotero.org/google-docs/?yohmcP)

[39.](https://www.zotero.org/google-docs/?yohmcP)	[Khetawat D, Steele RG. Examining the Association Between Digital Stress Components and Psychological Wellbeing: A Meta-Analysis. *Clin Child Fam Psychol Rev*. 2023;26(4):957-974. doi:10.1007/s10567-023-00440-9](https://www.zotero.org/google-docs/?yohmcP)

[40.](https://www.zotero.org/google-docs/?yohmcP)	[Messena M, Everri M. Unpacking the relation between children’s use of digital technologies and children’s well-being: A scoping review. *Clin Child Psychol Psychiatry*. 2023;28(1):161-198. doi:10.1177/13591045221127886](https://www.zotero.org/google-docs/?yohmcP)

[41.](https://www.zotero.org/google-docs/?yohmcP)	[Moss C, Wibberley C, Witham G. Assessing the impact of Instagram use and deliberate self‐harm in adolescents: A scoping review. *Int J Ment Health Nurs*. 2023;32(1):14-29. doi:10.1111/inm.13055](https://www.zotero.org/google-docs/?yohmcP)

[42.](https://www.zotero.org/google-docs/?yohmcP)	[Paulus MP, Zhao Y, Potenza MN, Aupperle RL, Bagot KS, Tapert SF. Screen media activity in youth: A critical review of mental health and neuroscience findings. *J Mood Anxiety Disord*. 2023;3. doi:10.1016/j.xjmad.2023.100018](https://www.zotero.org/google-docs/?yohmcP)

[43.](https://www.zotero.org/google-docs/?yohmcP)	[Popat A, Tarrant C. Exploring adolescents’ perspectives on social media and mental health and well-being – A qualitative literature review. *Clin Child Psychol Psychiatry*. 2023;28(1):323-337. doi:10.1177/13591045221092884](https://www.zotero.org/google-docs/?yohmcP)

[44.](https://www.zotero.org/google-docs/?yohmcP)	[Priftis N, Panagiotakos D. Screen Time and Its Health Consequences in Children and Adolescents. *Child Basel Switz*. 2023;10(10):1665. doi:10.3390/children10101665](https://www.zotero.org/google-docs/?yohmcP)

[45.](https://www.zotero.org/google-docs/?yohmcP)	[Santos RMS, Mendes CG, Sen Bressani GY, et al. The associations between screen time and mental health in adolescents: a systematic review. *BMC Psychol*. 2023;11(1):127. doi:10.1186/s40359-023-01166-7](https://www.zotero.org/google-docs/?yohmcP)

[46.](https://www.zotero.org/google-docs/?yohmcP)	[Susi K, Glover-Ford F, Stewart A, Knowles Bevis R, Hawton K. Research Review: Viewing self-harm images on the internet and social media platforms: systematic review of the impact and associated psychological mechanisms. *J Child Psychol Psychiatry*. 2023;64(8):1115-1139. doi:10.1111/jcpp.13754](https://www.zotero.org/google-docs/?yohmcP)

[47.](https://www.zotero.org/google-docs/?yohmcP)	[Ye XL, Zhang W, Zhao FF. Depression and internet addiction among adolescents:A meta-analysis. *Psychiatry Res*. 2023;326:115311. doi:10.1016/j.psychres.2023.115311](https://www.zotero.org/google-docs/?yohmcP)

[48.](https://www.zotero.org/google-docs/?yohmcP)	[Ahmed O, Walsh EI, Dawel A, Alateeq K, Espinoza Oyarce DA, Cherbuin N. Social media use, mental health and sleep: A systematic review with meta-analyses. *J Affect Disord*. 2024;367:701-712. doi:10.1016/j.jad.2024.08.193](https://www.zotero.org/google-docs/?yohmcP)

[49.](https://www.zotero.org/google-docs/?yohmcP)	[Dun-Campbell K, Hartwell G, Maani N, Tompson A, Schalkwyk MC van, Petticrew M. Commercial determinants of mental ill health: An umbrella review. *PLOS Glob Public Health*. 2024;4(8):e0003605. doi:10.1371/journal.pgph.0003605](https://www.zotero.org/google-docs/?yohmcP)

[50.](https://www.zotero.org/google-docs/?yohmcP)	[Engel E, Gell S, Heiss R, Karsay K. Social media influencers and adolescents’ health: A scoping review of the research field. *Soc Sci Med*. 2024;340:116387. doi:https://doi.org/10.1016/j.socscimed.2023.116387](https://www.zotero.org/google-docs/?yohmcP)

[51.](https://www.zotero.org/google-docs/?yohmcP)	[Ferguson CJ, Kaye LK, Branley-Bell D, Markey P. There is no evidence that time spent on social media is correlated with adolescent mental health problems: Findings from a meta-analysis. *Prof Psychol Res Pract*. Published online 2024:No Pagination Specified-No Pagination Specified. doi:10.1037/pro0000589](https://www.zotero.org/google-docs/?yohmcP)

[52.](https://www.zotero.org/google-docs/?yohmcP)	[Giancola N, Bentley KH, Torous J. Social Media and Suicidal Behavior in Youth: Slippery Slope or Simply Overstressed? *Curr Treat Options Psychiatry*. Published online July 1, 2024. doi:10.1007/s40501-024-00323-y](https://www.zotero.org/google-docs/?yohmcP)

[53.](https://www.zotero.org/google-docs/?yohmcP)	[Girela-Serrano BM, Spiers ADV, Ruotong L, Gangadia S, Toledano MB, Di Simplicio M. Impact of mobile phones and wireless devices use on children and adolescents’ mental health: a systematic review. *Eur Child Adolesc Psychiatry*. 2024;33(6):1621-1651. doi:10.1007/s00787-022-02012-8](https://www.zotero.org/google-docs/?yohmcP)

[54.](https://www.zotero.org/google-docs/?yohmcP)	[Hall JA. Ten Myths About the Effect of Social Media Use on Well-Being. *J Med Internet Res*. 2024;26:e59585. doi:10.2196/59585](https://www.zotero.org/google-docs/?yohmcP)

[55.](https://www.zotero.org/google-docs/?yohmcP)	[Jaycox LH, Murphy ER, Zehr JL, Pearson JL, Avenevoli S. Social Media and Suicide Risk in Youth. *JAMA Netw Open*. 2024;7(10):e2441499. doi:10.1001/jamanetworkopen.2024.41499](https://www.zotero.org/google-docs/?yohmcP)

[56.](https://www.zotero.org/google-docs/?yohmcP)	[Liu X, Liu J, Flores DD, McDonald CC. Family factors related to adolescent screen media use and mental health outcomes: A systematic review and recommendation for practices. *J Adolesc*. 2024;96(7):1401-1427. doi:10.1002/jad.12367](https://www.zotero.org/google-docs/?yohmcP)

[57.](https://www.zotero.org/google-docs/?yohmcP)	[McGorry PD, Mei C, Dalal N, et al. The Lancet Psychiatry Commission on youth mental health. *Lancet Psychiatry*. 2024;11(9):731-774. doi:10.1016/S2215-0366(24)00163-9](https://www.zotero.org/google-docs/?yohmcP)

[58.](https://www.zotero.org/google-docs/?yohmcP)	[National Academies of Sciences, Engineering, and Medicine. *Social Media and Adolescent Health*. (Galea S, Buckley GJ, Wojtowicz A, eds.). National Academies Press; 2024. doi:10.17226/27396](https://www.zotero.org/google-docs/?yohmcP)

[59.](https://www.zotero.org/google-docs/?yohmcP)	[Sanders T, Noetel M, Parker P, et al. An umbrella review of the benefits and risks associated with youths’ interactions with electronic screens. *Nat Hum Behav*. 2024;8(1):82-99. doi:10.1038/s41562-023-01712-8](https://www.zotero.org/google-docs/?yohmcP)

[60.](https://www.zotero.org/google-docs/?yohmcP)	[Sewall CJR, Parry DA. Social media and youth mental health: Simple narratives produce biased interpretations. *J Psychopathol Clin Sci*. 2024;133(7):507-514. doi:10.1037/abn0000950](https://www.zotero.org/google-docs/?yohmcP)

[61.](https://www.zotero.org/google-docs/?yohmcP)	[Weigle PE, Shafi RMA. Social Media and Youth Mental Health. *Curr Psychiatry Rep*. 2024;26(1):1-8. doi:10.1007/s11920-023-01478-w](https://www.zotero.org/google-docs/?yohmcP)

[62.](https://www.zotero.org/google-docs/?yohmcP)	[Yu DJ, Wing YK, Li TMH, Chan NY. The Impact of Social Media Use on Sleep and Mental Health in Youth: a Scoping Review. *Curr Psychiatry Rep*. 2024;26(3):104-119. doi:10.1007/s11920-024-01481-9](https://www.zotero.org/google-docs/?yohmcP)

[63.](https://www.zotero.org/google-docs/?yohmcP)	[Zhang Y, Li J, Zhang M, Ai B, Jia F. Bidirectional associations between loneliness and problematic internet use: A meta-analytic review of longitudinal studies. *Addict Behav*. 2024;150:107916. doi:10.1016/j.addbeh.2023.107916](https://www.zotero.org/google-docs/?yohmcP)

[64.](https://www.zotero.org/google-docs/?yohmcP)	[Barthorpe A, Winstone L, Mars B, Moran P. Is social media screen time really associated with poor adolescent mental health? A time use diary study. *J Affect Disord*. 2020;274:864-870. doi:10.1016/j.jad.2020.05.106](https://www.zotero.org/google-docs/?yohmcP)

[65.](https://www.zotero.org/google-docs/?yohmcP)	[Bennett BL, Whisenhunt BL, Hudson DL, et al. Examining the impact of social media on mood and body dissatisfaction using ecological momentary assessment. *J Am Coll Health*. 2020;68(5):502-508. doi:10.1080/07448481.2019.1583236](https://www.zotero.org/google-docs/?yohmcP)

[66.](https://www.zotero.org/google-docs/?yohmcP)	[Beyens I, Pouwels JL, Irene I. van Driel, van Driel II, Keijsers L, Valkenburg PM. The effect of social media on well-being differs from adolescent to adolescent. *Sci Rep*. 2020;10(1):10763-10763. doi:10.1038/s41598-020-67727-7](https://www.zotero.org/google-docs/?yohmcP)

[67.](https://www.zotero.org/google-docs/?yohmcP)	[Boer M, Van Den Eijnden RJJM, Boniel-Nissim M, et al. Adolescents’ Intense and Problematic Social Media Use and Their Well-Being in 29 Countries. *J Adolesc Health*. 2020;66(6):S89-S99. doi:10.1016/j.jadohealth.2020.02.014](https://www.zotero.org/google-docs/?yohmcP)

[68.](https://www.zotero.org/google-docs/?yohmcP)	[Boer M, Boer M, Stevens GWJM, et al. Social media use intensity, social media use problems, and mental health among adolescents: Investigating directionality and mediating processes. Published online 2020. doi:10.1016/j.chb.2020.106645](https://www.zotero.org/google-docs/?yohmcP)

[69.](https://www.zotero.org/google-docs/?yohmcP)	[Coyne SM, Coyne SM, Rogers AA, et al. Does time spent using social media impact mental health?: An eight year longitudinal study. Published online 2020. doi:10.1016/j.chb.2019.106160](https://www.zotero.org/google-docs/?yohmcP)

[70.](https://www.zotero.org/google-docs/?yohmcP)	[Craig W, Boniel-Nissim M, King N, et al. Social Media Use and Cyber-Bullying: A Cross-National Analysis of Young People in 42 Countries. *J Adolesc Health Off Publ Soc Adolesc Med*. 2020;66(6):S100-S108. doi:10.1016/j.jadohealth.2020.03.006](https://www.zotero.org/google-docs/?yohmcP)

[71.](https://www.zotero.org/google-docs/?yohmcP)	[Das-Friebel A, Lenneis A, Realo A, et al. Bedtime social media use, sleep, and affective wellbeing in young adults: an experience sampling study. *J Child Psychol Psychiatry*. 2020;61(10):1138-1149. doi:10.1111/jcpp.13326](https://www.zotero.org/google-docs/?yohmcP)

[72.](https://www.zotero.org/google-docs/?yohmcP)	[Escobar-Viera C, Shensa A, Hamm M, et al. “I Don’t Feel Like the Odd One”: Utilizing Content Analysis to Compare the Effects of Social Media Use on Well-Being Among Sexual Minority and Nonminority US Young Adults. *Am J Health Promot*. 2020;34(3):285-293. doi:10.1177/0890117119885517](https://www.zotero.org/google-docs/?yohmcP)

[73.](https://www.zotero.org/google-docs/?yohmcP)	[Fabris MA, Marengo D, Longobardi C, Settanni M. Investigating the links between fear of missing out, social media addiction, and emotional symptoms in adolescence: The role of stress associated with neglect and negative reactions on social media. *Addict Behav*. 2020;106:106364. doi:10.1016/j.addbeh.2020.106364](https://www.zotero.org/google-docs/?yohmcP)

[74.](https://www.zotero.org/google-docs/?yohmcP)	[Fardouly J, Magson NR, Rapee RM, Johnco CJ, Oar EL. The use of social media by Australian preadolescents and its links with mental health. *J Clin Psychol*. 2020;76(7):1304-1326. doi:10.1002/jclp.22936](https://www.zotero.org/google-docs/?yohmcP)

[75.](https://www.zotero.org/google-docs/?yohmcP)	[Gassó AM, Mueller-Johnson K, Montiel I. Sexting, Online Sexual Victimization, and Psychopathology Correlates by Sex: Depression, Anxiety, and Global Psychopathology. *Int J Environ Res Public Health*. 2020;17(3):1018. doi:10.3390/ijerph17031018](https://www.zotero.org/google-docs/?yohmcP)

[76.](https://www.zotero.org/google-docs/?yohmcP)	[George MJ, Jensen M, Russell MA, et al. Young Adolescents’ Digital Technology Use, Perceived Impairments, and Well-Being in a Representative Sample. *J Pediatr*. 2020;219:180-187. doi:10.1016/j.jpeds.2019.12.002](https://www.zotero.org/google-docs/?yohmcP)

[77.](https://www.zotero.org/google-docs/?yohmcP)	[Hawes T, Zimmer-Gembeck MJ, Campbell SM. Unique associations of social media use and online appearance preoccupation with depression, anxiety, and appearance rejection sensitivity. *Body Image*. 2020;33:66-76. doi:10.1016/j.bodyim.2020.02.010](https://www.zotero.org/google-docs/?yohmcP)

[78.](https://www.zotero.org/google-docs/?yohmcP)	[Kim S, Favotto L, Halladay J, Wang L, Boyle MH, Georgiades K. Differential associations between passive and active forms of screen time and adolescent mood and anxiety disorders. *Soc Psychiatry Psychiatr Epidemiol*. 2020;55(11):1469-1478. doi:10.1007/s00127-020-01833-9](https://www.zotero.org/google-docs/?yohmcP)

[79.](https://www.zotero.org/google-docs/?yohmcP)	[Kvardova N, Machackova H, Smahel D. The Direct and Indirect Effects of Online Social Support, Neuroticism, and Web Content Internalization on the Drive for Thinness among Women Visiting Health-Oriented Websites. *Int J Environ Res Public Health*. 2020;17(7):2416. doi:10.3390/ijerph17072416](https://www.zotero.org/google-docs/?yohmcP)

[80.](https://www.zotero.org/google-docs/?yohmcP)	[Mars B, Gunnell D, Biddle L, et al. Prospective associations between internet use and poor mental health: A population-based study. Abulseoud OA, ed. *PLOS ONE*. 2020;15(7):e0235889. doi:10.1371/journal.pone.0235889](https://www.zotero.org/google-docs/?yohmcP)

[81.](https://www.zotero.org/google-docs/?yohmcP)	[O’Reilly M. Social media and adolescent mental health: the good, the bad and the ugly. *J Ment Health*. 2020;29(2):200-206. doi:10.1080/09638237.2020.1714007](https://www.zotero.org/google-docs/?yohmcP)

[82.](https://www.zotero.org/google-docs/?yohmcP)	[Peris M, De La Barrera U, Schoeps K, Montoya-Castilla I. Psychological Risk Factors that Predict Social Networking and Internet Addiction in Adolescents. *Int J Environ Res Public Health*. 2020;17(12):4598. doi:10.3390/ijerph17124598](https://www.zotero.org/google-docs/?yohmcP)

[83.](https://www.zotero.org/google-docs/?yohmcP)	[Prichard I, Kavanagh E, Mulgrew KE, Lim MSC, Tiggemann M. The effect of Instagram #fitspiration images on young women’s mood, body image, and exercise behaviour. *Body Image*. 2020;33:1-6. doi:10.1016/j.bodyim.2020.02.002](https://www.zotero.org/google-docs/?yohmcP)

[84.](https://www.zotero.org/google-docs/?yohmcP)	[Puukko K, Hietajärvi L, Maksniemi E, Alho K, Salmela-Aro K. Social Media Use and Depressive Symptoms—A Longitudinal Study from Early to Late Adolescence. *Int J Environ Res Public Health*. 2020;17(16):5921. doi:10.3390/ijerph17165921](https://www.zotero.org/google-docs/?yohmcP)

[85.](https://www.zotero.org/google-docs/?yohmcP)	[Shensa A, Sidani JE, Escobar-Viera CG, Switzer GE, Primack BA, Choukas-Bradley S. Emotional support from social media and face-to-face relationships: Associations with depression risk among young adults. *J Affect Disord*. 2020;260:38-44. doi:10.1016/j.jad.2019.08.092](https://www.zotero.org/google-docs/?yohmcP)

[86.](https://www.zotero.org/google-docs/?yohmcP)	[Twenge JM, Twenge JM, Farley E, Farley E. Not all screen time is created equal: associations with mental health vary by activity and gender. Published online 2020. doi:10.1007/s00127-020-01906-9](https://www.zotero.org/google-docs/?yohmcP)

[87.](https://www.zotero.org/google-docs/?yohmcP)	[Twenge JM, Martin GN. Gender differences in associations between digital media use and psychological well‐being: Evidence from three large datasets. *J Adolesc*. 2020;79(1):91-102. doi:10.1016/j.adolescence.2019.12.018](https://www.zotero.org/google-docs/?yohmcP)

[88.](https://www.zotero.org/google-docs/?yohmcP)	[Twigg L, Duncan C, Weich S. Is social media use associated with children’s well‐being? Results from the UK Household Longitudinal Study. *J Adolesc*. 2020;80(1):73-83. doi:10.1016/j.adolescence.2020.02.002](https://www.zotero.org/google-docs/?yohmcP)

[89.](https://www.zotero.org/google-docs/?yohmcP)	[Beeres DT, Andersson F, Vossen HGM, Galanti MR. Social Media and Mental Health Among Early Adolescents in Sweden: A Longitudinal Study With 2-Year Follow-Up (KUPOL Study). *J Adolesc Health*. 2021;68(5):953-960. doi:10.1016/j.jadohealth.2020.07.042](https://www.zotero.org/google-docs/?yohmcP)

[90.](https://www.zotero.org/google-docs/?yohmcP)	[Belton S, Issartel J, Behan S, Goss H, Peers C. The Differential Impact of Screen Time on Children’s Wellbeing. *Int J Environ Res Public Health*. 2021;18(17):9143. doi:10.3390/ijerph18179143](https://www.zotero.org/google-docs/?yohmcP)

[91.](https://www.zotero.org/google-docs/?yohmcP)	[Beyens I, Pouwels JL, van Driel II, Keijsers L, Valkenburg PM. Social Media Use and Adolescents’ Well-Being: Developing a Typology of Person-Specific Effect Patterns. *Commun Res*. Published online December 2021:00936502211038196. doi:10.1177/00936502211038196](https://www.zotero.org/google-docs/?yohmcP)

[92.](https://www.zotero.org/google-docs/?yohmcP)	[Buda G, Lukoševičiūtė J, Šalčiūnaitė L, Šmigelskas K. Possible Effects of Social Media Use on Adolescent Health Behaviors and Perceptions. *Psychol Rep*. 2021;124(3):1031-1048. doi:10.1177/0033294120922481](https://www.zotero.org/google-docs/?yohmcP)

[93.](https://www.zotero.org/google-docs/?yohmcP)	[Charmaraman L, Richer AM, Liu C, Lynch AD, Moreno MA. Early Adolescent Social Media–Related Body Dissatisfaction: Associations with Depressive Symptoms, Social Anxiety, Peers, and Celebrities. *J Dev Behav Pediatr*. 2021;42(5):401-407. doi:10.1097/DBP.0000000000000911](https://www.zotero.org/google-docs/?yohmcP)

[94.](https://www.zotero.org/google-docs/?yohmcP)	[Coyne SM, Hurst JL, Dyer WJ, et al. Suicide Risk in Emerging Adulthood: Associations with Screen Time over 10 years. *J Youth Adolesc*. 2021;50(12):2324-2338. doi:10.1007/s10964-020-01389-6](https://www.zotero.org/google-docs/?yohmcP)

[95.](https://www.zotero.org/google-docs/?yohmcP)	[Dissing AS, Hulvej Rod N, Gerds TA, Lund R. Smartphone interactions and mental well-being in young adults: A longitudinal study based on objective high-resolution smartphone data. *Scand J Public Health*. 2021;49(3):325-332. doi:10.1177/1403494820920418](https://www.zotero.org/google-docs/?yohmcP)

[96.](https://www.zotero.org/google-docs/?yohmcP)	[Ferguson CJ. Links between screen use and depressive symptoms in adolescents over 16 years: Is there evidence for increased harm? *Dev Sci*. 2021;24(1):e13008. doi:10.1111/desc.13008](https://www.zotero.org/google-docs/?yohmcP)

[97.](https://www.zotero.org/google-docs/?yohmcP)	[Hamilton JL, Biernesser C, Moreno MA, et al. Social media use and prospective suicidal thoughts and behaviors among adolescents at high risk for suicide. *Suicide Life Threat Behav*. 2021;51(6):1203-1212. doi:10.1111/sltb.12801](https://www.zotero.org/google-docs/?yohmcP)

[98.](https://www.zotero.org/google-docs/?yohmcP)	[Hjetland GJ, Schønning V, Hella RT, Veseth M, Skogen JC. How do Norwegian adolescents experience the role of social media in relation to mental health and well-being: a qualitative study. *BMC Psychol*. 2021;9(1):78. doi:10.1186/s40359-021-00582-x](https://www.zotero.org/google-docs/?yohmcP)

[99.](https://www.zotero.org/google-docs/?yohmcP)	[Jarman HK, Marques MD, McLean SA, Slater A, Paxton SJ. Motivations for Social Media Use: Associations with Social Media Engagement and Body Satisfaction and Well-Being among Adolescents. *J Youth Adolesc*. 2021;50(12):2279-2293. doi:10.1007/s10964-020-01390-z](https://www.zotero.org/google-docs/?yohmcP)

[100.](https://www.zotero.org/google-docs/?yohmcP)	[Jarman HK, Marques MD, McLean SA, Slater A, Paxton SJ. Social media, body satisfaction and well-being among adolescents: A mediation model of appearance-ideal internalization and comparison. *Body Image*. 2021;36:139-148. doi:10.1016/j.bodyim.2020.11.005](https://www.zotero.org/google-docs/?yohmcP)

[101.](https://www.zotero.org/google-docs/?yohmcP)	[Khan A, Lee EY, Rosenbaum S, Khan SR, Tremblay MS. Dose-dependent and joint associations between screen time, physical activity, and mental wellbeing in adolescents: an international observational study. *Lancet Child Adolesc Health*. 2021;5(10):729-738. doi:10.1016/S2352-4642(21)00200-5](https://www.zotero.org/google-docs/?yohmcP)

[102.](https://www.zotero.org/google-docs/?yohmcP)	[Kleppang AL, Steigen AM, Ma L, Søberg Finbråten H, Hagquist C. Electronic media use and symptoms of depression among adolescents in Norway. Van Amelsvoort T, ed. *PLOS ONE*. 2021;16(7):e0254197. doi:10.1371/journal.pone.0254197](https://www.zotero.org/google-docs/?yohmcP)

[103.](https://www.zotero.org/google-docs/?yohmcP)	[Kohler MT, Turner IN, Webster GD. Social comparison and state–trait dynamics: Viewing image-conscious Instagram accounts affects college students’ mood and anxiety. *Psychol Pop Media*. 2021;10(3):340-349. doi:10.1037/ppm0000310](https://www.zotero.org/google-docs/?yohmcP)

[104.](https://www.zotero.org/google-docs/?yohmcP)	[Kürtüncü M, Ayyıldız TK, Kurt A. An examination of smartphone addiction and loneliness among high school students according to various variables: A sample from Turkey. *Perspect Psychiatr Care*. 2021;57(2):941-947. doi:10.1111/ppc.12639](https://www.zotero.org/google-docs/?yohmcP)

[105.](https://www.zotero.org/google-docs/?yohmcP)	[Kvardova N, Smahel D, Machackova H, Subrahmanyam K. Who Is Exposed to Harmful Online Content? The Role of Risk and Protective Factors Among Czech, Finnish, and Spanish Adolescents. *J Youth Adolesc*. 2021;50(12):2294-2310. doi:10.1007/s10964-021-01422-2](https://www.zotero.org/google-docs/?yohmcP)

[106.](https://www.zotero.org/google-docs/?yohmcP)	[MacDonald KB, Schermer JA. Loneliness unlocked: Associations with smartphone use and personality. *Acta Psychol (Amst)*. 2021;221:103454. doi:10.1016/j.actpsy.2021.103454](https://www.zotero.org/google-docs/?yohmcP)

[107.](https://www.zotero.org/google-docs/?yohmcP)	[Marengo N, Borraccino A, Charrier L, et al. Cyberbullying and problematic social media use: an insight into the positive role of social support in adolescents—data from the Health Behaviour in School-aged Children study in Italy. *Public Health*. 2021;199:46-50. doi:10.1016/j.puhe.2021.08.010](https://www.zotero.org/google-docs/?yohmcP)

[108.](https://www.zotero.org/google-docs/?yohmcP)	[McComb SE, Mills JS. Young women’s body image following upwards comparison to Instagram models: The role of physical appearance perfectionism and cognitive emotion regulation. *Body Image*. 2021;38:49-62. doi:10.1016/j.bodyim.2021.03.012](https://www.zotero.org/google-docs/?yohmcP)

[109.](https://www.zotero.org/google-docs/?yohmcP)	[Mundy LK, Canterford L, Moreno‐Betancur M, et al. Social networking and symptoms of depression and anxiety in early adolescence. *Depress Anxiety*. 2021;38(5):563-570. doi:10.1002/da.23117](https://www.zotero.org/google-docs/?yohmcP)

[110.](https://www.zotero.org/google-docs/?yohmcP)	[Paakkari L, Tynjälä J, Lahti H, Ojala K, Lyyra N. Problematic Social Media Use and Health among Adolescents. *Int J Environ Res Public Health*. 2021;18(4):1885. doi:10.3390/ijerph18041885](https://www.zotero.org/google-docs/?yohmcP)

[111.](https://www.zotero.org/google-docs/?yohmcP)	[Parsons CA, Alden LE, Biesanz JC. Influencing emotion: Social anxiety and comparisons on Instagram. *Emotion*. 2021;21(7):1427-1437. doi:10.1037/emo0001044](https://www.zotero.org/google-docs/?yohmcP)

[112.](https://www.zotero.org/google-docs/?yohmcP)	[Paulich KN, Ross JM, Lessem JM, Hewitt JK. Screen time and early adolescent mental health, academic, and social outcomes in 9- and 10- year old children: Utilizing the Adolescent Brain Cognitive Development ^SM^ (ABCD) Study. Kabir E, ed. *PLOS ONE*. 2021;16(9):e0256591. doi:10.1371/journal.pone.0256591](https://www.zotero.org/google-docs/?yohmcP)

[113.](https://www.zotero.org/google-docs/?yohmcP)	[Petropoulos Petalas D, Konijn EA, Johnson BK, et al. Plurality in the measurement of social media use and mental health: An exploratory study among adolescents and young adults. *Soc Media Soc*. 2021;7(3):20563051211035353.](https://www.zotero.org/google-docs/?yohmcP)

[114.](https://www.zotero.org/google-docs/?yohmcP)	[Pouwels JL, Valkenburg PM, Beyens I, van Driel II, Keijsers L. Social media use and friendship closeness in adolescents’ daily lives: An experience sampling study. *Dev Psychol*. 2021;57(2):309-323. doi:10.1037/dev0001148](https://www.zotero.org/google-docs/?yohmcP)

[115.](https://www.zotero.org/google-docs/?yohmcP)	[Shafi RM, Nakonezny PA, Miller KA, et al. Altered markers of stress in depressed adolescents after acute social media use. *J Psychiatr Res*. 2021;136:149-156. doi:10.1016/j.jpsychires.2021.01.055](https://www.zotero.org/google-docs/?yohmcP)

[116.](https://www.zotero.org/google-docs/?yohmcP)	[Skogen JC, Hjetland GJ, Bøe T, Hella RT, Knudsen AK. Through the Looking Glass of Social Media. Focus on Self-Presentation and Association with Mental Health and Quality of Life. A Cross-Sectional Survey-Based Study. *Int J Environ Res Public Health*. 2021;18(6):3319. doi:10.3390/ijerph18063319](https://www.zotero.org/google-docs/?yohmcP)

[117.](https://www.zotero.org/google-docs/?yohmcP)	[Tamarit A, Schoeps K, Peris-Hernández M, Montoya-Castilla I. The Impact of Adolescent Internet Addiction on Sexual Online Victimization: The Mediating Effects of Sexting and Body Self-Esteem. *Int J Environ Res Public Health*. 2021;18(8):4226. doi:10.3390/ijerph18084226](https://www.zotero.org/google-docs/?yohmcP)

[118.](https://www.zotero.org/google-docs/?yohmcP)	[Trumello C, Vismara L, Sechi C, Ricciardi P, Marino V, Babore A. Internet Addiction: The Role of Parental Care and Mental Health in Adolescence. *Int J Environ Res Public Health*. 2021;18(24):12876. doi:10.3390/ijerph182412876](https://www.zotero.org/google-docs/?yohmcP)

[119.](https://www.zotero.org/google-docs/?yohmcP)	[Vuorre M, Orben A, Przybylski AK. There Is No Evidence That Associations Between Adolescents’ Digital Technology Engagement and Mental Health Problems Have Increased. *Clin Psychol Sci*. 2021;9(5):823-835. doi:10.1177/2167702621994549](https://www.zotero.org/google-docs/?yohmcP)

[120.](https://www.zotero.org/google-docs/?yohmcP)	[Azhari A, Toms Z, Pavlopoulou G, Esposito G, Dimitriou D. Social media use in female adolescents: Associations with anxiety, loneliness, and sleep disturbances. *Acta Psychol (Amst)*. 2022;229:103706. doi:10.1016/j.actpsy.2022.103706](https://www.zotero.org/google-docs/?yohmcP)

[121.](https://www.zotero.org/google-docs/?yohmcP)	[Boer M, Stevens GWJM, Finkenauer C, van den Eijnden RJJM. The complex association between social media use intensity and adolescent wellbeing: A longitudinal investigation of five factors that may affect the association. *Comput Hum Behav*. 2022;128:107084. doi:10.1016/j.chb.2021.107084](https://www.zotero.org/google-docs/?yohmcP)

[122.](https://www.zotero.org/google-docs/?yohmcP)	[Bohnert M, Gracia P. Digital Engagement, Social Inequalities and Adolescent Well-Being. *SocArXiv*. Preprint posted online July 28, 2022. doi:10.31235/osf.io/ks3tj](https://www.zotero.org/google-docs/?yohmcP)

[123.](https://www.zotero.org/google-docs/?yohmcP)	[Boniel-Nissim M, van den Eijnden RJJM, Furstova J, et al. International perspectives on social media use among adolescents: Implications for mental and social well-being and substance use. *Comput Hum Behav*. 2022;129:107144. doi:10.1016/j.chb.2021.107144](https://www.zotero.org/google-docs/?yohmcP)

[124.](https://www.zotero.org/google-docs/?yohmcP)	[Castrén S, Mustonen T, Hylkilä K, Männikkö N, Kääriäinen M, Raitasalo K. Risk Factors for Excessive Social Media Use Differ from Those of Gambling and Gaming in Finnish Youth. *Int J Environ Res Public Health*. 2022;19(4):2406. doi:10.3390/ijerph19042406](https://www.zotero.org/google-docs/?yohmcP)

[125.](https://www.zotero.org/google-docs/?yohmcP)	[Dissing AS, Andersen TO, Jensen AK, Lund R, Rod NH. Nighttime smartphone use and changes in mental health and wellbeing among young adults: a longitudinal study based on high-resolution tracking data. *Sci Rep*. 2022;12(1):8013. doi:10.1038/s41598-022-10116-z](https://www.zotero.org/google-docs/?yohmcP)

[126.](https://www.zotero.org/google-docs/?yohmcP)	[Erdem E, Sezer Efe Y. The smartphone addiction, peer relationships and loneliness in adolescents. *L’Encéphale*. 2022;48(5):490-495. doi:10.1016/j.encep.2021.06.009](https://www.zotero.org/google-docs/?yohmcP)

[127.](https://www.zotero.org/google-docs/?yohmcP)	[Gámez-Guadix M, Mateos E, Wachs S, Blanco M. Self-Harm on the Internet Among Adolescents: Prevalence and Association with Depression, Anxiety, Family Cohesion, and Social Resources. *Psicothema*. 2022;2(34):233-239. doi:10.7334/psicothema2021.328](https://www.zotero.org/google-docs/?yohmcP)

[128.](https://www.zotero.org/google-docs/?yohmcP)	[Garcia RL, Bingham S, Liu S. The effects of daily Instagram use on state self-objectification, well-being, and mood for young women. *Psychol Pop Media*. 2022;11(4):423-434. doi:10.1037/ppm0000350](https://www.zotero.org/google-docs/?yohmcP)

[129.](https://www.zotero.org/google-docs/?yohmcP)	[Günaydın N, Kaşko Arıcı Y, Kutlu FY, Yancar Demir E. The relationship between problematic Internet use in adolescents and emotion regulation difficulty and family Internet attitude. *J Community Psychol*. 2022;50(2):1135-1154. doi:10.1002/jcop.22708](https://www.zotero.org/google-docs/?yohmcP)

[130.](https://www.zotero.org/google-docs/?yohmcP)	[Lee S, Lohrmann DK, Luo J, Chow A. Frequent Social Media Use and Its Prospective Association With Mental Health Problems in a Representative Panel Sample of US Adolescents. *J Adolesc Health*. 2022;70(5):796-803. doi:10.1016/j.jadohealth.2021.11.029](https://www.zotero.org/google-docs/?yohmcP)

[131.](https://www.zotero.org/google-docs/?yohmcP)	[Mann RB, Blumberg F. Adolescents and social media: The effects of frequency of use, self-presentation, social comparison, and self esteem on possible self imagery. *Acta Psychol (Amst)*. 2022;228:103629. doi:10.1016/j.actpsy.2022.103629](https://www.zotero.org/google-docs/?yohmcP)

[132.](https://www.zotero.org/google-docs/?yohmcP)	[Marciano L, Driver CC, Schulz PJ, Camerini AL. Dynamics of adolescents’ smartphone use and well-being are positive but ephemeral. *Sci Rep*. 2022;12(1):1316. doi:10.1038/s41598-022-05291-y](https://www.zotero.org/google-docs/?yohmcP)

[133.](https://www.zotero.org/google-docs/?yohmcP)	[Marciano L, Schulz PJ, Camerini AL. How do depression, duration of internet use and social connection in adolescence influence each other over time? An extension of the RI-CLPM including contextual factors. *Comput Hum Behav*. 2022;136:107390. doi:10.1016/j.chb.2022.107390](https://www.zotero.org/google-docs/?yohmcP)

[134.](https://www.zotero.org/google-docs/?yohmcP)	[McArthur BA, Browne D, Racine N, Tough S, Madigan S. Screen Time as a Mechanism Through Which Cumulative Risk is Related to Child Socioemotional and Developmental Outcomes in Early Childhood. *Res Child Adolesc Psychopathol*. 2022;50(6):709-720. doi:10.1007/s10802-021-00895-w](https://www.zotero.org/google-docs/?yohmcP)

[135.](https://www.zotero.org/google-docs/?yohmcP)	[McCrory A, Best P, Maddock A. ‘It’s just one big vicious circle’: young people’s experiences of highly visual social media and their mental health. *Health Educ Res*. 2022;37(3):167-184. doi:10.1093/her/cyac010](https://www.zotero.org/google-docs/?yohmcP)

[136.](https://www.zotero.org/google-docs/?yohmcP)	[Mehtälä S, Salo M, Tikka S, Pirkkalainen H. Exploring early adolescents’ stressful IT use experiences. *Behav Inf Technol*. Published online August 9, 2022:1-15. doi:10.1080/0144929X.2022.2109991](https://www.zotero.org/google-docs/?yohmcP)

[137.](https://www.zotero.org/google-docs/?yohmcP)	[Miljeteig K, von Soest T. An experience sampling study on the association between social media use and self-esteem. *J Media Psychol Theor Methods Appl*. 2022;34(6):373-382. doi:10.1027/1864-1105/a000333](https://www.zotero.org/google-docs/?yohmcP)

[138.](https://www.zotero.org/google-docs/?yohmcP)	[Moreton L, Greenfield S. University students’ views on the impact of Instagram on mental wellbeing: a qualitative study. *BMC Psychol*. 2022;10(1):45. doi:10.1186/s40359-022-00743-6](https://www.zotero.org/google-docs/?yohmcP)

[139.](https://www.zotero.org/google-docs/?yohmcP)	[Orben A, Przybylski AK, Blakemore SJ, Kievit RA. Windows of developmental sensitivity to social media. *Nat Commun*. 2022;13(1):1649. doi:10.1038/s41467-022-29296-3](https://www.zotero.org/google-docs/?yohmcP)

[140.](https://www.zotero.org/google-docs/?yohmcP)	[Paquin V, Ferrari M, Rej S, et al. Trajectories of Adolescent Media Use and Their Associations With Psychotic Experiences. *JAMA Psychiatry*. 2024;81(7):708. doi:10.1001/jamapsychiatry.2024.0384](https://www.zotero.org/google-docs/?yohmcP)

[141.](https://www.zotero.org/google-docs/?yohmcP)	[Parris L, Lannin DG, Hynes K, Yazedjian A. Exploring Social Media Rumination: Associations With Bullying, Cyberbullying, and Distress. *J Interpers Violence*. 2022;37(5-6). doi:10.1177/0886260520946826](https://www.zotero.org/google-docs/?yohmcP)

[142.](https://www.zotero.org/google-docs/?yohmcP)	[Plackett R, Sheringham J, Dykxhoorn J. The longitudinal effect of social media use on adolescent mental health in the UK: findings from the UK Longitudinal Household Study. *The Lancet*. 2022;400:S71. doi:10.1016/S0140-6736(22)02281-4](https://www.zotero.org/google-docs/?yohmcP)

[143.](https://www.zotero.org/google-docs/?yohmcP)	[Pop LM, Iorga M, Iurcov R. Body-Esteem, Self-Esteem and Loneliness among Social Media Young Users. *Int J Environ Res Public Health*. 2022;19(9):5064. doi:10.3390/ijerph19095064](https://www.zotero.org/google-docs/?yohmcP)

[144.](https://www.zotero.org/google-docs/?yohmcP)	[Samra A, Warburton WA, Collins AM. Social comparisons: A potential mechanism linking problematic social media use with depression. *J Behav Addict*. 2022;11(2):607-614. doi:10.1556/2006.2022.00023](https://www.zotero.org/google-docs/?yohmcP)

[145.](https://www.zotero.org/google-docs/?yohmcP)	[Svensson R, Johnson B, Olsson A. Does gender matter? The association between different digital media activities and adolescent well-being. *BMC Public Health*. 2022;22(1):273. doi:10.1186/s12889-022-12670-7](https://www.zotero.org/google-docs/?yohmcP)

[146.](https://www.zotero.org/google-docs/?yohmcP)	[Winstone L, Mars B, Haworth CM, Heron J, Kidger J. Adolescent social media user types and their mental health and well‐being: Results from a longitudinal survey of 13–14‐year‐olds in the United Kingdom. *JCPP Adv*. 2022;2(2):e12071.](https://www.zotero.org/google-docs/?yohmcP)

[147.](https://www.zotero.org/google-docs/?yohmcP)	[Wong SL, King N, Gariépy G, et al. Adolescent social media use and its association with relationships and connections: Canadian Health Behaviour in School-aged Children, 2017/2018. *Health Rep*. 2022;33(12):14-23. doi:10.25318/82-003-x202201200002-eng](https://www.zotero.org/google-docs/?yohmcP)

[148.](https://www.zotero.org/google-docs/?yohmcP)	[Yurtdaş-Depboylu G, Kaner G, Özçakal S. The association between social media addiction and orthorexia nervosa, eating attitudes, and body image among adolescents. *Eat Weight Disord - Stud Anorex Bulim Obes*. 2022;27(8):3725-3735. doi:10.1007/s40519-022-01521-4](https://www.zotero.org/google-docs/?yohmcP)

[149.](https://www.zotero.org/google-docs/?yohmcP)	[Zerhouni O, Flaudias V, Barré M, Rodgers RF. The effects of exposure to social media images of thin and average size women on body satisfaction among young women: Emotion regulation and self-efficacy as modulating factors. *Body Image*. 2022;43:292-300. doi:10.1016/j.bodyim.2022.09.011](https://www.zotero.org/google-docs/?yohmcP)

[150.](https://www.zotero.org/google-docs/?yohmcP)	[Anthony R, Young H, Hewitt G, et al. Young people’s online communication and its association with mental well‐being: results from the 2019 student health and well‐being survey. *Child Adolesc Ment Health*. 2023;28(1):4-11. doi:10.1111/camh.12610](https://www.zotero.org/google-docs/?yohmcP)

[151.](https://www.zotero.org/google-docs/?yohmcP)	[Armstrong-Carter E, Garrett SL, Nick EA, Prinstein MJ, Telzer EH. Momentary links between adolescents’ social media use and social experiences and motivations: Individual differences by peer susceptibility. *Dev Psychol*. 2023;59(4):707-719. doi:10.1037/dev0001503](https://www.zotero.org/google-docs/?yohmcP)

[152.](https://www.zotero.org/google-docs/?yohmcP)	[Ayorech Z, Baldwin JR, Pingault JB, Rimfeld K, Plomin R. Gene-environment correlations and genetic confounding underlying the association between media use and mental health. *Sci Rep*. 2023;13(1):1030. doi:10.1038/s41598-022-25374-0](https://www.zotero.org/google-docs/?yohmcP)

[153.](https://www.zotero.org/google-docs/?yohmcP)	[Burnell K, Andrade FC, Hoyle RH. Longitudinal and daily associations between adolescent self-control and digital technology use. *Dev Psychol*. 2023;59(4):720-732. doi:10.1037/dev0001444](https://www.zotero.org/google-docs/?yohmcP)

[154.](https://www.zotero.org/google-docs/?yohmcP)	[Çiftci N, Yıldız M, Çiftci K. The mediating role of social ostracism in the effect of social media addiction on loneliness in adolescents. *J Pediatr Nurs*. 2023;73:177-183. doi:10.1016/j.pedn.2023.09.005](https://www.zotero.org/google-docs/?yohmcP)

[155.](https://www.zotero.org/google-docs/?yohmcP)	[Çimke S, Yıldırım Gürkan D. Factors affecting body image perception, social media addiction, and social media consciousness regarding physical appearance in adolescents. *J Pediatr Nurs*. 2023;73:e197-e203. doi:10.1016/j.pedn.2023.09.010](https://www.zotero.org/google-docs/?yohmcP)

[156.](https://www.zotero.org/google-docs/?yohmcP)	[Coyne SM, Weinstein E, Sheppard JA, et al. Analysis of Social Media Use, Mental Health, and Gender Identity Among US Youths. *JAMA Netw Open*. 2023;6(7):e2324389. doi:10.1001/jamanetworkopen.2023.24389](https://www.zotero.org/google-docs/?yohmcP)

[157.](https://www.zotero.org/google-docs/?yohmcP)	[Irmer A, Schmiedek F. Associations between youth’s daily social media use and well-being are mediated by upward comparisons. *Commun Psychol*. 2023;1(1):12. doi:10.1038/s44271-023-00013-0](https://www.zotero.org/google-docs/?yohmcP)

[158.](https://www.zotero.org/google-docs/?yohmcP)	[Khan A, Moni MA, Khan SR, Burton NW. Different types of screen time are associated with low life satisfaction in adolescents across 37 European and North American countries. *Scand J Public Health*. 2023;51(6):918-925. doi:10.1177/14034948221082459](https://www.zotero.org/google-docs/?yohmcP)

[159.](https://www.zotero.org/google-docs/?yohmcP)	[Marciano L, Viswanath K. Social media use and adolescents’ well-being: A note on flourishing. *Front Psychol*. 2023;14. https://www.frontiersin.org/articles/10.3389/fpsyg.2023.1092109](https://www.zotero.org/google-docs/?yohmcP)

[160.](https://www.zotero.org/google-docs/?yohmcP)	[Mougharbel F, Chaput JP, Sampasa-Kanyinga H, et al. Longitudinal associations between different types of screen use and depression and anxiety symptoms in adolescents. *Front Public Health*. 2023;11:1101594. doi:10.3389/fpubh.2023.1101594](https://www.zotero.org/google-docs/?yohmcP)

[161.](https://www.zotero.org/google-docs/?yohmcP)	[Noel JK, Sammartino CJ, Johnson M, Swanberg J, Rosenthal SR. Smartphone Addiction and Mental Illness In Rhode Island Young Adults. *R I Med J*. 2023;106(3):35-41.](https://www.zotero.org/google-docs/?yohmcP)

[162.](https://www.zotero.org/google-docs/?yohmcP)	[O’Reilly M, Levine D, Donoso V, Voice L, Hughes J, Dogra N. Exploring the potentially positive interaction between social media and mental health; the perspectives of adolescents. *Clin Child Psychol Psychiatry*. 2023;28(2):668-682. doi:10.1177/13591045221106573](https://www.zotero.org/google-docs/?yohmcP)

[163.](https://www.zotero.org/google-docs/?yohmcP)	[Orsolini L, Longo G, Volpe U. The Mediatory Role of the Boredom and Loneliness Dimensions in the Development of Problematic Internet Use. *Int J Environ Res Public Health*. 2023;20(5):4446. doi:10.3390/ijerph20054446](https://www.zotero.org/google-docs/?yohmcP)

[164.](https://www.zotero.org/google-docs/?yohmcP)	[Panayiotou M, Black L, Carmichael-Murphy P, Qualter P, Humphrey N. Time spent on social media among the least influential factors in adolescent mental health: preliminary results from a panel network analysis. *Nat Ment Health*. 2023;1(5):316-326. doi:10.1038/s44220-023-00063-7](https://www.zotero.org/google-docs/?yohmcP)

[165.](https://www.zotero.org/google-docs/?yohmcP)	[Papapanou TK, Darviri C, Kanaka-Gantenbein C, et al. Strong Correlations between Social Appearance Anxiety, Use of Social Media, and Feelings of Loneliness in Adolescents and Young Adults. *Int J Environ Res Public Health*. 2023;20(5):4296. doi:10.3390/ijerph20054296](https://www.zotero.org/google-docs/?yohmcP)

[166.](https://www.zotero.org/google-docs/?yohmcP)	[Seekis V, Lawrence RK. How exposure to body neutrality content on TikTok affects young women’s body image and mood. *Body Image*. 2023;47:101629. doi:10.1016/j.bodyim.2023.101629](https://www.zotero.org/google-docs/?yohmcP)

[167.](https://www.zotero.org/google-docs/?yohmcP)	[Skogen JC, Andersen AIO, Finserås TR, Ranganath P, Brunborg GS, Hjetland GJ. Commonly reported negative experiences on social media are associated with poor mental health and well-being among adolescents: results from the “LifeOnSoMe”-study. *Front Public Health*. 2023;11:1192788. doi:10.3389/fpubh.2023.1192788](https://www.zotero.org/google-docs/?yohmcP)

[168.](https://www.zotero.org/google-docs/?yohmcP)	[Steinsbekk S, Nesi J, Wichstrøm L. Social media behaviors and symptoms of anxiety and depression. A four-wave cohort study from age 10-16 years. *Comput Hum Behav*. 2023;147:107859. doi:10.1016/j.chb.2023.107859](https://www.zotero.org/google-docs/?yohmcP)

[169.](https://www.zotero.org/google-docs/?yohmcP)	[Szlyk HS, Li X, Kasson E, et al. How do teens with a history of suicidal behavior and self‐harm interact with social media? *J Adolesc*. 2023;95(4):797-810. doi:10.1002/jad.12154](https://www.zotero.org/google-docs/?yohmcP)

[170.](https://www.zotero.org/google-docs/?yohmcP)	[Tørmoen AJ, Myhre MØ, Kildahl AT, Walby FA, Rossow I. A nationwide study on time spent on social media and self-harm among adolescents. *Sci Rep*. 2023;13(1):19111. doi:10.1038/s41598-023-46370-y](https://www.zotero.org/google-docs/?yohmcP)

[171.](https://www.zotero.org/google-docs/?yohmcP)	[Tuncturk M, Karacetin G, Ermis C, et al. Internet gaming disorder and problematic internet use: the links between cyberbullying, aggression, loneliness and psychiatric comorbidities. *Psychiatr Danub*. 2023;35(3):395-406. doi:10.24869/psyd.2023.395](https://www.zotero.org/google-docs/?yohmcP)

[172.](https://www.zotero.org/google-docs/?yohmcP)	[Zhu X, Griffiths H, Xiao Z, et al. Trajectories of Screen Time across Adolescence and Their Associations with Adulthood Mental Health and Behavioral Outcomes. *J Youth Adolesc*. 2023;52(7):1433-1447. doi:10.1007/s10964-023-01782-x](https://www.zotero.org/google-docs/?yohmcP)

[173.](https://www.zotero.org/google-docs/?yohmcP)	[Angelini F, Gini G. Differences in perceived online communication and disclosing e‐motions among adolescents and young adults: The role of specific social media features and social anxiety. *J Adolesc*. 2024;96(3):512-525. doi:10.1002/jad.12256](https://www.zotero.org/google-docs/?yohmcP)

[174.](https://www.zotero.org/google-docs/?yohmcP)	[Atwal S, Browne D. Screen Time and Mental Health in Canadian Youth: An Examination of Nationally Representative Data. *Psychol Rep*. 2024;127(4):1678-1704. doi:10.1177/00332941221139995](https://www.zotero.org/google-docs/?yohmcP)

[175.](https://www.zotero.org/google-docs/?yohmcP)	[Azagba S, Ebling T, Korkmaz A. Social media and e-cigarette use: The mediating role of mental health conditions. *J Affect Disord*. 2024;344:528-534. doi:10.1016/j.jad.2023.10.053](https://www.zotero.org/google-docs/?yohmcP)

[176.](https://www.zotero.org/google-docs/?yohmcP)	[Azagba S, Ebling T, Korkmaz A. Social media use and mental health indicators among US adolescents: A population-based study. *J Psychiatr Res*. 2024;176:354-359. doi:10.1016/j.jpsychires.2024.06.043](https://www.zotero.org/google-docs/?yohmcP)

[177.](https://www.zotero.org/google-docs/?yohmcP)	[Barry CT, Moran-Miller K, Levy HF, Gray T. Social media engagement, perceptions of social media costs and benefits, and well-being in college student-athletes. *J Am Coll Health*. 2024;72(8):2938-2947. doi:10.1080/07448481.2022.2142797](https://www.zotero.org/google-docs/?yohmcP)

[178.](https://www.zotero.org/google-docs/?yohmcP)	[Basu N, Blanton MA, Gonzales J, Hendricks KE, Mehari K, Smith PN. Upward social comparisons and suicidal ideation on Facebook: Moderating role of thwarted belongingness. *Psychol Pop Media*. 2024;13(3):513-517. doi:10.1037/ppm0000505](https://www.zotero.org/google-docs/?yohmcP)

[179.](https://www.zotero.org/google-docs/?yohmcP)	[Benedetto L, Rollo S, Cafeo A, et al. Emotional and Behavioural Factors Predisposing to Internet Addiction: The Smartphone Distraction among Italian High School Students. *Int J Environ Res Public Health*. 2024;21(4):386. doi:10.3390/ijerph21040386](https://www.zotero.org/google-docs/?yohmcP)

[180.](https://www.zotero.org/google-docs/?yohmcP)	[Bitto Urbanova L, Holubcikova J, Madarasova Geckova A, Van Dijk JP, Reijneveld SA. Adolescents with emotional and behavioural problems are at higher risk to become excessive or addicted Internet users: is this association moderated by gender? *Eur J Public Health*. 2024;34(2):283-288. doi:10.1093/eurpub/ckae009](https://www.zotero.org/google-docs/?yohmcP)

[181.](https://www.zotero.org/google-docs/?yohmcP)	[Boniel-Nissim M, Marino C, Galeotti T, et al. *A Focus on Adolescent Social Media Use and Gaming in Europe, Central Asia and Canada: Health Behaviour in School-Aged Children International Report from the 2021/2022 Survey.* World Health Organization. Regional Office for Europe; 2024. Accessed November 17, 2024. https://iris.who.int/handle/10665/378982](https://www.zotero.org/google-docs/?yohmcP)

[182.](https://www.zotero.org/google-docs/?yohmcP)	[Brimmel N, Bijttebier P, Eggermont S. Associations between intentions for affective social media content choices and depressive symptoms in adolescence: A cross-sectional investigation of media response styles as moderators. *Psychol Pop Media*. 2024;13(4):550-559. doi:10.1037/ppm0000510](https://www.zotero.org/google-docs/?yohmcP)

[183.](https://www.zotero.org/google-docs/?yohmcP)	[Carlberg Rindestig F, Gillander Gådin K, Semb O, Dennhag I. Unwanted Online Sexual Solicitation Among Young People in a Swedish Psychiatric Sample: Occurrence and Associations with Depression and Anxiety. *J Child Sex Abuse*. 2024;33(5):589-607. doi:10.1080/10538712.2024.2416633](https://www.zotero.org/google-docs/?yohmcP)

[184.](https://www.zotero.org/google-docs/?yohmcP)	[Carter B, Ahmed N, Cassidy O, et al. “There’s more to life than staring at a small screen”: a mixed methods cohort study of problematic smartphone use and the relationship to anxiety, depression and sleep in students aged 13-16 years old in the UK. *BMJ Ment Health*. 2024;27(1). doi:10.1136/bmjment-2024-301115](https://www.zotero.org/google-docs/?yohmcP)

[185.](https://www.zotero.org/google-docs/?yohmcP)	[Carter B, Payne M, Rees P, Sohn SY, Brown J, Kalk NJ. A multi‐school study in England, to assess problematic smartphone usage and anxiety and depression. *Acta Paediatr*. Published online July 31, 2024:apa.17317. doi:10.1111/apa.17317](https://www.zotero.org/google-docs/?yohmcP)

[186.](https://www.zotero.org/google-docs/?yohmcP)	[Charmaraman L, Zhang A, Wang K, Chen B. Sexual Minorities and Loneliness: Exploring Sexuality through Social Media and Gender–Sexuality Alliance (GSA) Supports. *Int J Environ Res Public Health*. 2024;21(3):300. doi:10.3390/ijerph21030300](https://www.zotero.org/google-docs/?yohmcP)

[187.](https://www.zotero.org/google-docs/?yohmcP)	[Çınar Özbay S, Kanbay Y, Firat M, Özbay Ö. The Mediating Effect of Social Anxiety on the Relationship Between Internet Addiction and Aggression in Teenagers. *Psychol Rep*. 2024;127(3):1050-1064. doi:10.1177/00332941221133006](https://www.zotero.org/google-docs/?yohmcP)

[188.](https://www.zotero.org/google-docs/?yohmcP)	[Davis CG, Goldfield GS. Limiting social media use decreases depression, anxiety, and fear of missing out in youth with emotional distress: A randomized controlled trial. *Psychol Pop Media*. Published online 2024:No Pagination Specified-No Pagination Specified. doi:10.1037/ppm0000536](https://www.zotero.org/google-docs/?yohmcP)

[189.](https://www.zotero.org/google-docs/?yohmcP)	[Dyer WJ, Coyne SM, Gale M, Sheppard JA. Who’s most at risk? A person‐centered approach to understanding the long‐term relationship between early social media use and later depression across adolescence. *J Adolesc*. 2024;96(7):1555-1568. doi:10.1002/jad.12362](https://www.zotero.org/google-docs/?yohmcP)

[190.](https://www.zotero.org/google-docs/?yohmcP)	[Eichenberg C, Schneider R, Rumpl H. Social media addiction: associations with attachment style, mental distress, and personality. *BMC Psychiatry*. 2024;24(1):278. doi:10.1186/s12888-024-05709-z](https://www.zotero.org/google-docs/?yohmcP)

[191.](https://www.zotero.org/google-docs/?yohmcP)	[Ganson KT, Pang N, Nagata JM, et al. Screen time, social media use, and weight-related bullying victimization: Findings from an international sample of adolescents. Sacca L, ed. *PLOS ONE*. 2024;19(4):e0299830. doi:10.1371/journal.pone.0299830](https://www.zotero.org/google-docs/?yohmcP)

[192.](https://www.zotero.org/google-docs/?yohmcP)	[Garcia MA, Cooper TV. Social Media Use, Emotional Investment, Self-Control Failure, and Addiction in Relation to Mental and Sleep Health in Hispanic University Emerging Adults. *Psychiatr Q*. 2024;95(4):497-515. doi:10.1007/s11126-024-10085-8](https://www.zotero.org/google-docs/?yohmcP)

[193.](https://www.zotero.org/google-docs/?yohmcP)	[Gingras MP, Brendgen M, Beauchamp MH, et al. Adolescents and Social Media: Longitudinal Links Between Motivations for Using Social Media and Subsequent Internalizing Symptoms. *J Youth Adolesc*. Published online October 2024. doi:10.1007/s10964-024-02097-1](https://www.zotero.org/google-docs/?yohmcP)

[194.](https://www.zotero.org/google-docs/?yohmcP)	[Hamilton JL, Dalack M, Boyd SI, et al. Positive and negative social media experiences and proximal risk for suicidal ideation in adolescents. *J Child Psychol Psychiatry*. 2024;65(12):1580-1589. doi:10.1111/jcpp.13996](https://www.zotero.org/google-docs/?yohmcP)

[195.](https://www.zotero.org/google-docs/?yohmcP)	[Herrmann L, Barkmann C, Bindt C, Hohmann S, Fahrenkrug S, Becker-Hebly I. How social is social media for transgender and gender-diverse youth? Association of online social experiences with internalizing mental health problems. *Eur Child Adolesc Psychiatry*. Published online March 17, 2024. doi:10.1007/s00787-024-02396-9](https://www.zotero.org/google-docs/?yohmcP)

[196.](https://www.zotero.org/google-docs/?yohmcP)	[Idrees B, Sampasa-Kanyinga H, Hamilton HA, Chaput JP. Associations between problem technology use, life stress, and self-esteem among high school students. *BMC Public Health*. 2024;24(1):492. doi:10.1186/s12889-024-17963-7](https://www.zotero.org/google-docs/?yohmcP)

[197.](https://www.zotero.org/google-docs/?yohmcP)	[Kajastus K, Kiviruusu O, Marttunen M, Ranta K. Associations of generalized anxiety and social anxiety symptoms with sleep duration, amount of intense exercise, and excessive internet use among adolescents. *BMC Psychiatry*. 2024;24(1):791. doi:10.1186/s12888-024-06231-y](https://www.zotero.org/google-docs/?yohmcP)

[198.](https://www.zotero.org/google-docs/?yohmcP)	[Karabel M, Onur H, ÓimÓek Ó. Internet addiction and depression: a study among adolescents. *Cir Cir*. 2024;92(4):13448. doi:10.24875/CIRU.24000002](https://www.zotero.org/google-docs/?yohmcP)

[199.](https://www.zotero.org/google-docs/?yohmcP)	[Klinger D, Plener PL, Marboe G, Karwautz A, Kothgassner OD, Dienlin T. Exploring the relationship between media use and depressive symptoms among gender diverse youth: findings of the Mental Health Days Study. *Child Adolesc Psychiatry Ment Health*. 2024;18(1):104. doi:10.1186/s13034-024-00797-x](https://www.zotero.org/google-docs/?yohmcP)

[200.](https://www.zotero.org/google-docs/?yohmcP)	[Kosola S, Mörö S, Holopainen E. Smartphone use and well-being of adolescent girls: a population-based study. *Arch Dis Child*. 2024;109(7):576-581. doi:10.1136/archdischild-2023-326521](https://www.zotero.org/google-docs/?yohmcP)

[201.](https://www.zotero.org/google-docs/?yohmcP)	[Lahti H, Kokkonen M, Hietajärvi L, Lyyra N, Paakkari L. Social media threats and health among adolescents: evidence from the health behaviour in school-aged children study. *Child Adolesc Psychiatry Ment Health*. 2024;18(1):62. doi:10.1186/s13034-024-00754-8](https://www.zotero.org/google-docs/?yohmcP)

[202.](https://www.zotero.org/google-docs/?yohmcP)	[Lee S, Elam K, Lohrmann D, Luo J, Chow A, Seo D. Prospective longitudinal relations among frequent social media use, nicotine vaping and experiencing internalizing mental health problems. *Addiction*. 2024;119(10):1751-1761. doi:10.1111/add.16601](https://www.zotero.org/google-docs/?yohmcP)

[203.](https://www.zotero.org/google-docs/?yohmcP)	[Livet A, Boers E, Laroque F, Afzali MH, McVey G, Conrod PJ. Pathways from adolescent screen time to eating related symptoms: a multilevel longitudinal mediation analysis through self-esteem. *Psychol Health*. 2024;39(9):1167-1182. doi:10.1080/08870446.2022.2141239](https://www.zotero.org/google-docs/?yohmcP)

[204.](https://www.zotero.org/google-docs/?yohmcP)	[Lowthian E, Fee G, Wakeham C, Clegg Z, Crick T, Anthony R. Identifying protective and risk behavior patterns of online communication in young people. *J Adolesc*. 2024;96(2):235-250. doi:10.1002/jad.12270](https://www.zotero.org/google-docs/?yohmcP)

[205.](https://www.zotero.org/google-docs/?yohmcP)	[Markey CH, August KJ, Gillen MM, Rosenbaum DL. An examination of youths’ social media use and body image: Considering TikTok, Snapchat, and Instagram. *J Media Psychol Theor Methods Appl*. Published online 2024:No Pagination Specified-No Pagination Specified. doi:10.1027/1864-1105/a000420](https://www.zotero.org/google-docs/?yohmcP)

[206.](https://www.zotero.org/google-docs/?yohmcP)	[Martín-Cárdaba MÁ, Martínez Díaz MV, Lafuente Pérez P, García Castro J. Smartphone Ownership, Minors’ Well-being, and Parental Mediation Strategies. An Analysis in the Context of Social Media Influencers. *J Youth Adolesc*. 2024;53(10):2202-2218. doi:10.1007/s10964-024-02013-7](https://www.zotero.org/google-docs/?yohmcP)

[207.](https://www.zotero.org/google-docs/?yohmcP)	[Milson R, Madigan DJ. Picture perfect: Perfectionistic self-presentation, Instagram intrusion, and body satisfaction in young women. *J Media Psychol Theor Methods Appl*. Published online 2024:No Pagination Specified-No Pagination Specified. doi:10.1027/1864-1105/a000434](https://www.zotero.org/google-docs/?yohmcP)

[208.](https://www.zotero.org/google-docs/?yohmcP)	[Minich M, Moreno M. Real-world adolescent smartphone use is associated with improvements in mood: An ecological momentary assessment study. Limongi R, ed. *PLOS ONE*. 2024;19(5):e0298422. doi:10.1371/journal.pone.0298422](https://www.zotero.org/google-docs/?yohmcP)

[209.](https://www.zotero.org/google-docs/?yohmcP)	[Murley WD, Oberle CD, Howard KJ, Perrotte JK. Anxiety connects social media use to food and alcohol disturbance and disordered eating when social support is low. *Eat Behav*. 2024;53:101879. doi:10.1016/j.eatbeh.2024.101879](https://www.zotero.org/google-docs/?yohmcP)

[210.](https://www.zotero.org/google-docs/?yohmcP)	[Pazarcikci F. Risk factors for technology addiction in young children ages 2–5 years. *J Pediatr Nurs*. 2024;78:e141-e147. doi:10.1016/j.pedn.2024.06.029](https://www.zotero.org/google-docs/?yohmcP)

[211.](https://www.zotero.org/google-docs/?yohmcP)	[Sarialioğlu A, Oluç T. The Relationship Between Social Media Addiction and Perceived Stress in Adolescents. *J Child Adolesc Psychiatr Nurs*. 2024;37(4):e70000. doi:10.1111/jcap.70000](https://www.zotero.org/google-docs/?yohmcP)

[212.](https://www.zotero.org/google-docs/?yohmcP)	[Sarman A, Çiftci N. Relationship between social media addiction and appearance-related social media consciousness in Turkish adolescents. *J Pediatr Nurs*. 2024;76:99-105. doi:10.1016/j.pedn.2024.02.008](https://www.zotero.org/google-docs/?yohmcP)

[213.](https://www.zotero.org/google-docs/?yohmcP)	[Schmidt-Persson J, Rasmussen MGB, Sørensen SO, et al. Screen Media Use and Mental Health of Children and Adolescents: A Secondary Analysis of a Randomized Clinical Trial. *JAMA Netw Open*. 2024;7(7):e2419881. doi:10.1001/jamanetworkopen.2024.19881](https://www.zotero.org/google-docs/?yohmcP)

[214.](https://www.zotero.org/google-docs/?yohmcP)	[Seekis V, Lawrence R. The effect of TikTok body neutrality content on young women’s self-compassion. *Psychol Pop Media*. Published online 2024:No Pagination Specified-No Pagination Specified. doi:10.1037/ppm0000569](https://www.zotero.org/google-docs/?yohmcP)

[215.](https://www.zotero.org/google-docs/?yohmcP)	[Söyünmez S, Efe YS, Kudubeş AA, Caner N. The predictors of objectified body consciousness among adolescents: Socıal appearance anxiety and social media use. *Arch Psychiatr Nurs*. 2024;51:152-157. doi:10.1016/j.apnu.2024.06.024](https://www.zotero.org/google-docs/?yohmcP)

[216.](https://www.zotero.org/google-docs/?yohmcP)	[Szwedo DE, Davis AA, Fowler C, Mikami AY, Allen JP. Social Media Posts from Friends during Late Adolescence as Predictors of Young Adult Physical Health. *J Youth Adolesc*. 2024;53(4):784-798. doi:10.1007/s10964-024-01945-4](https://www.zotero.org/google-docs/?yohmcP)

[217.](https://www.zotero.org/google-docs/?yohmcP)	[Thai H, Davis CG, Mahboob W, Perry S, Adams A, Goldfield GS. Reducing social media use improves appearance and weight esteem in youth with emotional distress. *Psychol Pop Media*. 2024;13(1):162-169. doi:10.1037/ppm0000460](https://www.zotero.org/google-docs/?yohmcP)

[218.](https://www.zotero.org/google-docs/?yohmcP)	[Tibbs M, Deschênes S, van der Velden P, Fitzgerald A. An Investigation of the Longitudinal Bidirectional Associations Between Interactive Versus Passive Social Media Behaviors and Youth Internalizing Difficulties. A Within-Person Approach. *J Youth Adolesc*. Published online October 12, 2024. doi:10.1007/s10964-024-02093-5](https://www.zotero.org/google-docs/?yohmcP)

[219.](https://www.zotero.org/google-docs/?yohmcP)	[Trekels J. From filters to body positivity: Opposing social media messages and adolescent body image. *Psychol Pop Media*. Published online 2024:No Pagination Specified-No Pagination Specified. doi:10.1037/ppm0000565](https://www.zotero.org/google-docs/?yohmcP)

[220.](https://www.zotero.org/google-docs/?yohmcP)	[Tuck AB, Thompson RJ. Types of social media use are differentially associated with trait and momentary affect. *Emotion*. 2024;24(7):1600-1611. doi:10.1037/emo0001379](https://www.zotero.org/google-docs/?yohmcP)

[221.](https://www.zotero.org/google-docs/?yohmcP)	[Van De Casteele M, Flamant N, Ponnet K, Soenens B, Van Hees V, Vansteenkiste M. Adolescents’ mental health in the social‐media era: The role of offline and online need‐based experiences. *J Adolesc*. 2024;96(3):612-631. doi:10.1002/jad.12286](https://www.zotero.org/google-docs/?yohmcP)

[222.](https://www.zotero.org/google-docs/?yohmcP)	[Visier-Alfonso ME, López-Gil JF, Mesas AE, Jiménez-López E, Cekrezi S, Martínez-Vizcaíno V. Does Socioeconomic Status Moderate the Association Between Screen Time, Mobile Phone Use, Social Networks, Messaging Applications, and Mental Health Among Adolescents? *Cyberpsychology Behav Soc Netw*. 2024;27(11):824-834. doi:10.1089/cyber.2024.0064](https://www.zotero.org/google-docs/?yohmcP)

[223.](https://www.zotero.org/google-docs/?yohmcP)	[Walsh LC, Regan A, Okabe-Miyamoto K, Lyubomirsky S. Does putting down your smartphone make you happier? the effects of restricting digital media on well-being. Mahmoud AB, ed. *PLOS ONE*. 2024;19(10):e0306910. doi:10.1371/journal.pone.0306910](https://www.zotero.org/google-docs/?yohmcP)

[224.](https://www.zotero.org/google-docs/?yohmcP)	[Walsh W, Finkelhor D, Turner H, O’Brien J. Online commercial sexual exploitation of children in a national victim survey. *Psychol Trauma Theory Res Pract Policy*. Published online 2024:No Pagination Specified-No Pagination Specified. doi:10.1037/tra0001821](https://www.zotero.org/google-docs/?yohmcP)

[225.](https://www.zotero.org/google-docs/?yohmcP)	[Wingralek ZJ, Banaszek A, Giermasiński A, et al. Turn on the screen, turn off the loneliness – analysis of risk factors for binge-watching among Polish medical and non-medical students. A web-based cross-sectional study. *Ann Agric Environ Med*. 2024;31(2):264-271. doi:10.26444/aaem/183779](https://www.zotero.org/google-docs/?yohmcP)

[226.](https://www.zotero.org/google-docs/?yohmcP)	[Zsila Á, Shabahang R, Aruguete MS, Bőthe B, Gregor-Tóth P, Orosz G. Exploring the association between Twitch use and well-being. *Psychol Pop Media*. 2024;13(4):620-632. doi:10.1037/ppm0000486](https://www.zotero.org/google-docs/?yohmcP)
